# Supplementary material for: Qualitative study to develop processes and tools for the assessment and tracking of African institutions’ capacity for operational health research
Source: BMJ Open. 2017 Sep 5;7(9):e016660. doi: 10.1136/bmjopen-2017-016660 (PMC5588986; doi:10.1136/bmjopen-2017-016660)
Supplement: Supplementary file 1 [file bmjopen-2017-016660supp001.pdf]

Supplementary Data

Box 1 Key sources used to inform review tools

Optimal institutional research management and support

- Ajai-Ajagbe, P. (2013) #13 Research Management in African Universities. *IAU Horizons* (June) Vol. 19 N° 2 pp 32-33. http://www.acup.cat/sites/default/files/iauhorizonsvol192en1.pdf
- Bates I, *et al*. 2011. Assessing and Strengthening African Universities' Capacity for Doctoral Programmes. *PLoS Medicine.* 8(9).
- Cole DC, Boyd A, Aslanyan G, Bates, I. 2014. Indicators for tracking programmes to strengthen health research capacity in lower- and middle- income countries: a qualitative synthesis. *Health Research Policy and Systems.* **12(17).**
- Dean O, Smith (2011). Managing the Research University. Oxford University Press. ISBN 978-0199793259. Retrieved 2014-08-11.
- Dyason K, Harle J. Research project management in African universities. RIMI4AC. Available online at: http://www.rimi4ac.net/site_media/CONTENT_uploads/ProjectManagement_v1%20[SINGLE%20PAGES].pdf (accessed on 13/08/2014).
- ESSENCE, 2011. Planning, Monitoring and Evaluation. Framework for Capacity Strengthening in Health Research (pdf). TDR/WHO: Geneva. Available online at: http://whqlibdoc.who.int/hq/2011/tdr_essence_11.1_eng.pdf (accessed on 16/05/2014).
- ESSENCE, 2012. Five keys to improving research costing in low- and middle- income countries (pdf). TDR/WHO: Geneva. Available online at: http://whqlibdoc.who.int/hq/2012/TDR_ESSENCE_1.12_eng.pdf?ua=1 (accessed on 16/05/2014).
- Green J, D Langley (2009) Professionalising research management Bristol: HEFCE. A report to HEFCE
- HEFCE (2008) Institutional Benchmarking Template: Establishing and meeting the leadership and development needs of principal investigators
- Kirkland J, Bjarnason S, Stackhouse J. 2006 International research management: benchmarking programme . Report to HEFCE by the Association of Commonwealth Universities. Available online at: http://webarchive.nationalarchives.gov.uk/20100202100434/http://www.hefce.ac.uk/pubs/rdreports/2006//rd11_06/rd11_06.pdf (accessed on 13/08/14)
- Kirkland J, Ajai-Ajagbe, P. 2013. Research management in African universities: from awareness raising to developing structures**.** The association of commonwealth universities (ACU). Available online at: http://carnegie.org/fileadmin/Media/Publications/PDF/ACU_-Research_Management_in_African_Universities_Report_-_2013.pdf (accessed on 02/08/2014, no longer on-line).

- Mirzoev T, Lê G, Green A, Orgill M, Komba A, Esena R, Nyapada L, Uzochukwu B, Made W, Nxumalo N, Gilson L. 2013 Assessment of capacity for Health Policy and Systems Research and Analysis in seven African universities: results from the CHEPSAA project. *Health Policy and Planning*. 10.1093/heapol/czt065
- QAA. 2012. UK Quality Code for Higher Education (pdf). QAA: Gloucester. Available online at: http://www.qaa.ac.uk/Publications/InformationAndGuidance/Documents/B10.pdf (accessed on 16/05/2014).

Cross institution/Country wide research management

- Meek & Olson. Effectiveness of Research and Innovation Management at Policy and Institutional Levels. OECD. (undated) http://www.oecd.org/sti/Effectiveness%20of%20research%20and%20innovation%20management%20at%20policy%20and%20institutional%20levels_Meek%20and%20Olsson.pdf

The structure of the observation checklists, interview guides and the phrasing of some of the questions were based on previously designed evaluation tools, specifically those from the following references:

- Benchmarking Southern African Universities 2014 DRAFT Summary Association of Commonwealth Universities. Available online at: https://www.acu.ac.uk/focus-areas/benchmarking-african-universities (accessed on 13/08/14)
- Kirkland J, Bjarnason S, Stackhouse J. 2006 International research management: benchmarking programme . Report to HEFCE by the Association of Commonwealth Universities. Available online at: http://webarchive.nationalarchives.gov.uk/20100202100434/http://www.hefce.ac.uk/pubs/rdreports/2006//rd11_06/rd11_06.pdf (accessed on 13/08/14)
- Unpublished documents (2014) from RS-DFID Africa Capacity Building Initiative Evaluation: Toolkit (Draft)
- Unpublished documents (2013) from the Royal Society- Leverhulme African Award Evaluation: interview guides, survey methodology, findings report.
- Unpublished documents (2013) from the HRCSI Evaluation 2013: Interview guide package.
- Unpublished documents (2012) from Enhancing Laboratory Capacity Strengthening for NTD control, Deliverable One: tool for capacity gap analysis, interview guides.

Box 2. List of topics/items relevant to university research management and support systems (RMSS), grouped by components

**University Research Strategy**
The university has a research strategy
The research strategy is framed within the overall goals of the institution. The strategy is distinct from but links clearly with, and is complimentary to, other institutional plans, strategies and policies
The research strategy explicitly states its purpose to assist the business of the institution, identifies priorities, and monitor progress
The institution's mechanism for determining research strategy is transparent and widely owned
The institutional research strategy fully involves faculties in its design and implementation, and policies carried out by individual schools or departments are consistent with it
Implementation of the research strategy is overseen by an appropriate member of senior management. The strategy is also backed up by appropriate manpower & resources, to make sure it is implemented
The research strategy has the facility to draw on a range of evaluation mechanisms which might include sources external to the university - such as external peer review including other universities
The Research Management Office [if it exists] is fully involved in the drafting of institutional research strategies in conjunction with other appropriate offices
The research strategy is underpinned by the internal funding mechanisms for research
The research strategy is, as far as possible, responsive to the research funding environment and opportunities (at national, international and regional levels)
The research strategy seeks to add value to existing activity by proactively highlighting new opportunities for internal and external collaboration. The strategy should also promote interdisciplinary research and the development of early career researchers
The research strategy is effectively communicated, monitored, reviewed and developed/refined
Methods for evaluation of the strategy and performance indicators should be established from the outset. Key performance indicators should include a balance of quantitative and qualitative methods
The research strategy should be sufficiently flexible and defined within a reasonable time frame (e.g. 5 years) reviewed regularly, and be capable of evolving in response to events
The strategy should take into account the need for appropriate staff incentives

**Institutional Research Capacity**

The institution has a unit dedicated to research management (Research office)<br>The institution has a research committee to develop strategies to assist the University in meeting its research objectives, identify priorities, advise on stakeholder engagement, monitor research performance, discuss annual department updates, monitor national and international research policy which effect the institution<br>The research unit has an adequate number of Staff to fulfil the needs of researchers<br>Culture where research is valued, accepted, encouraged and enjoyed<br>Ethics committee exists to ensure research conducted is ethical<br>Evidence of accessible guidance to help researchers through the research process including governance and ethics<br>Clear academic honesty guidelines in place<br>Able to meet requirements of GCP and GLP<br>Sufficient facilities are available for research (studying, IT, library, technology, laboratories etc)
**Supporting funding applications**
Institutions have regular, effective and proactive means of informing academics staff on funding opportunities and the strategic directions of funding agencies where possible, these should involve direct communication between the Research Office and individual staff<br>Central research Offices have developed and strategically use key contacts in faculties schools, institutes and departments to facilitate a two way flow of information in funding opportunities and research interest<br>The institution maintains a searchable database on institutions research performance, capabilities and contact, including all past projects and proposals<br>information and current policy from all funders is maintained and communicated as appropriate<br>The Research Office holds regular information and updating sessions and targeted workshops for faculty members and graduate students with the purpose of providing information on funding opportunities, proposal development and the development of collaborative research teams to respond to one-off as well as on-going research opportunities<br>The institution seeks to establish an effective two-way communication strategy between themselves and major sponsors and proactively seek to develop that relationship<br>The institution has clear mechanisms in place to handle internal external enquiries regarding possible research and consultancy opportunities and to monitor the outcomes of these on a regular basis<br>The Research Office actively encourages collaboration between different departments within the institution including senior Academic Office, Public Relations, Marketing and Registry

The institution seeks to develop mechanisms to effectively track and involve alumni working in key positions with current, past and potential sponsors and in government<br><br>The research office actively brings key staff together in response to large scale tender and proposals proposal requests where appropriate and where consistent with research strategy<br><br>Institutions through the Research Office or other appropriate office, ensure that proposals are reviewed by experienced academic and research staff (externally, where appropriate) prior to submission<br><br>Proposals are only submitted with clear support from Head of Department of other appropriate management authority. Key personnel who need to be aware' of the project should be notified.<br><br>The institution approves all proposals before submission and research offices maintain records on the progress of all proposals<br><br>The information gained from previously submitted proposals is used to inform future proposals<br><br>The institution has a clear transparent and widely disseminated formula for determining the full economic cost of any give project, including indirect costs and staff time<br><br>full costing is calculated for each externally funded project even if this is not reflected in the price charged<br><br>All proposed research should be consistent with the institutions overall research strategy<br><br>The institutions provides clear guidance to staff and external sponsors as to which kind of projects and contractual terms are acceptable<br><br>The institution has clear risk assessment procedures for proposed projects which recognise the need to involve several key offices within the institution<br><br>The institution systematically reflects on its progress against its research strategy including regular comparisons with other institutions of similar nature
**Project Management and control**
All project proposals contain explicit statements of how the project will be managed and, where possible and appropriate, provision for the appointment of specialist staff<br><br>Mechanisms are in place to recognise the critical role of Principal Investigators, to ensure that they and other key actors are aware of their roles and responsibilities before commencement of the project and where required, that appropriate training is undertaken.<br><br>Key milestones (including reporting and financial review dates) are agreed with key actors at the outset and updated amongst all those actors throughout<br><br>Key actors, including Principal Investigators and Deans, are provided with regular and up to date project information (including financial, human resources, IP, and commercialization information), through on-line access or regular statements

Information provided to key actors, including Research Officers and Deans, pro-actively highlights any risks and obligations specific to both them and the institution.

Procedures are in place to ensure that all those with access to research are covered by appropriate confidentiality and rights assignment agreements (depending on jurisdiction), particularly those who are covered by a contract of employment with the institution

Appropriate data management policies exist (covering ethical and legal compliance, copyright and IPR issues, data storage, security, sharing and retention)

Appropriate health and safety policies are in place (encompassing staff induction, safety officers, evacuation procedures etc)

Appropriate insurance arrangements are in place for both staff and clinical trials (if applicable)

Mechanisms are in place to ensure that intellectual property both brought to and emerging from research is identified, protected, tracked and signed off at all stages and that staff have access to specialist advice in this regard.

Procedures are in place for the appropriate monitoring of material transfer agreements.

Mechanisms are in place to identify possible delays and monitor expenditure to ensure it is in line with project budgets

The institutions has an explicit consistent framework within which academic units can predict future revenue and expenditure, especially where such income contributes to underpinning core activities

Mechanisms are in place for the disclosure and management of conflicts of interest.

Mechanisms are in place to obtain feedback project sponsors, which can be taken into account in future planning

Formal closure and continuous monitoring processes are in place ensuring that all obligations have been and continue to be met and that opportunities arising from the project are identified.

Training and staff development for research

Evidence of research training needs assessments

Provision of research skills training shaped around skills background and needs of different professional groups

There is availability and use of funds for research skills training for research management staff, researchers and academic staff

There is availability of a range of research skills training for students, research management staff and researchers covering-

Proposal writing, grant application, data analysis and management (including software and qualitative analysis) ethics, health and safety, GCP and GLP, generic research skills (quantitative and qualitative) academic writing and publishing<br><br>Evidence of matching novice and experienced researchers<br><br>Mentorship and supervision structures for students and early career stage researchers and new PI's<br><br>Individual job descriptions support research/institutional objectives<br><br>Policies are in place to support recruitment and contract negotiation for new support staff<br><br>Staff skills and abilities are matched to research needs<br><br>Seminar programmes relating to research undertaken<br><br>The research management structure and policies form a core element of induction programmes for new academic and technical staff as well as new postgraduate students.<br><br>Research strategy, policy and management issues form a core element of ongoing professional development programmes for mid-career and senior academic staff.<br><br>Staff in leadership roles (e.g. Deans) are offered appropriate instruction in research strategy, policy and management, as well as being involved in discussion of good practice within the institution<br><br>The Research Office maintains effective ongoing relationships with internal clients at all levels (faculty, department, individuals) with a view to supporting research staff and understanding their needs.<br><br>Performance measures for research management are established and are widely available/disseminated.<br><br>The institution makes provision for appropriate incentives to enhance the research activity of new and emerging researchers. Such incentives might include conference grants and other start-up funding.<br><br>Policies for providing incentives for staff research activity are transparent, easy to understand and consistent across the institution.<br><br>Career development opportunities<br><br>Career pathways exist for researchers
**Teaching capacity to support research**
Number of (half as a minimum) full-time academic staff as active and recognised contributors to subject associations, learned societies and relevant professional bodies.<br><br>Number of (third as a minimum) academic staff with recent (i.e. within the past three years) personal experience of research activity (including external examination, review panels, collaborative research)

Number of (third as a minimum)academic staff engaged in research or other forms of advanced scholarship<br><br>The outcomes of external scrutiny exercises undertaken by bodies such as the Quality Assurance Agency for Higher Education, the funding councils and professional and statutory bodies are carefully considered and actioned.
**External promotion of research**
Collaborations exist with external organisations (institutions, businesses, government, NGO's)<br><br>The institution is able to conform to the requirements of multiple funding agencies<br><br>Number of joint posts with other academic institutions<br><br>The institution has a clear strategy in place for all forms of intellectual property management<br><br>Clear regulations are in place to determine the ownership of intellectual property by and between staff, students and third parties. These regulations are effectively disseminated throughout the institution and externally<br><br>Academic departments and research projects are systematically monitored to identify emerging intellectual property at an early stage.<br><br>The institution establishes a register of intellectual property assets and pro-actively manages and maintains it at all stages of development and exploitation<br><br>Clear policy mechanisms are in place to govern the distribution of revenues from intellectual property between the university and other key stakeholders.<br><br>The institution's research communication strategy is consistent with the institution's overall strategy and underpins the core missions of the institution, particularly in relation to the integration of research, education and service.<br><br>There is a clear understanding of the roles and responsibilities of the different offices/officers responsible for research communication and good channels of communication exist between all these actors.<br><br>The institution pro-actively identifies projects (at various stages) and outcomes that are aligned with the university's priorities and are particularly suitable for external dissemination<br><br>The institution has a programme of events, such as launches, to profile major achievements or projects which relate to the strategic objectives and any priority research themes of the institution.<br><br>The institution has clear criteria for the type of work most likely to generate good publicity, and guidance on how to avoid poor publicity, and makes this information available to staff.<br><br>The institution has a clear strategy and procedures with regard to handling crisis communications and ensures these are disseminated to every level.<br><br>The institution seeks to make key research findings accessible to a wider audience, through the use of research summaries, expert guides and speakers lists, produced in suitable lay language and in

publicly accessible formats so as to engage public understanding of the core mission of the institution (including inter-institutional partnerships).<br><br>The institution has established clear mechanisms to review and reward the performance of departments and research groups in the area of dissemination, which are integrated with an incentivisation policy providing a variety of incentives.<br><br>Mechanisms are in place for staff to report their dissemination activity. Such mechanisms maximise research kudos and academic excellence and are consistent with any reporting requirements to external organisations<br><br>The institution provides assistance and systematic training programmes for staff in handling the media, and specific assistance in the drafting of press releases and publicity materials.<br><br>The institution facilitates the participation of researchers, particularly early career researchers, in international conferences and other fora to present their research findings and raise their profile<br><br>Where possible, dissemination outputs of staff are captured in a centrally managed integrated digital repository, linked to any central research activity database, which is made available to all units of the institution<br><br>The institution has a clear branding policy which is consistent with the research communication strategy.<br><br>The institution's web portal reflects the institution's core mission and strategy and is strategically and systematically managed as a key tool for promoting research to the broader community.
**National Research Uptake**
Ability of link policy to research and practice<br><br>Number of evidence based policies<br><br>Number of evidence based development interventions<br><br>Number of plans and policies to support research<br><br>Existence of national standards (accreditation, quality assurance) regarding the evaluation of research institutes<br><br>Existence of scientific councils with transparent and efficient systems in place to evaluate and disburse competitive research funds<br><br>Level of funding of research by the government<br><br>Researcher salary on par or above other countries in region

Box 3. Overall semi-structured interview suite of questions, by RMSS component

Biography of interviewee

What is your current position within this institution?

How long have you held this position?

How long have you worked at this institution?

What is your role in research within this institution?

1. Research strategies and policies

The institution

How many staff and students are there at this institution?

What is the percentage of income from a) teaching and b) research?

Is there core funding for research? How much? How is it disbursed?

How many PhD students are registered a) with your institution and b) externally?

Is there a university officer/directorate responsible for research? Do they have terms of reference?

How does this institution's research outputs compare to other comparable institutions? How do you measure this?

Strategies

Do you have a university research strategy?

What are the main themes/components of the strategy?

Does it link to a) national and b) other institutional strategies?

How is it disseminated internally and externally?

What are the research strengths at this institution?

Are strategies revised? How often? What were any major changes?

What are the strategic priority research areas? How were they decided? How are researchers and externally funded projects encouraged to focus on these areas?

Was any baseline information (e.g. a SWOT analysis) used to inform the strategy?

Who was involved in setting the strategy? What was the process?

2. Institutional Support Services and Infrastructure

Research management

Is there a university research committee? What do they do?

Is there a research support office? What do they do? (E.g. identify opportunities, help with application process, and ensure compliance with funder's requirements)

How are you made aware of research funding opportunities? (at national, international and regional levels)

How do you keep track of publications/presentations/conferences/grant applications produced/ department?

ICT (also see data management in section 4)

Is there adequate Wi-Fi, broadband speed, video conferencing and skype facilities for researchers? Do they pay for this?

Can they access the IT systems from home?

Do you purchase computers etc. on their behalf, or make recommendations? Do you set them up? Help with software? Is there any charge for this service?

How are files and information backed up? (e.g. offsite servers)

Library

How do staff and students access peer reviewed and grey literature? Are there any regular training courses offered?

How is access to e-resources and hard copy books/journals managed between the ICT unit and the library?

Laboratories

What research laboratories and field sites are available to use for research purposes at this institution?

What type of research studies can be supported by the laboratories (e.g. HPLC for pharmacokinetics; genomics/sequencing; insectory etc.)

Are the laboratories enrolled in external quality assurance systems?

Do the laboratories have international accreditation?

Do the laboratories follow Good Laboratory Practice guidelines?

Are there backup generators? Surge protection?

What sample storage facilities do you have? Are they temperature controlled and monitored?

What are the policies and processes governing transfer of samples to external institutions?

3. Supporting funding applications

What are the mechanisms for identifying external funding opportunities? Does the research office help with this? (E.g. is there a ‘grants identification’ officer?)

Is there any support to help PIs prepare funding proposals? (E.g. getting documents together, preparing/checking budgets, submitting proposals)

What is the mechanism for collating information on all proposals submitted? Is there a searchable database of submitted projects and whether they were successful?

What is the process for submitting proposals? Is there a formal sign off and if so by whom?

Do proposals have to have input or approval from finance/accountants prior to submission? What do they look for? How do you make sure that overheads are included and the costings are correct (e.g. salaries, equipment)?

Does the university use external advice (e.g. legal) at any stage during the process?

Do you have any way of comparing your research performance with other institutions?

4. Project management and control

What systems are in place to monitor the progress of each project? (E.g. against milestones)

Ethics

How is this managed in the university as a whole? Is this done at the university or at the faculty level?

Is ethics committee membership GCP-compliant?

Are there guidelines about how the ethics committee functions?

Are there guidelines for researchers about the ethics process?

Are there guidelines relating to academic honesty and plagiarism?

Financial

Who provides financial reports to funders? Who has specialist knowledge of each funders’ reporting requirements?

How often are financial reports made to PI’s (frequency, method, feedback loop?)

How do departments predict and plan future research revenue and expenditure?

How does the university ensure that project expenditure remains in line with the budget?

Legal

What is the process for minimising risks regarding financial and contractual terms? Is legal advice available? Who accesses this and when? (ie during the contract signing process or only if there is a problem)?

How are appropriate insurance arrangements organised (particularly for field staff and clinical trials)

What regulations are in place to determine the ownership of intellectual property by and between staff, students and third parties? How are these regulations disseminated throughout the institution and externally?

If what ways do you identify emerging intellectual property in your academic departments and on-going research projects?

Have you established a register of intellectual property assets? How are these managed and maintained?

What policies/mechanisms are in place to govern the distribution of revenues from intellectual property between the university and other key stakeholders?

Data management

Are there research data management guidelines and/or policies for data protection and storage?

How is research data backed up and secured? How are routine office and research documents (e.g. draft publications, guidelines/protocols etc.) backed up and secured?

Who is responsible for these systems? Are PIs charged for this service?

Do you provide help for PIs to complete Data Management plans to funders?

What are the mechanisms for managing data ownership, data security, licensing for re-use, data sharing, reuse of third-party data, restriction of data sharing (prior to publishing or seeking patents, retaining/destroying data?

Clinical work/trials questions:

Does the university acts as a sponsor for clinical trials?

Is there a clinical trials office? What does it do?

How do you do clinical monitoring? Have any audits about this been conducted and if so what were the key findings?

5. Human Resource Management for Research

Are job descriptions available for researchers and support staff?

Is there an induction process for new employees?

What are the processes for promotion for a) researchers and b) support staff (e.g. administrators, laboratory scientists)?

What mentorship and supervision structures exist for students and early career stage researchers and new PI’s?

What career pathways are there are for a) researchers and b) research support staff?

Is there any ongoing professional development programme and does this cover research skills?

Do you know about the MCDC career development groups (CDGs)?

How do the MCDC career development groups (CDGs) fit into/complement institutional systems?

Are these career development activities embedded in institutional structures?

Do you think they are helpful? Should they be institutionalised? Why/why not?

Human resources

What Policies/strategies are in place for Human resource development of a) researchers/scientific staff, b) admin staff (including training, retention, tenure track, funding)

Do you have a formal induction process for new employees? Is there a special one for researchers?

Are there health and safety policies? (E.g. staff induction, safety officers, evacuation procedures etc.)

How are training needs identified? (E.g. staff training needs assessments). Is HR responsible for providing and/or recording any research training (e.g. GCP/GLP training, proposal writing, project management, supervision)?

How are training opportunities identified and funded? Is there a core budget for training and how is it allocated?

What proportion of research posts are a) core funded and b) project funded?

Are you involved in all new appointments? Do you advise PIs on the institution's procedures governing the employment of staff?

Is career guidance given to PhD students, post-docs and other researchers?

How are post-docs absorbed into the workforce?

What is the process and turnaround time for recruiting and appointing new research staff?

Do you make more internal or external research appointments?

Does your institution offer the possibility of short-term bridging funding to retain research staff during hiatus periods between grants?

Can you describe what Performance measures are used for research management and how these are reported?

Are there joint posts with other academic institutions? How do they work and are they effective?

6. Human Resource Development for Research

Is training available on

- Research design (epidemiology, stats, social science, health systems)
- Ethics, health and safety, GCP and GLP
- Data analysis and management (including software and qualitative analysis)
- Academic writing and publishing
- Proposal writing, grant application
- Teaching and education
- Leadership and management

Are there facilities and fora (e.g. seminars, journal club, staff exchanges) for researchers to discuss their work regularly with each other?

Is a tracking system in place for PhD students? How many supervisors do PhD students have? How many students do PhD supervisors have?

Are there minimum standards in place about the level of supervision to be given?

7. External promotion of research

Do you have longstanding research collaborations with external organisations? Examples?

Do you have a research communication strategy? A research communication unit?

Who is responsible for research communication? Do researchers perceive that this is their responsibility?

Does the institution have a research profile on its website?

How do you make key research findings accessible to a non-academic audience (e.g. research summaries in lay language and in publicly accessible formats)?

Do you have a programme of events, such as launches, to profile major achievements or projects

Do you provide advice to staff about how to deal with the media (e.g. how to generate good publicity and avoid poor publicity)?

What strategy and procedures are communicated to staff with regard to handling crisis communications and how are these disseminated?

Are there incentives for departments and research groups in the area of dissemination?

8. National research engagement

What level of funding for research is provided by the government?

Are there national policies and plans regarding health (and other) research?

How does the institution engage with policy makers?

Are there national scientific councils that evaluate and disburse competitive research funds? Do they have transparent and efficient systems?

What are the mechanisms by which research from your institution influences policy and practice?

Are there national standards (accreditation, quality assurance) regarding the evaluation of research institutes?
