# Supplementary material for: Qualitative study to develop processes and tools for the assessment and tracking of African institutions’ capacity for operational health research
Source: BMJ Open. 2017 Sep 5;7(9):e016660. doi: 10.1136/bmjopen-2017-016660 (PMC5588986; doi:10.1136/bmjopen-2017-016660)
Supplement: Author's manuscript [file bmjopen-2017-016660.draft_revisions.pdf]

BMJ Open

BMJ Open is committed to open peer review. As part of this commitment we make the peer review history of every article we publish publicly available.

When an article is published we post the peer reviewers' comments and the authors' responses online. We also post the versions of the paper that were used during peer review. These are the versions that the peer review comments apply to.

The versions of the paper that follow are the versions that were submitted during the peer review process. They are not the versions of record or the final published versions. They should not be cited or distributed as the published version of this manuscript.

BMJ Open is an open access journal and the full, final, typeset and author-corrected version of record of the manuscript is available on our site with no access controls, subscription charges or pay-per-view fees (http://bmjopen.bmj.com).

If you have any questions on BMJ Open's open peer review process please email editorial.bmjopen@bmj.com

BMJ Open

Process and tools for the assessment and tracking of African institutions' capacity for health research

Journal:	*BMJ Open*
Manuscript ID	bmjopen-2017-016660
Article Type:	Research
Date Submitted by the Author:	02-Mar-2017

Complete List of Authors:	Wallis, Selina; Liverpool school of tropical medicine, International health<br>Cole, Donald; University of Toronto, Dalla Lana School of Public Health<br>gaye, oumar<br>mmbaga, blandina<br>mwapasa, victor<br>tagbor, harry<br>bates, imelda

**Primary Subject Heading**:	Global health
Secondary Subject Heading:	Global health
Keywords:	Research activities, Institutional practices, Systems analysis, Organization & Administration, Evaluation

SCHOLARONE™
Manuscripts

Process and tools for the assessment and tracking of African institutions' capacity for health research

Selina Wallis, Donald C Cole, Oumar Gaye, Blandina T. Mmbaga, Victor Mwapasa, Harry Tagbor, Imelda Bates

Corresponding author's name, address, telephone/fax numbers and e-mail address;

Imelda Bates, Liverpool School of Tropical Medicine, Pembroke Place, Liverpool L3 5QA

Tel: 0151 705 3115; Imelda.bates@lstmed.ac.uk

Keywords [MeSH] Research activities, Institutional practices, Systems analysis, Organization & Administration, Evaluation

Abbreviated Running Title - Assessing research management systems

Abstract

Objectives: The capacity of institutions in LMICs to produce international quality health research depends on many inter-linking 'systems' factors. We aimed to develop and test an evidence-informed process for conducting a holistic assessment of universities' health research management systems (RMSS) and for tracking interventions to address gaps.

Setting: Four African universities

Participants: Seventy-six diverse university staff and post-graduate students

Intervention/methods: An evidence-informed 'benchmark' was developed and used to itemise all components of a university's health RMSS. Data on all components were collected during site visits to four universities in sub-Saharan Africa using mixed methods research and diverse tools (interview guides, document reviews and facilities observation guides). Using the benchmark, gaps in health research capacity were identified and action plans developed to remedy gaps. Progress against indicators was tracked and common challenges and successes identified.

Results: Common gaps in health research capacity included: no accessible research strategy, a lack of central tracking of research activities, outputs or impact, and lack of quality assurance for proposal submissions and contracts.

Conclusions: Identification of each institutions' strengths and weaknesses against the same evidence-informed benchmark enabled them to identify gaps in in their health research systems, to develop prioritised action plans, to justify resource requests to fulfil the plans, and to track progress in strengthening research systems. Feedback indicated that the process was comprehensive and generated practical actions, several of which were no-cost. The regular follow-up helped to maintain focus on activities to strengthen health research capacity in the face of challenges. The use of standard benchmark, approach and tools enabled inter-institutional comparisons in health research capacity to be made which has accelerated production of evidence about the science of research capacity strengthening. Research capacity gaps that were common to several institutions could be a 'smart' investment for governments and health research funders.

For peer review only - http://bmjopen.bmj.com/site/about/guidelines.xhtml

Trial registration: none

Strengths and limitations of this study

- This study uses mixed methods research to generate primary, prospective, longitudinal data about the baseline status of health research systems in four African institutions, and tracks changes in research capacity against pre-determined indicators
- The use of the same benchmark and research approach across different institutions enables comparisons to be made so common challenges can be identified; these could be effective targets for investment
- The main limitations for the study were that the limited follow up time did not allow for demonstration of the long-term sustainability of changes to research systems and, because our study was designed to provide a broad overview of an institution's RMSS, it did not explore particular components in depth
- Institutions found the evaluation process to be comprehensive and helpful since in addition to advancing the science of research capacity strengthening it generated practical actions and progress indicators, and facilitated inter-institutional comparison and bench-marking

Source of funding: This work was supported by a grant from the Wellcome Trust, UK, to the London School of Hygiene and Tropical Medicine MCDC project (http://www.mcdconsortium.org/phd-programme.php). The funders had no role in study design, data collection and analysis, decision to publish, or preparation of the manuscript.

Competing interests
We have read and understood BMJ policy on declaration of interests (http://www.bmj.com/about-bmj/resources-authors/forms-policies-and-checklists/declaration-competing-interests) and declare that we have no conflicts of interest

Word count: 4228 excluding tables, figures/diagrams and references

For peer review only - http://bmjopen.bmj.com/site/about/guidelines.xhtml

Process and tools for the assessment and tracking of African institutions' capacity for health research

INTRODUCTION

Importance of research for development

Health research has been acknowledged to play a key role in progress towards the Sustainable Development Goals.[1] Strong research institutions and skilled researchers are essential for low- and middle-income countries to generate evidence for their own health policies and to make progress in achieving their health related goals.[2, 3] Investments in health research capacity, can provide positive returns by promoting evidence-informed policy and practice in the health system,[2] although implementation[4] and estimation of returns can be challenging.[5] The first African Ministerial Conference on Science and Technology in 2003 recognised that "There is strong evidence that using research evidence to inform policy and practice leads to benefits which contribute to socioeconomic development"[6] and participating countries committed to spend at least 1% of their GDP on research and development by 2010.[7] Few African countries have managed to meet this target and Kenya, Mozambique, Senegal and Uganda all have more than 40% of their research and development financed from abroad.[7]

Lack of research/researchers in LMICs especially Africa

Although the average growth rate of scientific production in Africa is faster than that of the world as a whole, African Union countries only produce 2% of the world's total scientific output.[8] Egypt, Kenya, Nigeria, and South Africa produce the largest number of publications from Africa.[7] This is a reflection of the small numbers of researchers in Africa and decades of under-investment in research institutions. Most countries in sub-Saharan Africa (SSA) have less than 500 researchers per million inhabitants (e.g. Tanzania 35, Ghana 39, Malawi 50, Senegal 361) compared to over 4000 per million inhabitants in the UK and North America.[9] There are numerous disincentives to pursuing a research career in many African countries including heavy teaching loads, weak organisational research systems, lack of national research leadership, limited access to scientific information, slow internet connections and inadequate physical facilities including libraries and laboratories.[10]

Attempts to address weak capacity for health research in Africa

Resources to guide development of health research capacity have been available for at least a decade[11] but outdated and ineffective models for strengthening capacity persist [12] African research institutions have historically faced numerous challenges.[13] The ability to produce international quality health research depends not only on developing a critical mass of African researchers, but also on providing them with a conducive environment in which to do research and progress their careers.[14, 15] International funders have responded by supporting strengthening of national systems and structures for health research[16] and in boosting the capacity of low- and middle-income country universities in research governance and management.[17] However, despite longstanding calls for more robust evaluations of capacity development,[11] the evidence needed to inform effective implementation and evaluation of programmes for strengthening health research capacity remains weak.[18, 19] Furthermore, the lack of clearly defined goals and baselines against which to evaluate the success of research capacity strengthening programmes makes it difficult to track their progress and impact.[20]

Development funders and policy makers are calling for a "significant re-think of the approach to capacity development".[21] They stress the need for an inter-disciplinary approach which recognises

For peer review only - http://bmjopen.bmj.com/site/about/guidelines.xhtml

the complexity, fluidity and non-linearity in human systems, a systematic perspective, and acknowledgement of relationships between capacity at the individual, institutional and wider societal levels.[22, 19] To promote a more purposeful and strategic approach to strengthening health research capacity in low- and middle-income countries, a group of international funders have produced guidance about developing shared principles and indicators,[23] and for evaluating outcomes and impacts of health research capacity strengthening interventions. Putting these guidelines into practice at the organizational level is challenging since little is known about what information matters for strengthening research capacity, and how and why this varies in different institutional contexts.

Purpose

The purpose of our study was to develop and test an evidence-informed process that could be used 1) to conduct a baseline assessment of health research management and support systems (RMSS) in African universities and 2) to document actions taken to address identified gaps. As institutions implemented these actions, we sought to identify common difficulties they encountered. This information would help not only the institutions, but also external agencies and national governments, to more effectively target and monitor their contributions to strengthening institutional and hence, national health research capacity. The assessment process covered all the components needed for a university to generate, manage and disseminate health research of international quality.

Despite earlier work on research management benchmarking,[24] no single document existed which detailed all the systems needed in a university to foster, support and manage international quality health research. Hence it was necessary to develop a comprehensive description of the components of an 'optimal' scenario[19] as a benchmark against which the baseline assessment could be compared.[22, 25] We describe the process of using best available evidence to generate this benchmark as a health Research Management and Support Systems (RMSS) list and to craft tools for collecting baseline data in each of the universities. We share our experience of using the tools to identify institutions' RMSS capacity gaps, the early results on tracking the universities' progress and challenges in strengthening their RMSS and senior researchers' experience with the RMSS assessment process.

METHODS

Partner universities

We worked with four African universities which were partners in the Malaria Capacity Development Consortium (MCDC 2008-2015, http://www.mcdconsortium.org/) funded by the Bill and Melinda Gates Foundation and the Wellcome Trust. MCDC supported African scientists to undertake high-quality malaria research and to enhance the health research capacity of their home institutions. In particular, MCDC aimed to strengthen the capacity of the African universities to provide academic, administrative and financial support to generate health research of international quality despite differences in geography, size and maturity of their research infrastructure.

The entry point for our study into each of the universities was the department (or centre) in which MCDC's collaborating principal investigator was located. These departments had been established between 1957 and 1991; all had active malaria research programmes and offered postgraduate training. The departments' universities were located in west (Anglophone and Francophone), east and southern Africa and at the time of the study had between 6,000 and 60,000 registered students.

For peer review only - http://bmjopen.bmj.com/site/about/guidelines.xhtml

Generation of a list of research management and support system components

In order to conduct a holistic assessment of the African universities' health RMSS it was necessary to first create a benchmark by identifying all the components and related best practice required for the optimal functioning of such systems.[19] As no single document available detailed all these components, we drafted an initial list of components by itemising all activities that occur within a project cycle and by identifying all the support mechanisms that are required to conceive, generate and monitor research and to ensure that research findings are used to inform national health policies and practices. The list guided a mapping of relevant information using internet searches. The search for relevant global publications included academic articles, and grey literature such as guidelines and regulations governing research aspects of higher education institutions (Supplementary file Box 1). We also interrogated websites of agencies relevant for each of the themes, and read their reports and documents and any references included therein and consulted with researchers, grants managers and research finance officers until saturation was achieved. We aimed to cover aspects of the institutional capacity needed to provide optimal academic, administrative and financial support for health research activities from the perspectives of the Dean or Principal of the institution, faculty research support staff and researchers at different career stages.

Items on the list were grouped into components which were simultaneously adjusted and expanded to encompass all the aspects of RMSS, with no duplication across components (Supplementary file Box 2). The final RMSS components were:

1. Research Strategies and Policies
2. Institutional Support Services and Infrastructure
3. Supporting Funding Applications
4. Project Management and Control
5. Human Resource Management for Research
6. Human Resource Development for Research
7. External Promotion of Research
8. National Research Engagement

Development of tools for data collection

The most appropriate methods to be used for collecting data on each of the components and their associated items during subsequent visits to the universities were.[26] The primary data collection tool was a guide for semi-structured interviews with different cadres of university staff, supplemented by a list of facilities to be visited at the institutions (i.e. library, IT suite, laboratories) and a list of documents to be reviewed (i.e. strategies, policies, regulations, handbooks).

Inclusion of the entire master list of items for every component in every semi-structured interview would have been impractical and inappropriate. Since each interviewee would have knowledge of specific aspects of RMSS in their institution, combinations of questions were selected from an overall suite (Supplementary file Box 3) to construct focused interview guides for different cadres of interviewees (i.e. Heads of Department/Institute Deans or Principals; senior researchers; staff with research support responsibilities such as administration, finance, human resources, communications, ethics and laboratories). For example, questions for laboratory technicians, but not

For peer review only - http://bmjopen.bmj.com/site/about/guidelines.xhtml

for other cadres, dealt with equipment maintenance. We ensured that all items from the master list were covered across the set of cadre-specific interview guides.

The data collection tools (lists and interview guides) were reviewed by all members of the research team and adjustments were made to reduce redundancy. Additional changes were made after the first university visit and minor revisions were made during the visit to the second university. After this, no more revisions were required, so this version was used for the two subsequent visits.

Baseline data collection during university visits

Pre-visit briefings were conducted by Skype with the MCDC principal investigator in each of the African universities, to explain the purpose and process of the visits and to schedule interviews. They were provided with the data collection tools in advance of the visits so they were aware of the range and type of information that would be sought. Subsequently, 3-4 day visits to each of the four African universities were conducted by 2-3 members of the research team between September and November 2014.

As far as possible, all data collected during the visits was obtained from at least two independent sources to enhance validity.[27] Interviewees were asked if any aspects of research systems had not been covered by the interview questions and, as a result, procurement procedures were added to the questions for the second and subsequent visits. During each interview, interviewees were asked to propose feasible actions that could be taken to overcome any of the challenges or gaps in research support systems that they mentioned.

Notes from the interviews were typed up within a few hours of each interview, checked against audio-recordings of the interviews (available if interviewees gave permission), and final versions were verified among the site visit team. Information from observation of facilities and review of documents was used to elaborate and verify data from the interviews. A consultation meeting was held at the end of each visit for all available interviewees to share preliminary findings about strengths and gaps identified in the institutional RMSS. In keeping with the principles of inter-disciplinary team reflexivity[28] and of pooling internal and external assessments,[29] we used the meetings to check the accuracy of the findings, to discuss the reasons for discrepancies, to generate and prioritise proposed actions, and to ensure that such actions were deemed feasible by institution staff.

Baseline Data analysis

A framework analysis approach was used to manage and analyse the multi-disciplinary information generated from the site visits about institutions' 'baseline' research systems.[30] Data were entered into a matrix which had a row for each of the eight components. Columns for topics within each of the RMSS components that emerged from the interviews were constructed using deductive (i.e. based on the topics/items grouped under each component from the scoping review) and inductive (i.e. unexpected new topics that emerged from the information collected) approaches. Use of the matrix facilitated identification of emerging patterns and comparison of the strengths and weaknesses in each institution's research systems. Following the site visits, findings were presented in a draft report which was reviewed by the MCDC principal investigators in consultation with their institutional colleagues, before being finalised. To respect confidentiality, the final reports were only shared with the MCDC secretariat and the institutions themselves. An anonymised 'overview' report was produced and made publically available which summarised commonalities and differences in RMSS across all institutions and highlighted innovative RMSS practices.[31]

For peer review only - http://bmjopen.bmj.com/site/about/guidelines.xhtml

Follow-up interviews for tracking progress and obtaining feedback on the process

Information about progress and challenges in addressing gaps in the institutions' health RMSS was obtained through 2-5 Skype and telephone interviews with the MCDC principal investigators in each institution over fifteen months until May 2016. Each interview lasted 20-40 minutes and covered the gaps and actions identified in the relevant intuitional baseline report. Progress on each action, means by which progress had been achieved, and any challenges experienced, were documented. During the interviews the principal investigators were asked to comment on whether the process had been helpful, and if so how, and which aspects could be improved in the future and to reflect on their role as research manager practitioners.[32] These comments were organized into themes, and quotes reflective of each theme were selected to convey the principal investigators' perspectives in their own words.

Information obtained about progress and challenges around actions in the baseline report were mapped against the eight RMSS components using a pre-prepared matrix and analysed using a framework analysis approach. The research team broadly assessed whether the institutions collectively had made 'good', 'moderate' or 'little/no' progress in addressing the gaps in each component of their research support systems. This helped understanding about which components of research support systems all four universities found most easy to address and which they found hardest. A report outlining progress and challenges was drafted for each institution and reviewed by each principal investigator.

RESULTS

Baseline situation

In total 76 interviews were conducted (18-20/university), 65 documents/resources (12-20/university) were reviewed, and facilities observed included libraries, research laboratories and study spaces. The gaps in RMSS that were common (i.e. occurred in at least three of the four universities), and proposed actions that emerged during the on-site visits to address these gaps, were categorised by RMSS component (table 1).

Progress in strengthening universities' RMSS

All of the universities had made some progress in addressing gaps in their research support systems, and there were some common successes and challenges (table 2). Although MCDC provided some institutions with limited funding to address some of these gaps, many of the actions, such as re-organisation of management structures or in-house training, did not require additional funds.

Overall, little or no progress was made in Research Policies and Strategies, External Promotion of Research, and National Research Engagement; moderate progress was made in Institutional Support Services and Infrastructure and Human Resource Management and Development for Research; and good progress in Funding Applications and Project Management and Control. Examples of innovative practices and problem-solving were identified for each component (table 2).

The process of assessing and tracking strengthening of RMSS

The process of assessing and providing feedback on institutional RMSS used in the study was universally viewed as a positive and constructive way to raise awareness of the importance of

For peer review only - http://bmjopen.bmj.com/site/about/guidelines.xhtml

strengthening research support systems and to catalyse broader institutional engagement with these topics. Relevant interviewee comments included:

"Senior staff are really engaging with this. They understand the importance of the programme"

"The project definitely helped to raise awareness of all the challenges we are facing, that we need more funds and to improve the environment; it highlighted difficulties and that all the partners are now really interested in helping African institutions. It enabled us to start some concrete actions and now we have institutional buy in, now they are engaged and committed to go further"

An area for improvement was in ensuring that important documents provided to institutions, such as drafts of the research capacity assessments, were produced in French as well as English language.

"It would help if the report was in French, with logos, stamp and signature - an official version. Otherwise a translation is not taken seriously"

The comprehensive nature of the assessments and data collection tools provided confidence that all key aspects of research support systems had been covered during the process and helped stakeholders to prioritise and justify their future budgeting and funding requests.

"It was very useful to get an overview of the whole system from an outside team"

"A piecemeal approach would not be effective at all. We need to look at each area. We can then leverage funding ….and use this [assessment] to make sure every area is funded."

The collaboration between an external team and stakeholders within the institutions brought additional benefits in terms of impartiality and reduction in bias, which would not have been possible with an exclusively internal review team. Seeking opinions from multiple perspectives and the involvement of external team, helped to overcome internal sensitivities.

"It stimulated honest and fair discussion between us all…… It demonstrated our strengths as well as weaknesses. Everyone said it didn't say anything we didn't know but as an outside organisation produced it there were no biases. That's why everyone has agreed we need to move forward"

"Certain areas that the overall report helped when I was presenting the sensitive issues that there are common problems - instead of feeling hopeless, we felt we were doing better in some areas. We knew … that here are political issues. If the recommendation had come from within that could have caused issues"

Addressing gaps in research support systems is a complex undertaking and regular contact with the external team to track progress was helpful for keeping the focus on priorities and maintaining momentum.

"The follow up process was helpful to keep me focused on understanding the changes occurring across the college and in all areas of research management."

"On-going follow up was helpful to keep on track with forward movement"

For peer review only - http://bmjopen.bmj.com/site/about/guidelines.xhtml

DISCUSSION

Process and Tools

We have demonstrated that is it possible to construct and implement a coherent, evidence–informed process for assessing and tracking programmes to strengthen institutions' health RMSS. The comprehensive data collection tools drew on current approaches and evidence from several disciplines including research management, education and organisational systems.[33, 34, 35] It has parallels with others' efforts[36] to construct assessment tools to improve the quality of indicators and processes for measuring health research capacity strengthening.[20] The assessment process was systematic yet flexible enough to accommodate the complexity and fluidity of health RMSS, across a range of African universities. The assessment process acknowledged the influence of inter-relationships between individual, institutional and wider societal levels on the 'research ecosystem' (i.e. researchers and their institutions, funders and governments who support research, policymakers who use research, and communication specialists who share and discuss the findings with a broad audience).[37] The way in which the assessment process was conducted, particularly the findings from the baseline assessments and the collaborative identification of actions to address health RMSS gaps, was universally viewed as positive, and is consistent with others' experience in reviewing health research capacity.[36, 4] In addition the institutional assessments helped to raise awareness of the importance of strengthening RMSS[18] and to catalyse multi-disciplinary engagement in improving RMSS across the institutions.[38]

Such assessments would be difficult for exclusively internal teams to undertake since they may struggle to gain timely access to senior university officials and could be influenced by sensitivities and politics within the institutions. A partnership between senior institutional researchers, who intimately understood the structural, financial and political context, and an external team, who were impartial and experienced in such assessments, was therefore essential to maximise assessment validity and contribution to learning.[18] Such insider-outsider assessments have also been used in examining research ethics systems.[29] The transferability of the RMSS assessment tools and processes across geo -political and institutional boundaries means they could be usefully deployed in the increasingly common model of research consortia.[24] Of note is the need to produce reports for non-Anglophone universities in the country's dominant language, since language barriers are known to be a critical handicap in scientific collaborations and in engaging senior university officials.[39]

Tracking Progress/Challenges

Although there are numerous publications of retrospective evaluations of research capacity strengthening efforts, prospective tracking of progress is far less common.[40] We applied an established five step process for assessing baseline status and prospectively tracking changes in health research capacity.[18] The researchers perceived the process as constructive since it helped to maintain focus and momentum within the institution, and provided an opportunity to introduce and share innovative approaches to problem-solving at each institution and for each RMSS component. Most institutions had made the best progress in areas that were primarily under the control of the collaborating senior researchers' departments, such as involving finance officers and managers in developing research proposals, and providing training and resources for managing grants. Much of this progress was achieved with limited or no additional funds. This may therefore be a useful indicator of what might be achieved by other research institutions in Africa who have minimal external support.

For peer review only - http://bmjopen.bmj.com/site/about/guidelines.xhtml

Gaps in health research capacity that were generally found to be most the challenging to remedy depended on university-wide changes. Examples included embedding research training, which was usually non-sustainably linked to projects, within university systems, and ensuring laboratories were accredited and underpinned by sustainable financing models. Most challenging of all were the lack of systems for communication and dissemination of research outputs and for using research to influence health policies and programmes. This lack of institutional knowledge exchange capacity to promote research uptake in Africa has been noted by others.[41]

Limitations of the study

Our study was designed to provide a broad overview of an institution's health RMSS, and therefore could did not explore particular components in depth. Other instruments and guidelines are available to do this including: Good Financial Grants Practice,[42] for researchers' development framework,[34] Octagon for research ethics capacity,[29] 'stepwise' laboratory accreditation,[43] and DRUSSA for research uptake.[44] The MCDC principal investigators varied in their seniority, influence and social capital[45] (i.e. the norms and networks that enable people to act collectively) which may have affected the thoroughness of the assessment phase, as well as the extent of progress especially in implementing university-wide actions. The lack of a theory of change[46] for the broader MCDC programme meant that explicit articulation of a common set of outcomes and pathway to change for strengthening RMSS was lacking.[47] Tracking information was generally not independently verified, as it was based on Skype or phone interviews with the MCDC principal investigators. The follow up time was 15 months which is too short to be able to demonstrate longer term impact of such a process on health RMSS. Hence, we regard our prospective tracking as an initial experience which could be used to guide a more fulsome, prospective evaluation.

Contributions to an emerging science

Momentum is gathering around a new global science on research capacity strengthening which draws on implementation research,[48] research evaluation processes[5] and mixed methods research methodologies.[49] Our effort is consonant with this developing global science, addressing the area of health RMSS with an explicit and comprehensive set of assessment tools, embedded in a collegial, collaborative process. Similar to a small but growing number of colleagues engaged in contributing to the science-base for research capacity strengthening, we are sharing our tools in a peer-review forum, so that others can apply and adapt them for assessing their own or others' university's RMSS. Linking collaborative RMSS assessments of gaps with collegial generation of actions to address those gaps, and jointly tracking progress on chosen actions and challenges prospectively constitutes a more rigorous approach to health research capacity strengthening than has been common to date.[20] In addition, documentation of innovative problem-solving by African institutions is crucial to counter deficit-focused narratives, facilitate sharing among resource-constrained institutions, and facilitate universities' role as agents of change.[50] An additional benefit of using a systematic, common approach to strengthening institutional health research capacity is that it provides evidence for external agencies and governments about better targeting of efforts to make institutions in Africa globally competitive research leaders.

IMPLICATIONS

Research capacity outputs need to be recognised as of equivalent value to research outputs[12] and therefore need a rigorous scientific basis. Our experience in developing and applying an assessment and tracking framework can facilitate similar initiatives in other research oriented institutions in

For peer review only - http://bmjopen.bmj.com/site/about/guidelines.xhtml

lower and middle income countries and their respective consortia. The identification and sharing of RMSS components that are commonly problematic could guide national governments to target their resources towards these weakest components. At the supra-national level, the use of our tools and process, and sharing of the results more widely, enable comparisons to be made across institutions and countries. Such analyses would not only contribute to the science of health research capacity strengthening, by enabling common research approaches and tools to be applied in different contexts and by validating findings on common capacity gaps, but also provide guidance to international health and research funders about 'smart' investment of resources. Sharing of problem-solving innovations in RMSS among universities and research institutes with similar resource constraints through such organizations as the African Academy of Sciences is an important more immediate opportunity. Finding ways to share such innovations widely beyond health, for example through inter-disciplinary study tours or joint workshops for researchers and research support staff, is imperative for fostering collaborations for RMSS strengthening, and hence health system strengthening more broadly.

Contributorship statement

SW: Liverpool School of Tropical Medicine, Liverpool, UK
contributed to study design, data collection, analysis and final paper

DCC: Professor, Dalla Lana School of Public Health, University of Toronto, Canada
contributed to study design, data collection, analysis and final paper

OG: Professor, Cheikh Anta Diop University, Dakar, Senegal
contributed to data collection and final paper

BTM: Kilimanjaro Clinical Research institute, Kilimanjaro Christian Medical Centre and Kilimanjaro Christian Medical University College Moshi, Tanzania
contributed to data collection and final paper

VM: College of Medicine, University of Malawi, Blantyre, Malawi
contributed to data collection and final paper

HT: Kwame Nkrumah University of Science and Technology, Kumasi, Ghana
contributed to data collection and final paper

IB: Liverpool School of Tropical Medicine, Liverpool, UK
led study design, contributed to data collection, analysis and led drafts of final paper

Acknowledgements
We acknowledge the collaboration of staff of participating universities, which have remained anonymous for reasons of confidentiality of the findings. Thanks to other members of the study and administration team, Ema Kelly and Vicki Doyle of Capacity Development International, Dorte Holler Johansen from the University of Copenhagen, Denmark, and Lorelei Silvester and Denise Wellings from LSTM for their valuable contributions to this study

Data sharing statement
No additional data are available

For peer review only - http://bmjopen.bmj.com/site/about/guidelines.xhtml

All data related to this study are included in this submission, either in tables in the manuscript or in supplementary files

For peer review only

For peer review only - http://bmjopen.bmj.com/site/about/guidelines.xhtml

References

1. World Health Organization. Health in 2015: from MDGs, Millennium Development Goals to SDGs. Sustainable Development Goals. Switzerland: World Health Organization. Available: http://apps.who.int/iris/bitstream/10665/200009/1/9789241565110_eng.pdf?ua=1 (accessed 27 February 2017)
2. Koon A, Nambiar D, Rao D. 2012. Embedding of research into decision-making processes. Geneva: Alliance for Health Policy and Systems Research, WHO
3. Bennet S, Akua Agyepong I, Sheikh K, Hanson K, Ssengooba F, Gilson L. 2011. Building the field of health policy and systems research: an agenda for action. *PLoS Medicine* 8: 1023
4. Hawkes S, Aulakh BK, Jadeja N, Jimenez M, Buse K, Anwar I, Barge S, Odubanjo MO, Shukla A, Ghaffar A. 2016. Strengthening capacity to apply health research evidence in policy making: experience from four countries. *Health Policy and Planning* 31(2): 161-70
5. Neufeld V, Cole DC, Boyd A, Njelesani D, Bates I, Hanney SR. 2014. Perspectives on Evaluating Global Health Research for Development: A Background Paper. Taking stock of current practice and ways to improve it. For GHRI-IDRC. 79 pp. p9-11. www.ccghr.ca/wp-content/uploads/2014/07/EGHR4D-final-paper.pdf (accessed 27 February 2017)
6. Newman K. 2014. What is the evidence on the impact of research on international development. London: DFID. https://assets.publishing.service.gov.uk/media/57a089aced915d622c000343/impact-of-research-on-international-development.pdf (accessed 27 February 2017)
7. NEPAD Planning and Coordinating Agency (NPCA) 2014. African Innovation Outlook 2014, NPCA, Pretoria. http://www.un.org/africarenewal/sites/www.un.org.africarenewal/files/AIO_2_Final%20Product[2].pdf (accessed 27 February 2017)
8. Neupane B. 2015. A more developmental approach to science. UNESCO science report: towards 2030 http://en.unesco.org/unesco_science_report (accessed 27 February 2017)
9. United Nations Educational, Scientific and Cultural Organisation (UNESCO) Institute for Statistics. Number of Researchers per million inhabitants by country. <http://www.uis.unesco.org/ev.php?URL_ID=3755&URL_DO=DO_TOPIC&URL_SECTION=201>. converted by ChartsBin statistics collector team 2011, Number of Researchers per million inhabitants by Country, ChartsBin.com, <http://chartsbin.com/view/1124>.
10. Vogel I. 2011. Research Capacity Strengthening: Learning from Experience. UKCDS, London. http://www.ukcds.org.uk/sites/default/files/content/resources/UKCDS_Capacity_Building_Report_July_2012.pdf (accessed 27 February 2017)
11. Nuyens Y. 2007. 10 best resources for health research capacity strengthening. *Health Policy and Planning* 22(4): 274-276
12. Franzen SRP, Chandler C and Lang T. Health research capacity development in low- and middle-income countries: reality or rhetoric? A systematic meta-narrative review of the qualitative literature. *BMJ Open* 2017;7:e012332
13. Sawyerr A. 2004 African universities and the challenge of research capacity development. *JHEA/RESA* 2(1): 213–42.
14. Miiro GM, Oukem-Boyer OOM, Sarr O, Rahmani M, Ntoumi F, Dheda K, Pym A, Mboup S, Kaleebu P. 2013. EDCTP regional networks of excellence: initial merits for planned clinical trials in Africa. *BMC Public Health* 13: 1

For peer review only - http://bmjopen.bmj.com/site/about/guidelines.xhtml

15. Cole DC, Johnson N, Mejia R, Mccullough H, Turcotte-Tremblay A-M, Barnoya J, Falabella Luco S. 2016. Mentoring health researchers globally: Diverse experiences, programmes, challenges and responses. Global Public Health 11(9): 1093-108
16. Enoch J. 2015a. Rapid mapping of international funders' research capacity strengthening priorities. UKCDS. http://www.ukcds.org.uk/sites/default/files/content/resources/UKCDS%20rapid%20RCS%20mapping%20for%20DFID.pdf (accessed 27 February 2017)
17. Enoch J. 2015b. Health Research Capacity Strengthening: A UKCDS Mapping. Published by UKCDS. http://www.ukcds.org.uk/our-work/health-research-capacity-strengthening-a-ukcds-mapping (accessed 27 February 2017)
18. Bates I, Boyd A, Aslanyan G, Cole DC. 2015a. Tackling the tensions in evaluating capacity strengthening for health research in low-and middle-income countries. Health Policy and Planning 30: 334-344.
19. Bates I, Boyd A, Smith H, Cole DC. 2014. A practical and systematic approach to organisational capacity strengthening for research in the health sector in Africa. *Health Research Policy and Systems* 12: 11
20. Cole D C, Boyd A, Aslanyan G, Bates I. 2014. Indicators for tracking programmes to strengthen health research capacity in lower-and middle-income countries: a qualitative synthesis. *Health Research Policy and Systems* 12: 17
21. Department for International Development (DFID) 2008. Research strategy 2008-2013 Working Paper Series: Capacity Building. https://assets.publishing.service.gov.uk/media/57a08b8ee5274a31e0000c0a/ResearchStrategyWorkingPaperfinal_capacity_P1.pdf ((accessed 27 February 2017)
22. Ghaffar DA, Ijsselmuiden C, Zicker F. 2008. Changing mindsets: Research capacity strengthening in low-and middle-income countries, Council on Health Research for Development (COHRED). http://www.who.int/tdr/publications/documents/changing_mindsets.pdf (accessed 27 February 2017)
23. ESSENCE (Revision 2016). Planning, Monitoring and Evaluation. Framework for Capacity Strengthening in Health Research. WHO. www.who.int/tdr/publications/Essence_frwk_2016_web.pdf (accessed 27 February 2017)
24. Kirkland J, Bjarnason S, Stackhouse J, Day R. 2006. International research management: Benchmarking programme. Report to the HEFCE by the Association of Commonwealth Universities.
25. Altbach PG, Salmi J. 2012. *The road to academic excellence: the making of world-class research universities*. Directions in development; human development. Washington DC: World Bank
26. Denzin NK, Lincoln YS. 2008. Collecting and interpreting qualitative materials. Published by Sage.
27. Golafshani N. 2003 Understanding reliability and validity in qualitative research. *The Qualitative Report* 8: 597-606
28. Cohen D, Crabtree B. Reflexivity. Qualitative Research Guidelines Project. http://www.qualres.org/HomeRefl-3703.html (accessed 27 February 2017)
29. Hyder AA, Zafar W, Ali J, Ssekubugu R, Ndebele P, Kass N. 2013 Evaluating institutional capacity for research ethics in Africa: a case study from Botswana. *BMC Medical Ethics* 14: 31
30. Gale NK, Heath G, Cameron E, Rashid S, Redwood S. 2013. Using the framework method for the analysis of qualitative data in multi-disciplinary health research. BMC Medical Research Methodology 13: 117

For peer review only - http://bmjopen.bmj.com/site/about/guidelines.xhtml

31. Bates I, Cole DC, Doyle V, Kelly E, Holler Johansen D. 2015b. Strengthening Research Management and Support Systems (RMSS) in African Universities. Overview Report for Four Universities to MCDC. September - October 2014. Accessed 27 February 2017 at http://www.mcdconsortium.org/sites/www.mcdconsortium.org/files/MCDC%20RMSS%20overview%20report%20FINAL%209jan15_0.pdf
32. Cunliffe AL. 2004. On becoming a critically reflexive practitioner. *Journal of Management Education* 28(4): 407-426
33. Quality Assurance Agency 2010. Code of practice for the assurance of academic quality and standards in higher education. http://www.bbk.ac.uk/linkinglondon/resources/apel-credit-resources/pub_Nov2010_QAA_COP_section_2_HE_guidance.pdf (accessed 27 February 2017)
34. Vitae, 2010. Research Development Framework. https://www.vitae.ac.uk/vitae-publications/rdf-related/researcher-development-framework-rdf-vitae.pdf/view (accessed 27 February 2017)
35. UK Research Councils, Arts and Humanities Research Board Joint statement of the Research Councils'/AHRB's skills training requirements for research students. http://www.rcuk.ac.uk/documents/skills/statementofexpectation-pdf/ (accessed 27 February 2017)
36. Jessani N, Lewy D, Ekirapa-Kiracho E, Bennett S. 2014. Institutional capacity for health systems research in East and Central African schools of public health: experiences with a capacity assessment tool. *Health Research Policy and Systems* 12: 21
37. Wellcome Trust. undated. Strengthening research ecosystems in Africa and Asia. https://wellcome.ac.uk/what-we-do/our-work/research-ecosystems-africa-and-asia. (accessed 27 February 2017)
38. Mashaah T, Hakim J, Chidzonga M, Kangwende RA, Naik Y, Federspiel N, Fiorillo S, Scott J, Gomo E. 2014. Strengthening research governance for sustainable research: Experiences from three Zimbabwean universities. *Academic Medicine* 89: S69-S72
39. Hwang K. 2013. Effects of the language barrier on processes and performance of international scientific collaboration, collaborators' participation, organizational integrity, and inter-organizational relationships. *Science Communication* 35(1): 3–31
40. Wirrman-Gadsby E. 2011. Research capacity strengthening, donor approaches to improving and assessing its impact in low- and middle-income countries. *International Journal of Health Planning and Management* 26: 89–106.
41. Ayah R, Jessani N, Mafuta EM. 2014. Institutional capacity for health systems research in East and Central African schools of public health: knowledge translation and effective communication. *Health Research Policy and Systems* 12: 20
42. African Academy of Sciences. Undated. Good Financial Grants Practice – Standardising, simplifying and strengthening governance of grant funding. http://aasciences.ac.ke/aas/en/academy/academy-pages/good-financial-grants-practice/ (accessed 27 February 2017)
43. World Health Organization Regional Office for Africa. Undated. WHO Guide for the Stepwise Laboratory Improvement Process Towards Accreditation in the African Region (with checklist). http://www.who.int/tb/laboratory/afro-slipta-checklist-guidance.pdf (accessed 27 February 2017)
44. Development Research Uptake in Sub-Saharan Africa (DRUSSA). Undated. Strategies for effective research uptake communication and impact. http://www.drussa.net/index.php?option=com_content&view=article&id=2788%3Astrategies-

For peer review only - http://bmjopen.bmj.com/site/about/guidelines.xhtml

for-effective-research-uptake-communication-and-impact&catid=211%3Atools-a-tips-open&Itemid=297&lang=en (accessed 27 February 2017)

45. Woolcock M, Narayan D. 2000. Social capital: Implications for development theory, research, and policy. *The World Bank Research Observer* 15: 225-249
46. Vogel I. 2012. Review of the use of 'Theory of Change' in international development. Report for the UK Department for International Development (DFID). August. https://assets.publishing.service.gov.uk/media/57a08a5ded915d3cfd00071a/DFID_ToC_Review_VogelV7.pdf (accessed 27 February 2017)
47. Boyd A, Cole DC, Cho DB, Aslanyan G, Bates I. 2013 Frameworks for evaluating health research capacity strengthening: a qualitative study. *Health Research Policy and Systems* 11(1): 46.
48. Peters DH, Adam T, Alonge O, Agyepong IA, Tran N. 2013. Implementation research: what it is and how to do it. *BMJ* 347: f6753
49. Creswell JW, Plano Clark VL. 2010. 2nd edition. Designing and Conducting Mixed Methods Research. Published by Sage https://us.sagepub.com/en-us/nam/designing-and-conducting-mixed-methods-research/book233508
50. Hansen JA, Lehmann M. 2006. Agents of change: universities as development hubs. *Journal of Cleaner Production* 14: 820-829

For peer review only

For peer review only - http://bmjopen.bmj.com/site/about/guidelines.xhtml

Table 1. Consolidated key gaps in research management and support systems (RMSS) and proposed actions, by RMSS component

Gaps	Proposed actions
**Research Strategies and Policies**	
• No research strategy or not available or publicised<br>• Lack of central tracking of research activities	• Departments/universities need an accessible research strategy with polices and guidelines to support its implementation<br>• Electronic research management support systems are needed to track proposals and projects and to document research income and disbursement including overheads
**Institutional Support Services and Infrastructure**	
• Lack of research support offices and/or insufficient coordination between departments and university levels<br>• Inadequate resourcing and lack of clarity about the role and long term financial sustainability of research support offices<br>• Research laboratory facilities are not accredited and lack over-arching planning to harmonise equipment purchase and maintenance across multiple short term projects<br>• Unclear relationship between hard copy library facilities and increasing use of e-resources	• The roles and relationships between university level research coordination and research support offices at Faculty or College level need to be clarified<br>• The strategy for research support offices at Faculty or College level needs to be clarified and mechanisms found for long term sustainability and buy-in by the researchers<br>• Achieve international laboratory accreditation for the institution’s own laboratories; harmonise research laboratories’ activities with those of affiliated organisations and establish clear processes and costs for researchers wishing to access these facilities<br>• Pro-actively plan the future of book libraries in the context of the shift to increasing use of e-resources, including their possible integration with ICT facilities
**Supporting Funding Applications**	
• Insufficient quality assurance checks and signing off processes for proposal submissions or contracts which could put the institution at risk of contractual or intellectual property issues	• Set up mechanisms for timely, multi-disciplinary (e.g. finance, legal, ICT, laboratory, library, procurement) input into proposal development<br>• Set up a formal process for quality assurance and authorisation of proposals before submission and for tracking the outcome of submissions
**Project Management and Control**	
• Senior researchers spend a substantial proportion of time on administrative, procurement and	• Establish an electronic research information system to systematically manage and track all aspects each project including the project agreement, protocol,

For peer review only - http://bmjopen.bmj.com/site/about/guidelines.xhtml

other issues that could be more effectively taken on by non-academic professional staff<br>• Lack of systems for tracking financial spend against budget for projects risks under- or over-spend<br>• Unclear lines of responsibility between researchers and finance officers regarding financial tracking and reporting	budgets, funding requirements, accounting and audit, and to maximise recoupment of overheads<br>• Establish a formal project approval process for successful applications, including and contract review and sign off<br>• Encourage researchers to include and budget for experienced administrators to help reduce the time they spend on project administration and to actively include other relevant inputs such as procurement expertise<br>• Provide joint training in financial management for researchers and finance officers and increase clarity and understanding about their various roles and responsibilities in relation to each other, the institution and the research funders
**Human Resource Management for Research**	
• Lack of clarity on contractual arrangements, and therefore institutional responsibility, for short-term project staff<br>• Poorly defined, or non-existent, career paths for non-academic professionals such as ICT, library and administrative staff<br>• No formal post-doctoral career posts for researchers	• Strengthen HR skills and structures so that they can better support researchers and research projects, and to ensure that project staff are university employees with access to the protection and facilities of the institution where this is not currently the case<br>• Formalise career tracks for research support staff<br>• Formal post-doctoral training programmes need to be established to develop and retain talented researchers
**Human Resource Development for Research**	
• No coordinated, institutionalised programmes for induction or research skills training for researchers; reliance on projects to provide training means focus is on technical skills rather than generic skills, such as leadership and research communication<br>• Training offered at university level (e.g. computer skills, literature searching) poorly publicised and utilised by researchers	• Provide a formal induction programme and training needs assessment for new research staff<br>• Establish an institutional programme of skills training for researchers, possibly through a dedicated unit, that includes non-technical skills such as leadership, supervision and project management<br>• Improve incorporation of existing training opportunities (e.g. provided by library and ICT staff) into a core skills training programme for researchers
**External Promotion of Research**	
• Promotion of research activities and successes by the universities not prioritised although widely recognised as important	• Review research section of universities' website to ensure information is current and that hyperlinks are working<br>• Consider setting up a unit specifically to enhance the visibility of institutional and/or departmental

For peer review only - http://bmjopen.bmj.com/site/about/guidelines.xhtml

	research activities and outputs<br>• Provide training in research communication to improve researchers' ability to write "jargon-free" communications such as press releases and policy briefs
**National Research Engagement**	
• Insufficient publicising of institutional research outputs in influencing national and international policy making and programming	• Explore options for improving researchers' ability to impact on national health research priorities and practices<br>• Universities and departments should systematically document and showcase national and international uptake and utilisation of the research findings they have generated

For peer review only

Table 2. Progress on actions, challenges and innovative problem-solving during 2015-16, by RMSS* component and institution

Compo-nent [Overall progress]	Institution 1	Institution 2	Institution 3	Institution 4	Examples of innovative practices and problem-solving
**Research Strategies and Policies** [little/no progress]	University level strategy being approved but College level not yet developed.<br>A new university-wide research database now in place. A small university research fund has been set up.	Consultation on the strategy has taken place. A draft document is being written to submit for management approval.<br>Research management software has been installed to manage the research database	A research grant office is planned for early 2016 and a research database will be created within this office.	Strategy is on-hold as wide-scale institutional constitutional change is underway.<br>[post-project note: *Strategic plan 2017-2022 was approved December 2016*]	• Through a consultative process and workshop involving researchers, university officials, research support staff and representatives from external national agencies, the Ministry of Health and international research consortia, a research strategy was agreed upon based on a SWOT analysis
**Institutional Support Services and Infrastruc-ture [moderate progress]**	Work is on-going to coordinate inputs to proposals and grant management by PIs and finance staff. Workshops took place in 2015.	MoU and SoP in development for grant related activities.<br>ICT policies for marketing and staff engagement are being developed.<br>Sustainability planning for lab resources and budgeting is underway.	Instalment of high speed internet (DSL) at the Department of Biology, the PhD dedicated space and the Faculty library.<br>Steps towards formal accreditation of quality assurance processes and certification for the department labs has begun.	Progress has been made in establishing a research support office (grants unit) with administrators and financial staff appointed.	• A Laboratory Committee has been formed and inventoried all key laboratory resources within the College and affiliated research institutions
**Supporting**	A university-wide	Information about the	Implementation of a	Administrators from the	• Production of a "how-to"

For peer review only - http://bmjopen.bmj.com/site/about/guidelines.xhtml

**Funding Applica-tions [good progress]**	database has been implemented.<br><br>The finance office is now involved in proposal development and joint training has taken place.	research support services has been disseminated.<br><br>"How-to" guideline on developing research proposals with a budget framework and checklists has been launched.<br><br>A research careers development webpage has been developed by the institution with materials for fledgling researchers	research grant office and recruitment of a research coordinator has been agreed.	research/grants office have been assisting the PIs during processes of proposal development, registering projects and tracking implementation and management.<br><br>The Grant office has been registered to several "calls application portals" and a database of researcher interests is being created.	flow chart outlining the steps needed to develop research proposals<br>• A framework for calculating a proportion of staff time to be included in proposals for grants management has been established<br>• Topic-specific research groups have been formed which can respond to funding calls and collaboration opportunities<br>• A legal unit has been set up to guide the contract review process and a contract template and checklist developed
**Project Manage-ment and Control [good progress]**	Contract review, sign off and risk management procedures have been strengthened with contracts signed off by Provosts or Deans of Colleges.<br><br>Need for in-house training for admin to support PIs identified.	New systems for pre- and post-award management have been developed.<br><br>A new policy for compulsory post-award "Grants Management" inductions for all successful Principal Investigators is available.<br><br>A Grants administrator has now been appointed. A legal unit has been set up as part	SOPs for expenses and account management have been developed and validated with PIs, finance and administrative officers	Project management procedures are now overseen by an administrator who manages the grant and liaises with the PI on the implementation of the project.<br><br>The financial management system PASTEL is being used but staff need training to upload project data.	• Standard operating procedures for expenses and account management have been developed jointly by researchers, finance and administrative officers.<br>• Quarterly meetings between finance officers and researchers are used to address financial

For peer review only - http://bmjopen.bmj.com/site/about/guidelines.xhtml

		of research support services			management of project grants
**Human Resource Manage-ment for Research [moderate progress]**	HR recruitment policies and procedures for project staff have been reviewed and developed.<br>A new staff development programme has been launched.<br>On-going meetings are continuing to discuss the need to develop a post-graduate program	A training needs assessment for research support staff has been completed and training plans developed.<br>Procedures for hiring post-doctoral fellows have been established and terms and conditions of service defined.	Project management training courses and cross site visits for administrative staff and early career researchers have strengthened human resource management.<br>Staff needs for personal development have been identified but there is yet to be a plan to address these.	A team has been formed to develop a research scale based on a national scale for Researchers.<br>Post- doctoral programmes are not yet institutionalised	• Career Development Centres have been established and stocked with training resources<br>• Human resources staff are included in planning for research staff development
**Human Resource Develop-ment for Research [moderate progress]**	Work is still on-going to plan a formal research-based induction process for project staff and fledgling researchers.<br>Mentorship training and pairing has taken place.	Work has begun towards a comprehensive competency- based capacity building programme for junior and senior researchers.<br>A research capacity gap validation workshop and a training needs assessment for research staff have been conducted. An annual research training calendar has been developed.	Progress made towards adaptation and institutionalisation of the doctoral research courses for researchers.<br>An external funder is working with the institution to plan personal development and leadership courses.	The role of HR teams in research staff development has been reviewed and defined.<br>More work needs to be done to build a structured programme of capacity development for new and existing staff in research skills	• An annual research training calendar has been developed for research staff based on a formal training needs assessment<br>• Regular training is provided by the university on the roles and responsibilities of research leaders, administrators and finance staff throughout the grant life cycle
**External Promotion**	Some training and mentoring in research communication provided	Research profiles of faculty are being posted on their	The Faculty of Medicine website has been updated with current	The institution has a strong external collaborative network including an annual	• An attractive annual university research report

For peer review only - http://bmjopen.bmj.com/site/about/guidelines.xhtml

**of Research** [little/no progress]	by an external agency.<br><br>Some improvement of the research projects on the College website but more needs to be done to encourage researchers to add their details.<br><br>The academic board are to incorporate research uptake and the management of research outputs, into the university's centralised research policy.	webpages.<br><br>The separate research office website is under construction.<br><br>There are plans for a Knowledge Management Unit to help achieve better visibility of research activities.	research projects.<br><br>The recruitment of a communication officer is planned, who will be responsible for supporting the publicising and dissemination of research activities and uptake.	PhD symposium.<br><br>The planned new research strategy will embed research dissemination and uptake as a high priority area.<br><br>Work is ongoing to restructure the website	which chronicles recent research activities and highlights individuals, departments and colleges has been produced and is publically available
**National Research Engage-ment** [little/no progress]	Mechanisms still need to be established at College and departmental level to promote, monitor and record communication and to share opportunities for engagement between projects.<br><br>The institution's work with multi-national partners on national research engagement will feed into the College's research strategy.	Key national health policy making forums have been targeted with specifically-designed materials.<br><br>A local travel grant system is being established to support researchers to attend national meetings.<br><br>A template has been developed for annual research reports, to highlight successes, failures and opportunities for policy engagement	Currently participating in national tailored monitoring and evaluation training courses for medical district officers and health supervisors where research results and experience will be shared	Several researchers attend a national research conference in 2015 to disseminate their projects.<br><br>Promotion of their research into policy and practice is not yet stated in the research strategy as a core element of a researchers' responsibility	• A university research uptake strategy has been published<br>• University hosts a meeting of leading thinkers in science, policy, industry and civil society in Africa

*RMSS = research management and support system

For peer review only - http://bmjopen.bmj.com/site/about/guidelines.xhtml

For peer review only

For peer review only - http://bmjopen.bmj.com/site/about/guidelines.xhtml

Supplementary Data

Box 1 Key sources used to inform review tools

Optimal institutional research management and support

- Ajai-Ajagbe, P. (2013) #13 Research Management in African Universities. *IAU Horizons* (June) Vol. 19 N° 2 pp 32-33. http://www.acup.cat/sites/default/files/iauhorizonsvol192en1.pdf
- Bates I, *et al*. 2011. Assessing and Strengthening African Universities' Capacity for Doctoral Programmes. *PLoS Medicine.* 8(9).
- Cole DC, Boyd A, Aslanyan G, Bates, I. 2014. Indicators for tracking programmes to strengthen health research capacity in lower- and middle- income countries: a qualitative synthesis. *Health Research Policy and Systems.* **12(17).**
- Dean O, Smith (2011). Managing the Research University. Oxford University Press. ISBN 978-0199793259. Retrieved 2014-08-11.
- Dyason K, Harle J. Research project management in African universities. RIMI4AC. Available online at: http://www.rimi4ac.net/site_media/CONTENT_uploads/ProjectManagement_v1%20[SINGLE%20PAGES].pdf (accessed on 13/08/2014).
- ESSENCE, 2011. Planning, Monitoring and Evaluation. Framework for Capacity Strengthening in Health Research (pdf). TDR/WHO: Geneva. Available online at: http://whqlibdoc.who.int/hq/2011/tdr_essence_11.1_eng.pdf (accessed on 16/05/2014).
- ESSENCE, 2012. Five keys to improving research costing in low- and middle- income countries (pdf). TDR/WHO: Geneva. Available online at: http://whqlibdoc.who.int/hq/2012/TDR_ESSENCE_1.12_eng.pdf?ua=1 (accessed on 16/05/2014).
- Green J, D Langley (2009) Professionalising research management Bristol: HEFCE. A report to HEFCE
- HEFCE (2008) Institutional Benchmarking Template: Establishing and meeting the leadership and development needs of principal investigators
- Kirkland J, Bjarnason S, Stackhouse J. 2006 International research management: benchmarking programme . Report to HEFCE by the Association of Commonwealth Universities. Available online at: http://webarchive.nationalarchives.gov.uk/20100202100434/http://www.hefce.ac.uk/pubs/rdreports/2006//rd11_06/rd11_06.pdf (accessed on 13/08/14)
- Kirkland J, Ajai-Ajagbe, P. 2013. Research management in African universities: from awareness raising to developing structures**.** The association of commonwealth universities (ACU). Available online at: http://carnegie.org/fileadmin/Media/Publications/PDF/ACU_-Research_Management_in_African_Universities_Report_-_2013.pdf (accessed on 02/08/2014, no longer on-line).

For peer review only

- Mirzoev T, Lê G, Green A, Orgill M, Komba A, Esena R, Nyapada L, Uzochukwu B, Made W, Nxumalo N, Gilson L. 2013 Assessment of capacity for Health Policy and Systems Research and Analysis in seven African universities: results from the CHEPSAA project. *Health Policy and Planning*. 10.1093/heapol/czt065
- QAA. 2012. UK Quality Code for Higher Education (pdf). QAA: Gloucester. Available online at: http://www.qaa.ac.uk/Publications/InformationAndGuidance/Documents/B10.pdf (accessed on 16/05/2014).

Cross institution/Country wide research management

- Meek & Olson. Effectiveness of Research and Innovation Management at Policy and Institutional Levels. OECD. (undated) http://www.oecd.org/sti/Effectiveness%20of%20research%20and%20innovation%20management%20at%20policy%20and%20institutional%20levels_Meek%20and%20Olsson.pdf

The structure of the observation checklists, interview guides and the phrasing of some of the questions were based on previously designed evaluation tools, specifically those from the following references:

- Benchmarking Southern African Universities 2014 DRAFT Summary Association of Commonwealth Universities. Available online at: https://www.acu.ac.uk/focus-areas/benchmarking-african-universities (accessed on 13/08/14)
- Kirkland J, Bjarnason S, Stackhouse J. 2006 International research management: benchmarking programme . Report to HEFCE by the Association of Commonwealth Universities. Available online at: http://webarchive.nationalarchives.gov.uk/20100202100434/http://www.hefce.ac.uk/pubs/rdreports/2006//rd11_06/rd11_06.pdf (accessed on 13/08/14)
- Unpublished documents (2014) from RS-DFID Africa Capacity Building Initiative Evaluation: Toolkit (Draft)
- Unpublished documents (2013) from the Royal Society- Leverhulme African Award Evaluation: interview guides, survey methodology, findings report.
- Unpublished documents (2013) from the HRCSI Evaluation 2013: Interview guide package.
- Unpublished documents (2012) from Enhancing Laboratory Capacity Strengthening for NTD control, Deliverable One: tool for capacity gap analysis, interview guides.

For peer review only

Box 2. List of topics/items relevant to university research management and support systems (RMSS), grouped by components

**University Research Strategy**
The university has a research strategy
The research strategy is framed within the overall goals of the institution. The strategy is distinct from but links clearly with, and is complimentary to, other institutional plans, strategies and policies
The research strategy explicitly states its purpose to assist the business of the institution, identifies priorities, and monitor progress
The institution's mechanism for determining research strategy is transparent and widely owned
The institutional research strategy fully involves faculties in its design and implementation, and policies carried out by individual schools or departments are consistent with it
Implementation of the research strategy is overseen by an appropriate member of senior management. The strategy is also backed up by appropriate manpower & resources, to make sure it is implemented
The research strategy has the facility to draw on a range of evaluation mechanisms which might include sources external to the university - such as external peer review including other universities
The Research Management Office [if it exists] is fully involved in the drafting of institutional research strategies in conjunction with other appropriate offices
The research strategy is underpinned by the internal funding mechanisms for research
The research strategy is, as far as possible, responsive to the research funding environment and opportunities (at national, international and regional levels)
The research strategy seeks to add value to existing activity by proactively highlighting new opportunities for internal and external collaboration. The strategy should also promote interdisciplinary research and the development of early career researchers
The research strategy is effectively communicated, monitored, reviewed and developed/refined
Methods for evaluation of the strategy and performance indicators should be established from the outset. Key performance indicators should include a balance of quantitative and qualitative methods
The research strategy should be sufficiently flexible and defined within a reasonable time frame (e.g. 5 years) reviewed regularly, and be capable of evolving in response to events
The strategy should take into account the need for appropriate staff incentives
**Institutional Research Capacity**
The institution has a unit dedicated to research management (Research office)

For peer review only

The institution has a research committee to develop strategies to assist the University in meeting its research objectives, identify priorities, advise on stakeholder engagement, monitor research performance, discuss annual department updates, monitor national and international research policy which effect the institution<br>The research unit has an adequate number of Staff to fulfil the needs of researchers<br>Culture where research is valued, accepted, encouraged and enjoyed<br>Ethics committee exists to ensure research conducted is ethical<br>Evidence of accessible guidance to help researchers through the research process including governance and ethics<br>Clear academic honesty guidelines in place<br>Able to meet requirements of GCP and GLP<br>Sufficient facilities are available for research (studying, IT, library, technology, laboratories etc)
**Supporting funding applications**
Institutions have regular, effective and proactive means of informing academics staff on funding opportunities and the strategic directions of funding agencies where possible, these should involve direct communication between the Research Office and individual staff<br>Central research Offices have developed and strategically use key contacts in faculties schools, institutes and departments to facilitate a two way flow of information in funding opportunities and research interest<br>The institution maintains a searchable database on institutions research performance, capabilities and contact, including all past projects and proposals<br>information and current policy from all funders is maintained and communicated as appropriate<br>The Research Office holds regular information and updating sessions and targeted workshops for faculty members and graduate students with the purpose of providing information on funding opportunities, proposal development and the development of collaborative research teams to respond to one-off as well as on-going research opportunities<br>The institution seeks to establish an effective two-way communication strategy between themselves and major sponsors and proactively seek to develop that relationship<br>The institution has clear mechanisms in place to handle internal external enquiries regarding possible research and consultancy opportunities and to monitor the outcomes of these on a regular basis<br>The Research Office actively encourages collaboration between different departments within the institution including senior Academic Office, Public Relations, Marketing and Registry<br>The institution seeks to develop mechanisms to effectively track and involve alumni working in key positions with current, past and potential sponsors and in government

For peer review only

The research office actively brings key staff together in response to large scale tender and proposals proposal requests where appropriate and where consistent with research strategy<br>Institutions through the Research Office or other appropriate office, ensure that proposals are reviewed by experienced academic and research staff (externally, where appropriate) prior to submission<br>Proposals are only submitted with clear support from Head of Department of other appropriate management authority. Key personnel who need to be aware' of the project should be notified.<br>The institution approves all proposals before submission and research offices maintain records on the progress of all proposals<br>The information gained from previously submitted proposals is used to inform future proposals<br>The institution has a clear transparent and widely disseminated formula for determining the full economic cost of any give project, including indirect costs and staff time<br>full costing is calculated for each externally funded project even if this is not reflected in the price charged<br>All proposed research should be consistent with the institutions overall research strategy<br>The institutions provides clear guidance to staff and external sponsors as to which kind of projects and contractual terms are acceptable<br>The institution has clear risk assessment procedures for proposed projects which recognise the need to involve several key offices within the institution<br>The institution systematically reflects on its progress against its research strategy including regular comparisons with other institutions of similar nature
**Project Management and control**
All project proposals contain explicit statements of how the project will be managed and, where possible and appropriate, provision for the appointment of specialist staff<br>Mechanisms are in place to recognise the critical role of Principal Investigators, to ensure that they and other key actors are aware of their roles and responsibilities before commencement of the project and where required, that appropriate training is undertaken.<br>Key milestones (including reporting and financial review dates) are agreed with key actors at the outset and updated amongst all those actors throughout<br>Key actors, including Principal Investigators and Deans, are provided with regular and up to date project information (including financial, human resources, IP, and commercialization information), through on-line access or regular statements<br>Information provided to key actors, including Research Officers and Deans, pro-actively highlights any risks and obligations specific to both them and the institution.<br>Procedures are in place to ensure that all those with access to research are covered by appropriate

For peer review only

confidentiality and rights assignment agreements (depending on jurisdiction), particularly those who are covered by a contract of employment with the institution<br><br>Appropriate data management policies exist (covering ethical and legal compliance, copyright and IPR issues, data storage, security, sharing and retention)<br><br>Appropriate health and safety policies are in place (encompassing staff induction, safety officers, evacuation procedures etc)<br><br>Appropriate insurance arrangements are in place for both staff and clinical trials (if applicable)<br><br>Mechanisms are in place to ensure that intellectual property both brought to and emerging from research is identified, protected, tracked and signed off at all stages and that staff have access to specialist advice in this regard.<br><br>Procedures are in place for the appropriate monitoring of material transfer agreements.<br><br>Mechanisms are in place to identify possible delays and monitor expenditure to ensure it is in line with project budgets<br><br>The institutions has an explicit consistent framework within which academic units can predict future revenue and expenditure, especially where such income contributes to underpinning core activities<br><br>Mechanisms are in place for the disclosure and management of conflicts of interest.<br><br>Mechanisms are in place to obtain feedback project sponsors, which can be taken into account in future planning<br><br>Formal closure and continuous monitoring processes are in place ensuring that all obligations have been and continue to be met and that opportunities arising from the project are identified.
**Training and staff development for research**
Evidence of research training needs assessments<br><br>Provision of research skills training shaped around skills background and needs of different professional groups<br><br>There is availability and use of funds for research skills training for research management staff, researchers and academic staff<br><br>There is availability of a range of research skills training for students, research management staff and researchers covering-<br><br>Proposal writing, grant application, data analysis and management (including software and qualitative analysis) ethics, health and safety, GCP and GLP, generic research skills (quantitative and qualitative) academic writing and publishing<br><br>Evidence of matching novice and experienced researchers<br><br>Mentorship and supervision structures for students and early career stage researchers and new PI's

For peer review only

Individual job descriptions support research/institutional objectives<br><br>Policies are in place to support recruitment and contract negotiation for new support staff<br><br>Staff skills and abilities are matched to research needs<br><br>Seminar programmes relating to research undertaken<br><br>The research management structure and policies form a core element of induction programmes for new academic and technical staff as well as new postgraduate students.<br><br>Research strategy, policy and management issues form a core element of ongoing professional development programmes for mid-career and senior academic staff.<br><br>Staff in leadership roles (e.g. Deans) are offered appropriate instruction in research strategy, policy and management, as well as being involved in discussion of good practice within the institution<br><br>The Research Office maintains effective ongoing relationships with internal clients at all levels (faculty, department, individuals) with a view to supporting research staff and understanding their needs.<br><br>Performance measures for research management are established and are widely available/disseminated.<br><br>The institution makes provision for appropriate incentives to enhance the research activity of new and emerging researchers. Such incentives might include conference grants and other start-up funding.<br><br>Policies for providing incentives for staff research activity are transparent, easy to understand and consistent across the institution.<br><br>Career development opportunities<br><br>Career pathways exist for researchers
**Teaching capacity to support research**
Number of (half as a minimum) full-time academic staff as active and recognised contributors to subject associations, learned societies and relevant professional bodies.<br><br>Number of (third as a minimum) academic staff with recent (i.e. within the past three years) personal experience of research activity (including external examination, review panels, collaborative research)<br><br>Number of (third as a minimum)academic staff engaged in research or other forms of advanced scholarship<br><br>The outcomes of external scrutiny exercises undertaken by bodies such as the Quality Assurance Agency for Higher Education, the funding councils and professional and statutory bodies are carefully considered and actioned.
**External promotion of research**

For peer review only

Collaborations exist with external organisations (institutions, businesses, government, NGO's)
The institution is able to conform to the requirements of multiple funding agencies
Number of joint posts with other academic institutions
The institution has a clear strategy in place for all forms of intellectual property management
Clear regulations are in place to determine the ownership of intellectual property by and between staff, students and third parties. These regulations are effectively disseminated throughout the institution and externally
Academic departments and research projects are systematically monitored to identify emerging intellectual property at an early stage.
The institution establishes a register of intellectual property assets and pro-actively manages and maintains it at all stages of development and exploitation
Clear policy mechanisms are in place to govern the distribution of revenues from intellectual property between the university and other key stakeholders.
The institution's research communication strategy is consistent with the institution's overall strategy and underpins the core missions of the institution, particularly in relation to the integration of research, education and service.
There is a clear understanding of the roles and responsibilities of the different offices/officers responsible for research communication and good channels of communication exist between all these actors.
The institution pro-actively identifies projects (at various stages) and outcomes that are aligned with the university's priorities and are particularly suitable for external dissemination
The institution has a programme of events, such as launches, to profile major achievements or projects which relate to the strategic objectives and any priority research themes of the institution.
The institution has clear criteria for the type of work most likely to generate good publicity, and guidance on how to avoid poor publicity, and makes this information available to staff.
The institution has a clear strategy and procedures with regard to handling crisis communications and ensures these are disseminated to every level.
The institution seeks to make key research findings accessible to a wider audience, through the use of research summaries, expert guides and speakers lists, produced in suitable lay language and in publicly accessible formats so as to engage public understanding of the core mission of the institution (including inter-institutional partnerships).
The institution has established clear mechanisms to review and reward the performance of departments and research groups in the area of dissemination, which are integrated with an incentivisation policy providing a variety of incentives.
Mechanisms are in place for staff to report their dissemination activity. Such mechanisms maximise

For peer review only

research kudos and academic excellence and are consistent with any reporting requirements to external organisations<br><br>The institution provides assistance and systematic training programmes for staff in handling the media, and specific assistance in the drafting of press releases and publicity materials.<br><br>The institution facilitates the participation of researchers, particularly early career researchers, in international conferences and other fora to present their research findings and raise their profile<br><br>Where possible, dissemination outputs of staff are captured in a centrally managed integrated digital repository, linked to any central research activity database, which is made available to all units of the institution<br><br>The institution has a clear branding policy which is consistent with the research communication strategy.<br><br>The institution's web portal reflects the institution's core mission and strategy and is strategically and systematically managed as a key tool for promoting research to the broader community.
**National Research Uptake**
Ability of link policy to research and practice<br><br>Number of evidence based policies<br><br>Number of evidence based development interventions<br><br>Number of plans and policies to support research<br><br>Existence of national standards (accreditation, quality assurance) regarding the evaluation of research institutes<br><br>Existence of scientific councils with transparent and efficient systems in place to evaluate and disburse competitive research funds<br><br>Level of funding of research by the government<br><br>Researcher salary on par or above other countries in region

For peer review only

Box 3. Overall semi-structured interview suite of questions, by RMSS component

Biography of interviewee

What is your current position within this institution?

How long have you held this position?

How long have you worked at this institution?

What is your role in research within this institution?

1. Research strategies and policies

The institution

How many staff and students are there at this institution?

What is the percentage of income from a) teaching and b) research?

Is there core funding for research? How much? How is it disbursed?

How many PhD students are registered a) with your institution and b) externally?

Is there a university officer/directorate responsible for research? Do they have terms of reference?

How does this institution's research outputs compare to other comparable institutions? How do you measure this?

Strategies

Do you have a university research strategy?

What are the main themes/components of the strategy?

Does it link to a) national and b) other institutional strategies?

How is it disseminated internally and externally?

What are the research strengths at this institution?

Are strategies revised? How often? What were any major changes?

What are the strategic priority research areas? How were they decided? How are researchers and externally funded projects encouraged to focus on these areas?

Was any baseline information (e.g. a SWOT analysis) used to inform the strategy?

Who was involved in setting the strategy? What was the process?

For peer review only

2. Institutional Support Services and Infrastructure

Research management

Is there a university research committee? What do they do?

Is there a research support office? What do they do? (E.g. identify opportunities, help with application process, and ensure compliance with funder's requirements)

How are you made aware of research funding opportunities? (at national, international and regional levels)

How do you keep track of publications/presentations/conferences/grant applications produced/ department?

ICT (also see data management in section 4)

Is there adequate Wi-Fi, broadband speed, video conferencing and skype facilities for researchers? Do they pay for this?

Can they access the IT systems from home?

Do you purchase computers etc. on their behalf, or make recommendations? Do you set them up? Help with software? Is there any charge for this service?

How are files and information backed up? (e.g. offsite servers)

Library

How do staff and students access peer reviewed and grey literature? Are there any regular training courses offered?

How is access to e-resources and hard copy books/journals managed between the ICT unit and the library?

Laboratories

What research laboratories and field sites are available to use for research purposes at this institution?

What type of research studies can be supported by the laboratories (e.g. HPLC for pharmacokinetics; genomics/sequencing; insectory etc.)

Are the laboratories enrolled in external quality assurance systems?

Do the laboratories have international accreditation?

Do the laboratories follow Good Laboratory Practice guidelines?

Are there backup generators? Surge protection?

What sample storage facilities do you have? Are they temperature controlled and monitored?

What are the policies and processes governing transfer of samples to external institutions?

For peer review only

3. Supporting funding applications

What are the mechanisms for identifying external funding opportunities? Does the research office help with this? (E.g. is there a 'grants identification' officer?)

Is there any support to help PIs prepare funding proposals? (E.g. getting documents together, preparing/checking budgets, submitting proposals)

What is the mechanism for collating information on all proposals submitted? Is there a searchable database of submitted projects and whether they were successful?

What is the process for submitting proposals? Is there a formal sign off and if so by whom?

Do proposals have to have input or approval from finance/accountants prior to submission? What do they look for? How do you make sure that overheads are included and the costings are correct (e.g. salaries, equipment)?

Does the university use external advice (e.g. legal) at any stage during the process?

Do you have any way of comparing your research performance with other institutions?

4. Project management and control

What systems are in place to monitor the progress of each project? (E.g. against milestones)

Ethics

How is this managed in the university as a whole? Is this done at the university or at the faculty level?

Is ethics committee membership GCP-compliant?

Are there guidelines about how the ethics committee functions?

Are there guidelines for researchers about the ethics process?

Are there guidelines relating to academic honesty and plagiarism?

Financial

Who provides financial reports to funders? Who has specialist knowledge of each funders' reporting requirements?

How often are financial reports made to PI's (frequency, method, feedback loop?)

How do departments predict and plan future research revenue and expenditure?

How does the university ensure that project expenditure remains in line with the budget?

Legal

For peer review only

What is the process for minimising risks regarding financial and contractual terms? Is legal advice available? Who accesses this and when? (ie during the contract signing process or only if there is a problem)?

How are appropriate insurance arrangements organised (particularly for field staff and clinical trials)

What regulations are in place to determine the ownership of intellectual property by and between staff, students and third parties? How are these regulations disseminated throughout the institution and externally?

If what ways do you identify emerging intellectual property in your academic departments and on-going research projects?

Have you established a register of intellectual property assets? How are these managed and maintained?

What policies/mechanisms are in place to govern the distribution of revenues from intellectual property between the university and other key stakeholders?

Data management

Are there research data management guidelines and/or policies for data protection and storage?

How is research data backed up and secured? How are routine office and research documents (e.g. draft publications, guidelines/protocols etc.) backed up and secured?

Who is responsible for these systems? Are PIs charged for this service?

Do you provide help for PIs to complete Data Management plans to funders?

What are the mechanisms for managing data ownership, data security, licensing for re-use, data sharing, reuse of third-party data, restriction of data sharing (prior to publishing or seeking patents, retaining/destroying data?

Clinical work/trials questions:

Does the university acts as a sponsor for clinical trials?

Is there a clinical trials office? What does it do?

How do you do clinical monitoring? Have any audits about this been conducted and if so what were the key findings?

5. Human Resource Management for Research

Are job descriptions available for researchers and support staff?

Is there an induction process for new employees?

What are the processes for promotion for a) researchers and b) support staff (e.g. administrators, laboratory scientists)?

For peer review only

What mentorship and supervision structures exist for students and early career stage researchers and new PI's?

What career pathways are there are for a) researchers and b) research support staff?

Is there any ongoing professional development programme and does this cover research skills?

Do you know about the MCDC career development groups (CDGs)?

How do the MCDC career development groups (CDGs) fit into/complement institutional systems?

Are these career development activities embedded in institutional structures?

Do you think they are helpful? Should they be institutionalised? Why/why not?

Human resources

What Policies/strategies are in place for Human resource development of a) researchers/scientific staff, b) admin staff (including training, retention, tenure track, funding)

Do you have a formal induction process for new employees? Is there a special one for researchers?

Are there health and safety policies? (E.g. staff induction, safety officers, evacuation procedures etc.)

How are training needs identified? (E.g. staff training needs assessments). Is HR responsible for providing and/or recording any research training (e.g. GCP/GLP training, proposal writing, project management, supervision)?

How are training opportunities identified and funded? Is there a core budget for training and how is it allocated?

What proportion of research posts are a) core funded and b) project funded?

Are you involved in all new appointments? Do you advise PIs on the institution's procedures governing the employment of staff?

Is career guidance given to PhD students, post-docs and other researchers?

How are post-docs absorbed into the workforce?

What is the process and turnaround time for recruiting and appointing new research staff?

Do you make more internal or external research appointments?

Does your institution offer the possibility of short-term bridging funding to retain research staff during hiatus periods between grants?

Can you describe what Performance measures are used for research management and how these are reported?

Are there joint posts with other academic institutions? How do they work and are they effective?

For peer review only

6. Human Resource Development for Research

Is training available on

- Research design (epidemiology, stats, social science, health systems)
- Ethics, health and safety, GCP and GLP
- Data analysis and management (including software and qualitative analysis)
- Academic writing and publishing
- Proposal writing, grant application
- Teaching and education
- Leadership and management

Are there facilities and fora (e.g. seminars, journal club, staff exchanges) for researchers to discuss their work regularly with each other?

Is a tracking system in place for PhD students? How many supervisors do PhD students have? How many students do PhD supervisors have?

Are there minimum standards in place about the level of supervision to be given?

7. External promotion of research

Do you have longstanding research collaborations with external organisations? Examples?

Do you have a research communication strategy? A research communication unit?

Who is responsible for research communication? Do researchers perceive that this is their responsibility?

Does the institution have a research profile on its website?

How do you make key research findings accessible to a non-academic audience (e.g. research summaries in lay language and in publicly accessible formats)?

Do you have a programme of events, such as launches, to profile major achievements or projects

Do you provide advice to staff about how to deal with the media (e.g. how to generate good publicity and avoid poor publicity)?

What strategy and procedures are communicated to staff with regard to handling crisis communications and how are these disseminated?

Are there incentives for departments and research groups in the area of dissemination?

8. National research engagement

What level of funding for research is provided by the government?

For peer review only

Are there national policies and plans regarding health (and other) research?

How does the institution engage with policy makers?

Are there national scientific councils that evaluate and disburse competitive research funds? Do they have transparent and efficient systems?

What are the mechanisms by which research from your institution influences policy and practice?

Are there national standards (accreditation, quality assurance) regarding the evaluation of research institutes?

For peer review only

BMJ Open

A mixed methods study to develop a process and tools for the assessment and tracking of African institutions' capacity for operational health research

Journal:	*BMJ Open*
Manuscript ID	bmjopen-2017-016660.R1
Article Type:	Research
Date Submitted by the Author:	21-Apr-2017
Complete List of Authors:	Wallis, Selina; Liverpool school of tropical medicine, International health<br>Cole, Donald; University of Toronto, Dalla Lana School of Public Health<br>gaye, oumar<br>mmbaga, blandina<br>mwapasa, victor<br>tagbor, harry<br>bates, imelda
**Primary Subject Heading**:	Global health
Secondary Subject Heading:	Global health
Keywords:	Research activities, Institutional practices, Systems analysis, Organization & Administration, Evaluation

SCHOLARONE™
Manuscripts

For peer review only

A mixed methods study to develop a process and tools for the assessment and tracking of African institutions' capacity for operational health research

Selina Wallis, Donald C Cole, Oumar Gaye, Blandina T. Mmbaga, Victor Mwapasa, Harry Tagbor, Imelda Bates

Corresponding author's name, address, telephone/fax numbers and e-mail address;

Imelda Bates, Liverpool School of Tropical Medicine, Pembroke Place, Liverpool L3 5QA

Tel: 0151 705 3115; Imelda.bates@lstmed.ac.uk

Keywords [MeSH] Research activities, Institutional practices, Systems analysis, Organization & Administration, Evaluation

Abbreviated Running Title - Assessing research management systems

Abstract

Objectives: Research is key to achieving global development goals. Our objectives were to develop and test an evidence-informed process for assessing African universities' health research management systems (RMSS) and for tracking interventions to address capacity gaps.

Setting: Four African universities

Participants: Eighty-three university staff and students from eleven cadres

Intervention/methods: A literature-informed 'benchmark' was developed and used to itemise all components of a university's health RMSS. Data on all components were collected during site visits to four African universities using interview guides, document reviews and facilities observation guides. Gaps in RMSS capacity were identified against the benchmark and institutional action plans developed to remedy gaps. Progress against indicators was tracked over 15 months and common challenges and successes identified.

Results: Common gaps in operational health research capacity included: no accessible research strategy, a lack of research e-tracking capability, and inadequate quality checks for proposal submissions and contracts. Feedback indicated that the capacity assessment was comprehensive and generated practical actions, several of which were no-cost. Regular follow-up helped to maintain focus on activities to strengthen health research capacity in the face of challenges.

Conclusions: Identification of each institutions' strengths and weaknesses against an evidence-informed benchmark enabled them to identify gaps in in their operational health research systems, to develop prioritised action plans, to justify resource requests to fulfil the plans, and to track progress in strengthening RMSS. Use of a standard benchmark, approach and tools enabled comparisons across institutions which has accelerated production of evidence about the science of research capacity strengthening. The tools could be used by institutions seeking to understand their strengths and to address gaps in research capacity. Research capacity gaps that were common to several institutions could be a 'smart' investment for governments and health research funders.

For peer review only - http://bmjopen.bmj.com/site/about/guidelines.xhtml

Trial registration: none

Strengths and limitations of this study

- This study uses mixed methods research to generate primary, prospective, longitudinal data about the baseline status of operational health research systems in four African institutions, and tracks changes in research capacity against pre-determined indicators
- The use of the same benchmark and research approach across different institutions enables comparisons to be made so common challenges can be identified; these could be effective targets for investment
- The main limitations for the study were that the limited follow up time did not allow for demonstration of the long-term sustainability of changes to research systems and, because our study was designed to provide a broad overview of an institution's RMSS, it did not explore particular components in depth
- Institutions found the evaluation process to be comprehensive and helpful since in addition to advancing the science of research capacity strengthening it generated practical actions and progress indicators, and facilitated inter-institutional comparison and bench-marking

Source of funding: This work was supported by a grant from the Wellcome Trust, UK, to the London School of Hygiene and Tropical Medicine MCDC project (http://www.mcdconsortium.org/phd-programme.php). The funders had no role in study design, data collection and analysis, decision to publish, or preparation of the manuscript.

Competing interests
We have read and understood BMJ policy on declaration of interests (http://www.bmj.com/about-bmj/resources-authors/forms-policies-and-checklists/declaration-competing-interests) and declare that we have no conflicts of interest

Word count: 4602 excluding authors' contribution statement, tables, figures/diagrams and references

For peer review only - http://bmjopen.bmj.com/site/about/guidelines.xhtml

Process and tools for the assessment and tracking of African institutions' capacity for operational **health research**

INTRODUCTION

Importance of research for development

Health research has been acknowledged to play a key role in progress towards the Sustainable Development Goals.[1] Strong research institutions and skilled researchers are essential for low- and middle-income countries (LMICs) to generate evidence for their own health policies and to make progress in achieving their health related goals.[2, 3] Investments in operational health research capacity, can provide positive returns by promoting evidence-informed policy and practice in the health system,[2] although implementation[4] and estimation of returns can be challenging.[5] The first African Ministerial Conference on Science and Technology in 2003 recognised that "There is strong evidence that using research evidence to inform policy and practice leads to benefits which contribute to socioeconomic development"[6] and participating countries committed to spend at least 1% of their GDP on research and development by 2010.[7] Only Kenya, Malawi and South Africa have managed to approach this target and Kenya, Mozambique, Senegal and Uganda all have more than 40% of their research and development financed from abroad.[7]

Lack of research/researchers in LMICs especially Africa

Although the average growth rate of scientific production in Africa is faster than that of the world as a whole, African Union countries only produce 2% of the world's total scientific output.[8] Egypt, Kenya, Nigeria, and South Africa produce the largest number of publications from Africa.[7] This is a reflection of the small numbers of researchers in Africa and decades of under-investment in research institutions. Most countries in sub-Saharan Africa (SSA) have less than 500 researchers (of all disciplines) per million inhabitants (e.g. Tanzania 35, Ghana 39, Malawi 50, Senegal 361) compared to over 4000 per million inhabitants in the UK and North America.[9] There are numerous disincentives to pursuing a research career in many African countries including heavy teaching loads, weak organisational research systems, lack of national research leadership, limited access to scientific information, slow internet connections and inadequate physical facilities including libraries and laboratories.[10]

Attempts to address weak capacity for operational health research in Africa

Resources to guide development of operational health research capacity have been available for at least a decade[11] but outdated and ineffective models for strengthening capacity persist.[12] African research institutions have historically faced numerous challenges.[13] The ability to produce international quality health research depends not only on developing a critical mass of African researchers, but also on providing them with a conducive environment in which to do research and progress their careers.[14, 15] International funders have responded by supporting strengthening of national systems and structures for operational health research[16] and in boosting the capacity of low- and middle-income country universities in research governance and management.[17] However, despite longstanding calls for more robust evaluations of capacity development,[11] the evidence needed to inform effective implementation and evaluation of programmes for strengthening operational health research capacity remains weak.[18, 19] Furthermore, the lack of clearly defined goals and baselines against which to evaluate the success of research capacity strengthening programmes makes it difficult to track their progress and impact.[20]

For peer review only - http://bmjopen.bmj.com/site/about/guidelines.xhtml

Development funders and policy makers are calling for a “significant re-think of the approach to capacity development”.[21] They stress the need for an inter-disciplinary approach which recognises the complexity, fluidity and non-linearity in human systems, a systematic perspective, and acknowledgement of relationships between capacity at the individual, institutional and wider societal levels.[22, 19] To promote a more purposeful and strategic approach to strengthening operational health research capacity in LMICs, a group of international funders have produced guidance about developing shared principles and indicators,[23] and for evaluating outcomes and impacts of health research capacity strengthening interventions. Putting these guidelines into practice at the organizational level is challenging since little is known about what information matters for strengthening research capacity, and how and why this varies in different institutional contexts.

Purpose

The purpose of our study was to develop and test an evidence-informed process that could be used 1) to conduct a baseline assessment of operational health research management and support systems (RMSS) in African universities and 2) to document actions taken to address identified gaps. As institutions implemented these actions, we sought to identify common difficulties they encountered. This information would help not only the institutions, but also external agencies and national governments, to more effectively target and monitor their contributions to strengthening institutional and hence, national health research capacity. The assessment process covered all the components needed for a university to generate, manage and disseminate operational health research of international quality.

Approach to the study

The study comprised three phases – construction of a benchmark against which to conduct the baseline assessments of institutions’ RMSS, development of data collection tools based on the benchmark and collection and analysis of data during visits to the institutions and the follow up period. Despite earlier work on research management benchmarking,[24] no single document existed which detailed all the systems needed in a university to foster, support and manage international quality operational health research. Hence it was necessary to develop a comprehensive description of the components of an ‘optimal’ scenario[19] as a benchmark against which the baseline assessment could be compared.[22, 25] We describe the process of using best available evidence to generate this benchmark as a health Research Management and Support Systems (RMSS) list and used the benchmark to craft tools for collecting baseline data in each of the universities and to collate a list of indicators for monitoring progress. We share our experience of using the tools to identify institutions’ RMSS capacity gaps, the early results on tracking the universities’ progress and challenges in strengthening their RMSS and senior researchers’ experience with the RMSS assessment process.

METHODS

Partner universities

We worked with four African universities which were partners in the Malaria Capacity Development Consortium (MCDC 2008-2015, http://www.mcdconsortium.org/) funded by the Bill and Melinda Gates Foundation and the Wellcome Trust. MCDC supported African scientists to undertake high-quality malaria research and to enhance the operational health research capacity of their home institutions. In particular, MCDC aimed to strengthen the capacity of the African universities to provide academic, administrative and financial support to generate health research of international quality despite differences in geography, size and maturity of their research infrastructure.

For peer review only - http://bmjopen.bmj.com/site/about/guidelines.xhtml

The universities were based in Anglophone and Francophone countries in West (2), East (1) and Southern (1) Africa. The entry point for our study into each of the universities was the department (or centre) in which MCDC's collaborating principal investigator was located. These departments had been established between 1957 and 1991; all had active malaria research programmes and offered postgraduate training. The departments' universities were located in west (Anglophone and Francophone), east and southern Africa and at the time of the study had between 6,000 and 60,000 registered students.

Generation of a list of research management and support system components

In order to conduct a holistic assessment of the African universities' health RMSS it was necessary to first create a benchmark by identifying all the components and related best practice required for the optimal functioning of such systems.[19] As no single document available detailed all these components, we drafted an initial list of components by itemising all activities that occur within a project cycle and by identifying all the support mechanisms that are required to conceive, generate and monitor research and to ensure that research findings are used to inform national health policies and practices. The list identified search terms (e.g. research management, research capacity indicators, institutional benchmarking) which guided the collection of relevant information using internet searches. The search for relevant global publications included academic articles, and grey literature such as guidelines and regulations governing research aspects of higher education institutions (Supplementary file Box 1). We also interrogated websites of agencies relevant for each of the themes, and read their reports and documents and any references included therein and consulted with researchers, grants managers and research finance officers within and beyond our own institutions until no new items emerged and saturation was achieved. We aimed to cover aspects of the institutional capacity needed to provide optimal academic, administrative and financial support for operational health research activities from the perspectives of the Dean or Principal of the institution, faculty research support staff and researchers at different career stages.

From the literature (examples in Supplementary file Box 1) we extracted a list of all the items relevant for inclusion in a review of institutional RMSS. To help the development of systematic data collections tools, items on the list were grouped into components which were simultaneously adjusted and expanded to encompass all the aspects of RMSS, with no duplication across components (Supplementary file Box 2). The 'optimal' scenario for an institutional RMSS was therefore derived by amalgamating all the items identified from the literature search and eliminating any redundancy. In order to ensure comprehensiveness and minimise bias, no assumptions were made about what should be included, no selection criteria were applied to the original list of items and they were drawn together under the eight components without losing any of the items. This list of items therefore represented the description of the 'optimal' scenario (i.e. benchmark). The final RMSS components encompassed all the RMSS-relevant items identified in the literature and were:

1. Research Strategies and Policies
2. Institutional Support Services and Infrastructure
3. Supporting Funding Applications
4. Project Management and Control
5. Human Resource Management for Research
6. Human Resource Development for Research
7. External Promotion of Research
8. National Research Engagement

For peer review only - http://bmjopen.bmj.com/site/about/guidelines.xhtml

Development of tools for data collection

The most appropriate methods to be used for collecting data on each of the components and their associated items during subsequent visits to the universities were determined.[26] The primary data collection tool was a guide for semi-structured interviews with different cadres of university staff, supplemented by a list of facilities to be visited at the institutions (i.e. library, IT suite, laboratories) and a list of documents to be reviewed (i.e. strategies, policies, regulations, handbooks).

Inclusion of the entire master list of items for every component in every semi-structured interview would have been impractical and inappropriate. Since each interviewee would have knowledge of specific aspects of RMSS in their institution, combinations of questions were selected from an overall suite (Supplementary file Box 3) to construct focused interview guides for different cadres of interviewees (i.e. Heads of Department/Institute Deans or Principals; senior researchers; staff with research support responsibilities such as administration, finance, human resources, communications, ethics and laboratories). For example, questions for laboratory technicians, but not for other cadres, dealt with equipment maintenance. We ensured that all items from the master list were covered across the set of cadre-specific interview guides.

The data collection tools (lists and interview guides) were reviewed by all members of the research team and adjustments were made to reduce redundancy. Additional changes were made after the first university visit and minor revisions were made during the visit to the second university. After this, no more revisions were required, so this version was used for the two subsequent visits.

Baseline data collection during university visits

Pre-visit briefings were conducted by Skype with the MCDC principal investigator in each of the African universities, to explain the purpose and process of the visits and to schedule interviews. They were provided with the data collection tools in advance of the visits so they were aware of the range and type of information that would be sought. Subsequently, 3-4 day visits to each of the four African universities were conducted by 2-3 members of the research team between September and November 2014.

As far as possible, all data collected during the visits was obtained from at least two independent sources to enhance validity.[27] Interviewees were asked if any aspects of research systems had not been covered by the interview questions and, as a result, procurement procedures were added to the questions for the second and subsequent visits. During each interview, interviewees were asked to propose feasible actions that could be taken to overcome any of the challenges or gaps in research support systems that they mentioned.

Notes from the interviews were typed up within a few hours of each interview, checked against audio-recordings of the interviews (available if interviewees gave permission), and final versions were verified among the site visit team. Information from observation of facilities and review of documents was used to elaborate and verify data from the interviews. A consultation meeting was held at the end of each visit for all available interviewees to share preliminary findings about strengths and gaps identified in the institutional RMSS. In keeping with the principles of inter-disciplinary team reflexivity[28] and of pooling internal and external assessments,[29] we used the meetings to check the accuracy of the findings, to discuss the reasons for discrepancies, to generate and prioritise proposed actions, and to ensure that such actions were deemed feasible by institution staff.

For peer review only - http://bmjopen.bmj.com/site/about/guidelines.xhtml

Baseline Data analysis

A framework analysis approach was used to manage and analyse the multi-disciplinary information generated from the site visits about institutions' 'baseline' research systems.[30] Data were entered into a matrix which had a row for each of the eight components. Columns for topics within each of the RMSS components that emerged from the interviews were constructed using deductive (i.e. based on the topics/items grouped under each component from the scoping review) and inductive (i.e. unexpected new topics that emerged from the information collected) approaches. Use of the matrix facilitated identification of emerging patterns and comparison of the strengths and weaknesses in each institution's research systems. Following the site visits, findings were presented in a draft report which was reviewed by the MCDC principal investigators in consultation with their institutional colleagues, before being finalised. To respect confidentiality, the final reports were only shared with the MCDC secretariat and the institutions themselves. An anonymised 'overview' report was produced and made publically available which summarised commonalities and differences in RMSS across all institutions and highlighted innovative RMSS practices.[31]

Follow-up interviews for tracking progress and obtaining feedback on the process

Information about progress and challenges in addressing gaps in the institutions' health RMSS was obtained through 2-5 Skype and telephone interviews with the MCDC principal investigators in each institution over fifteen months until May 2016. Each interview lasted 20-40 minutes and covered the gaps and actions identified in the relevant intuitional baseline report. The relevant principal investigator, in discussion with SW and IB, gauged the progress on each action, explained the means by which progress had been achieved, and described any challenges experienced.. During the interviews the principal investigators were asked to comment on whether the process had been helpful, and if so how, and which aspects could be improved in the future and to reflect on their role as research manager practitioners.[32] These comments were organized into themes, and quotes reflective of each theme were selected to convey the principal investigators' perspectives in their own words.

Information obtained about progress and challenges around actions in the baseline report were mapped against the eight RMSS components using a pre-prepared matrix and analysed using a framework analysis approach. Two authors (SW, IB) reviewed the self-reported progress of each institution and broadly assessed whether the institutions collectively had made 'good', 'moderate' or 'little/no' progress in addressing the gaps in each component of their research support systems. This helped understanding about which components of research support systems all four universities found most easy to address and which they found hardest. A report outlining progress and challenges was drafted for each institution and reviewed by each principal investigator.

Ethical considerations

This project was considered to be primarily an evaluation which aimed to improve practices for strengthening research capacity so formal ethical approval was not sought. However we explained the study to all participants, we asked each interviewee for their verbal consent to participate and we provided an opportunity for them to refuse without any consequences for themselves.

For peer review only - http://bmjopen.bmj.com/site/about/guidelines.xhtml

RESULTS

Baseline situation

In total 83 interviews were conducted (19-22/university) with eleven different cadres of interviewees (table 1), 65 documents/resources (12-20/university) were reviewed, and facilities observed included libraries, research laboratories and study spaces. The gaps in RMSS that were common (i.e. occurred in at least three of the four universities), and proposed actions that emerged during the on-site visits to address these gaps, were categorised by RMSS component (table 2).

Progress in strengthening universities' RMSS

All of the universities had made some progress in addressing gaps in their research support systems, and there were some common successes and challenges. Examples are provided in(table 3. Although MCDC provided some institutions with limited funding to address some of these gaps, many of the actions, such as re-organisation of management structures or in-house training, did not require additional funds.

Overall, little or no progress was made in Research Policies and Strategies, External Promotion of Research, and National Research Engagement; moderate progress was made in Institutional Support Services and Infrastructure and Human Resource Management and Development for Research; and good progress in Funding Applications and Project Management and Control. Examples of innovative practices and problem-solving were identified for each component (table 3).

The process of assessing and tracking strengthening of RMSS

The process of assessing and providing feedback on institutional RMSS used in the study was universally viewed as a positive and constructive way to raise awareness of the importance of strengthening research support systems and to catalyse broader institutional engagement with these topics. Relevant comments from interviews with the principal investigators included:

"Senior staff are really engaging with this. They understand the importance of the programme"

"The project definitely helped to raise awareness of all the challenges we are facing, that we need more funds and to improve the environment; it highlighted difficulties and that all the partners are now really interested in helping African institutions. It enabled us to start some concrete actions and now we have institutional buy in, now they are engaged and committed to go further"

An area for improvement was in ensuring that important documents provided to institutions, such as drafts of the research capacity assessments, were produced in French as well as English language.

"It would help if the report was in French, with logos, stamp and signature - an official version. Otherwise a translation is not taken seriously"

The comprehensive nature of the assessments and data collection tools provided confidence that all key aspects of research support systems had been covered during the process and helped stakeholders to prioritise and justify their future budgeting and funding requests.

"It was very useful to get an overview of the whole system from an outside team"

"A piecemeal approach would not be effective at all. We need to look at each area. We can then leverage funding ….and use this [assessment] to make sure every area is funded."

For peer review only - http://bmjopen.bmj.com/site/about/guidelines.xhtml

For peer review only

The collaboration between an external team and stakeholders within the institutions brought additional benefits in terms of impartiality and reduction in bias, which would not have been possible with an exclusively internal review team. Seeking opinions from multiple perspectives and the involvement of external team, helped to overcome internal sensitivities.

"It stimulated honest and fair discussion between us all…… It demonstrated our strengths as well as weaknesses. Everyone said it didn't say anything we didn't know but as an outside organisation produced it there were no biases. That's why everyone has agreed we need to move forward"

"Certain areas that the overall report helped when I was presenting the sensitive issues that there are common problems - instead of feeling hopeless, we felt we were doing better in some areas. We knew … that here are political issues. If the recommendation had come from within that could have caused issues"

Addressing gaps in research support systems is a complex undertaking and regular contact with the external team to track progress was helpful for keeping the focus on priorities and maintaining momentum.

"The follow up process was helpful to keep me focused on understanding the changes occurring across the college and in all areas of research management."

"On-going follow up was helpful to keep on track with forward movement"

DISCUSSION

Process and Tools

We have demonstrated that is it possible to construct and implement a coherent, evidence–informed process for assessing and tracking programmes to strengthen institutions' health RMSS. The comprehensive data collection tools drew on current approaches and evidence from several disciplines including research management, education and organisational systems.[33, 34, 35] It has parallels with others' efforts[36] to construct assessment tools to improve the quality of indicators and processes for measuring operational health research capacity strengthening.[20] The assessment process was systematic yet flexible enough to accommodate the complexity and fluidity of health RMSS, across a range of African universities. The assessment process acknowledged the influence of inter-relationships between individual, institutional and wider societal levels on the 'research ecosystem' (i.e. researchers and their institutions, funders and governments who support research, policymakers who use research, and communication specialists who share and discuss the findings with a broad audience).[37] The way in which the assessment process was conducted, particularly the findings from the baseline assessments and the collaborative identification of actions to address health RMSS gaps, was universally viewed as positive, and is consistent with others' experience in reviewing operational health research capacity.[36, 4] In addition the institutional assessments helped to raise awareness of the importance of strengthening RMSS[18] and to catalyse multi-disciplinary engagement in improving RMSS across the institutions.[38]

Such assessments would be difficult for exclusively internal teams to undertake since they may struggle to gain timely access to senior university officials and could be influenced by sensitivities and politics within the institutions. A partnership between senior institutional researchers, who intimately understood the structural, financial and political context, and an external team, who were impartial and experienced in such assessments, was therefore essential to maximise assessment

For peer review only - http://bmjopen.bmj.com/site/about/guidelines.xhtml

validity and contribution to learning.[18] Such insider-outsider assessments have also been used in examining research ethics systems.[29] The transferability of the RMSS assessment tools and processes across geo -political and institutional boundaries means they could be usefully deployed in the increasingly common model of research consortia.[24] Of note is the need to produce reports for non-Anglophone universities in the country's dominant language, since language barriers are known to be a critical handicap in scientific collaborations and in engaging senior university officials.[39]

Tracking Progress/Challenges

Although there are numerous publications of retrospective evaluations of research capacity strengthening efforts, prospective tracking of progress is far less common.[40] We applied an established five step process for assessing baseline status and prospectively tracking changes in operational health research capacity.[18] The researchers perceived the process as constructive since it helped to maintain focus and momentum within the institution, and provided an opportunity to introduce and share innovative approaches to problem-solving at each institution and for each RMSS component. Most institutions had made the best progress in areas that were primarily under the control of the collaborating senior researchers' departments, such as involving finance officers and managers in developing research proposals, and providing training and resources for managing grants. Much of this progress was achieved with limited or no additional funds. This may therefore be a useful indicator of what might be achieved by other research institutions in Africa who have minimal external support.

Gaps in operational health research capacity that were generally found to be most the challenging to remedy depended on university-wide changes. Examples included embedding research training, which was usually non-sustainably linked to projects, within university systems, and ensuring laboratories were accredited and underpinned by sustainable financing models. Most challenging of all were the lack of systems for communication and dissemination of research outputs and for using research to influence health policies and programmes. This lack of institutional knowledge exchange capacity to promote research uptake in Africa has been noted by others.[41]

Limitations of the study

Our study was designed to provide a broad overview of an institution's health RMSS, and therefore could did not explore particular components in depth. Other instruments and guidelines are available to do this including: Good Financial Grants Practice,[42] for researchers' development framework,[34] Octagon for research ethics capacity,[29] 'stepwise' laboratory accreditation,[43] and DRUSSA for research uptake.[44] The MCDC principal investigators varied in their seniority, influence and social capital[45] (i.e. the norms and networks that enable people to act collectively) which may have affected the thoroughness of the assessment phase, as well as the extent of progress especially in implementing university-wide actions. We recognise that the study only included four African institutions and that these cannot be considered representative of the diversity and complexity of universities within the continent and even within individual countries. The lack of a theory of change[46] for the broader MCDC programme meant that explicit articulation of a common set of outcomes and pathway to change for strengthening RMSS was lacking.[47] Tracking information in progress was generally not independently verified, as it was based on Skype or phone interviews with the MCDC principal investigators. The follow up time was 15 months which is too short to be able to demonstrate longer term impact of such a process on health RMSS. Hence, we regard our prospective tracking as an initial experience which could be used to guide a more fulsome, prospective evaluation.

For peer review only - http://bmjopen.bmj.com/site/about/guidelines.xhtml

Contributions to an emerging science

Momentum is gathering around a new global science on research capacity strengthening which draws on implementation research,[48] research evaluation processes[5] and mixed methods research methodologies.[49] Our effort is consonant with this developing global science, addressing the area of health RMSS with an explicit and comprehensive set of assessment tools, embedded in a collegial, collaborative process. Similar to a small but growing number of colleagues engaged in contributing to the science-base for research capacity strengthening, we are sharing our tools in a peer-review forum, so that others can apply and adapt them for assessing their own or others' university's RMSS. Linking collaborative RMSS assessments of gaps with collegial generation of actions to address those gaps, and jointly tracking progress on chosen actions and challenges prospectively constitutes a more rigorous approach to operational health research capacity strengthening than has been common to date.[20] In addition, documentation of innovative problem-solving by African institutions is crucial to counter deficit-focused narratives, facilitate sharing among resource-constrained institutions, and facilitate universities' role as agents of change.[50] An additional benefit of using a systematic, common approach to strengthening institutional health research capacity is that it provides evidence for external agencies and governments about better targeting of efforts to make institutions in Africa globally competitive research leaders.

IMPLICATIONS

Research capacity outputs need to be recognised as of equivalent value to research outputs[12] and therefore need a rigorous scientific basis. Our experience in developing and applying an assessment and tracking framework can facilitate similar initiatives in other research oriented institutions in LMICs and their respective consortia. The identification and sharing of RMSS components that are commonly problematic could guide national governments to target their resources towards these weakest components. At the supra-national level, the use of our tools and process, and sharing of the results more widely, enable comparisons to be made across institutions and countries. Such analyses would not only contribute to the science of operational health research capacity strengthening, by enabling common research approaches and tools to be applied in different contexts and by validating findings on common capacity gaps, but also provide guidance to international health and research funders about 'smart' investment of resources. Sharing of problem-solving innovations in RMSS among universities and research institutes with similar resource constraints through such organizations as the African Academy of Sciences is an important more immediate opportunity. Finding ways to share such innovations widely beyond health, for example through inter-disciplinary study tours or joint workshops for researchers and research support staff, is imperative for fostering collaborations for RMSS strengthening, and hence health system strengthening more broadly.

Contributorship statement

SW: Liverpool School of Tropical Medicine, Liverpool, UK (mixed methods researcher) contributed to literature review, study design, baseline and follow up data collection, analysis and final paper

DCC: Professor, Dalla Lana School of Public Health, University of Toronto, Canada (mixed methods researcher)

For peer review only - http://bmjopen.bmj.com/site/about/guidelines.xhtml

contributed to literature review, study design, baseline and follow up data collection, analysis and final paper

OG: Professor, Cheikh Anta Diop University, Dakar, Senegal (MCDC Principal Investigator) contributed to providing baseline and follow up data and final paper

BTM: Kilimanjaro Clinical Research institute, Kilimanjaro Christian Medical Centre and Kilimanjaro Christian Medical University College Moshi, Tanzania (MCDC Principal Investigator) contributed to providing baseline and follow up data and final paper

VM: College of Medicine, University of Malawi, Blantyre, Malawi (MCDC Principal Investigator) contributed to providing baseline and follow up data and final paper

HT: Kwame Nkrumah University of Science and Technology, Kumasi, Ghana (MCDC Principal Investigator)
contributed to providing baseline and follow up data and final paper

IB: Liverpool School of Tropical Medicine, Liverpool, UK (mixed methods researcher)
led study design, contributed to literature review, baseline and follow up data collection, analysis and led drafts of final paper

Acknowledgements
We acknowledge the collaboration of staff of participating universities, which have remained anonymous for reasons of confidentiality of the findings. Thanks to other members of the study and administration team, Ema Kelly and Vicki Doyle of Capacity Development International, Dorte Holler Johansen from the University of Copenhagen, Denmark, and Lorelei Silvester and Denise Wellings from LSTM for their valuable contributions to this study

Data sharing statement
No additional data are available
All data related to this study are included in this submission, either in tables in the manuscript or in supplementary files

For peer review only - http://bmjopen.bmj.com/site/about/guidelines.xhtml

References

1. World Health Organization. Health in 2015: from MDGs, Millennium Development Goals to SDGs. Sustainable Development Goals. Switzerland: World Health Organization. Available: http://apps.who.int/iris/bitstream/10665/200009/1/9789241565110_eng.pdf?ua=1 (accessed 27 February 2017)
2. Koon A, Nambiar D, Rao D. 2012. Embedding of research into decision-making processes. Geneva: Alliance for Health Policy and Systems Research, WHO
3. Bennet S, Akua Agyepong I, Sheikh K, Hanson K, Ssengooba F, Gilson L. 2011. Building the field of health policy and systems research: an agenda for action. *PLoS Medicine* 8: 1023
4. Hawkes S, Aulakh BK, Jadeja N, Jimenez M, Buse K, Anwar I, Barge S, Odubanjo MO, Shukla A, Ghaffar A. 2016. Strengthening capacity to apply health research evidence in policy making: experience from four countries. *Health Policy and Planning* 31(2): 161-70
5. Neufeld V, Cole DC, Boyd A, Njelesani D, Bates I, Hanney SR. 2014. Perspectives on Evaluating Global Health Research for Development: A Background Paper. Taking stock of current practice and ways to improve it. For GHRI-IDRC. 79 pp. p9-11. www.ccghr.ca/wp-content/uploads/2014/07/EGHR4D-final-paper.pdf (accessed 27 February 2017)
6. Newman K. 2014. What is the evidence on the impact of research on international development. London: DFID. https://assets.publishing.service.gov.uk/media/57a089aced915d622c000343/impact-of-research-on-international-development.pdf (accessed 27 February 2017)
7. NEPAD Planning and Coordinating Agency (NPCA) 2014. African Innovation Outlook 2014, NPCA, Pretoria. http://www.un.org/africarenewal/sites/www.un.org.africarenewal/files/AIO_2_Final%20Product[2].pdf (accessed 27 February 2017)
8. Neupane B. 2015. A more developmental approach to science. UNESCO science report: towards 2030 http://en.unesco.org/unesco_science_report (accessed 27 February 2017)
9. United Nations Educational, Scientific and Cultural Organisation (UNESCO) Institute for Statistics. Number of Researchers per million inhabitants by country. <http://www.uis.unesco.org/ev.php?URL_ID=3755&URL_DO=DO_TOPIC&URL_SECTION=201>. converted by ChartsBin statistics collector team 2011, Number of Researchers per million inhabitants by Country, ChartsBin.com, <http://chartsbin.com/view/1124>.
10. Vogel I. 2011. Research Capacity Strengthening: Learning from Experience. UKCDS, London. http://www.ukcds.org.uk/sites/default/files/content/resources/UKCDS_Capacity_Building_Report_July_2012.pdf (accessed 27 February 2017)
11. Nuyens Y. 2007. 10 best resources for health research capacity strengthening. *Health Policy and Planning* 22(4): 274-276
12. Franzen SRP, Chandler C and Lang T. Health research capacity development in low- and middle-income countries: reality or rhetoric? A systematic meta-narrative review of the qualitative literature. *BMJ Open* 2017;7:e012332
13. Sawyerr A. 2004 African universities and the challenge of research capacity development. *JHEA/RESA* 2(1): 213–42.
14. Miiro GM, Oukem-Boyer OOM, Sarr O, Rahmani M, Ntoumi F, Dheda K, Pym A, Mboup S, Kaleebu P. 2013. EDCTP regional networks of excellence: initial merits for planned clinical trials in Africa. *BMC Public Health* 13: 1

For peer review only - http://bmjopen.bmj.com/site/about/guidelines.xhtml

15. Cole DC, Johnson N, Mejia R, Mccullough H, Turcotte-Tremblay A-M, Barnoya J, Falabella Luco S. 2016. Mentoring health researchers globally: Diverse experiences, programmes, challenges and responses. Global Public Health 11(9): 1093-108
16. Enoch J. 2015a. Rapid mapping of international funders' research capacity strengthening priorities. UKCDS. http://www.ukcds.org.uk/sites/default/files/content/resources/UKCDS%20rapid%20RCS%20mapping%20for%20DFID.pdf (accessed 27 February 2017)
17. Enoch J. 2015b. Health Research Capacity Strengthening: A UKCDS Mapping. Published by UKCDS. http://www.ukcds.org.uk/our-work/health-research-capacity-strengthening-a-ukcds-mapping (accessed 27 February 2017)
18. Bates I, Boyd A, Aslanyan G, Cole DC. 2015a. Tackling the tensions in evaluating capacity strengthening for health research in low-and middle-income countries. Health Policy and Planning 30: 334-344.
19. Bates I, Boyd A, Smith H, Cole DC. 2014. A practical and systematic approach to organisational capacity strengthening for research in the health sector in Africa. *Health Research Policy and Systems* 12: 11
20. Cole D C, Boyd A, Aslanyan G, Bates I. 2014. Indicators for tracking programmes to strengthen health research capacity in lower-and middle-income countries: a qualitative synthesis. *Health Research Policy and Systems* 12: 17
21. Department for International Development (DFID) 2008. Research strategy 2008-2013 Working Paper Series: Capacity Building. https://assets.publishing.service.gov.uk/media/57a08b8ee5274a31e0000c0a/ResearchStrategyWorkingPaperfinal_capacity_P1.pdf ((accessed 27 February 2017)
22. Ghaffar DA, Ijsselmuiden C, Zicker F. 2008. Changing mindsets: Research capacity strengthening in low-and middle-income countries, Council on Health Research for Development (COHRED). http://www.who.int/tdr/publications/documents/changing_mindsets.pdf (accessed 27 February 2017)
23. ESSENCE (Revision 2016). Planning, Monitoring and Evaluation. Framework for Capacity Strengthening in Health Research. WHO. www.who.int/tdr/publications/Essence_frwk_2016_web.pdf (accessed 27 February 2017)
24. Kirkland J, Bjarnason S, Stackhouse J, Day R. 2006. International research management: Benchmarking programme. Report to the HEFCE by the Association of Commonwealth Universities.
25. Altbach PG, Salmi J. 2012. *The road to academic excellence: the making of world-class research universities*. Directions in development; human development. Washington DC: World Bank
26. Denzin NK, Lincoln YS. 2008. Collecting and interpreting qualitative materials. Published by Sage.
27. Golafshani N. 2003 Understanding reliability and validity in qualitative research. *The Qualitative Report* 8: 597-606
28. Cohen D, Crabtree B. Reflexivity. Qualitative Research Guidelines Project. http://www.qualres.org/HomeRefl-3703.html (accessed 27 February 2017)
29. Hyder AA, Zafar W, Ali J, Ssekubugu R, Ndebele P, Kass N. 2013 Evaluating institutional capacity for research ethics in Africa: a case study from Botswana. *BMC Medical Ethics* 14: 31
30. Gale NK, Heath G, Cameron E, Rashid S, Redwood S. 2013. Using the framework method for the analysis of qualitative data in multi-disciplinary health research. BMC Medical Research Methodology 13: 117

For peer review only - http://bmjopen.bmj.com/site/about/guidelines.xhtml

31. Bates I, Cole DC, Doyle V, Kelly E, Holler Johansen D. 2015b. Strengthening Research Management and Support Systems (RMSS) in African Universities. Overview Report for Four Universities to MCDC. September - October 2014. Accessed 27 February 2017 at http://www.mcdconsortium.org/sites/www.mcdconsortium.org/files/MCDC%20RMSS%20overview%20report%20FINAL%209jan15_0.pdf
32. Cunliffe AL. 2004. On becoming a critically reflexive practitioner. *Journal of Management Education* 28(4): 407-426
33. Quality Assurance Agency 2010. Code of practice for the assurance of academic quality and standards in higher education. http://www.bbk.ac.uk/linkinglondon/resources/apel-credit-resources/pub_Nov2010_QAA_COP_section_2_HE_guidance.pdf (accessed 27 February 2017)
34. Vitae, 2010. Research Development Framework. https://www.vitae.ac.uk/vitae-publications/rdf-related/researcher-development-framework-rdf-vitae.pdf/view (accessed 27 February 2017)
35. UK Research Councils, Arts and Humanities Research Board Joint statement of the Research Councils'/AHRB's skills training requirements for research students. http://www.rcuk.ac.uk/documents/skills/statementofexpectation-pdf/ (accessed 27 February 2017)
36. Jessani N, Lewy D, Ekirapa-Kiracho E, Bennett S. 2014. Institutional capacity for health systems research in East and Central African schools of public health: experiences with a capacity assessment tool. *Health Research Policy and Systems* 12: 21
37. Wellcome Trust. undated. Strengthening research ecosystems in Africa and Asia. https://wellcome.ac.uk/what-we-do/our-work/research-ecosystems-africa-and-asia. (accessed 27 February 2017)
38. Mashaah T, Hakim J, Chidzonga M, Kangwende RA, Naik Y, Federspiel N, Fiorillo S, Scott J, Gomo E. 2014. Strengthening research governance for sustainable research: Experiences from three Zimbabwean universities. *Academic Medicine* 89: S69-S72
39. Hwang K. 2013. Effects of the language barrier on processes and performance of international scientific collaboration, collaborators' participation, organizational integrity, and inter-organizational relationships. *Science Communication* 35(1): 3–31
40. Wirrman-Gadsby E. 2011. Research capacity strengthening, donor approaches to improving and assessing its impact in low- and middle-income countries. *International Journal of Health Planning and Management* 26: 89–106.
41. Ayah R, Jessani N, Mafuta EM. 2014. Institutional capacity for health systems research in East and Central African schools of public health: knowledge translation and effective communication. *Health Research Policy and Systems* 12: 20
42. African Academy of Sciences. Undated. Good Financial Grants Practice – Standardising, simplifying and strengthening governance of grant funding. http://aasciences.ac.ke/aas/en/academy/academy-pages/good-financial-grants-practice/ (accessed 27 February 2017)
43. World Health Organization Regional Office for Africa. Undated. WHO Guide for the Stepwise Laboratory Improvement Process Towards Accreditation in the African Region (with checklist). http://www.who.int/tb/laboratory/afro-slipta-checklist-guidance.pdf (accessed 27 February 2017)
44. Development Research Uptake in Sub-Saharan Africa (DRUSSA). Undated. Strategies for effective research uptake communication and impact. http://www.drussa.net/index.php?option=com_content&view=article&id=2788%3Astrategies-

For peer review only - http://bmjopen.bmj.com/site/about/guidelines.xhtml

for-effective-research-uptake-communication-and-impact&catid=211%3Atools-a-tips-open&Itemid=297&lang=en (accessed 27 February 2017)

45. Woolcock M, Narayan D. 2000. Social capital: Implications for development theory, research, and policy. *The World Bank Research Observer* 15: 225-249
46. Vogel I. 2012. Review of the use of 'Theory of Change' in international development. Report for the UK Department for International Development (DFID). August. https://assets.publishing.service.gov.uk/media/57a08a5ded915d3cfd00071a/DFID_ToC_Review_VogelV7.pdf (accessed 27 February 2017)
47. Boyd A, Cole DC, Cho DB, Aslanyan G, Bates I. 2013 Frameworks for evaluating health research capacity strengthening: a qualitative study. *Health Research Policy and Systems* 11(1): 46.
48. Peters DH, Adam T, Alonge O, Agyepong IA, Tran N. 2013. Implementation research: what it is and how to do it. *BMJ* 347: f6753
49. Creswell JW, Plano Clark VL. 2010. 2nd edition. Designing and Conducting Mixed Methods Research. Published by Sage https://us.sagepub.com/en-us/nam/designing-and-conducting-mixed-methods-research/book233508
50. Hansen JA, Lehmann M. 2006. Agents of change: universities as development hubs. *Journal of Cleaner Production* 14: 820-829

For peer review only

For peer review only - http://bmjopen.bmj.com/site/about/guidelines.xhtml

Table 1. Number of interviewees for baseline data collection by cadre and institution

	Institution number			
	**1**	**2**	**3**	**4**
Principal investigator	1	1	1	1
PhD students	5	1	3	0
Senior lecturer/lecturer/post-doc	3	1	2	2
Dean/registrar/ provost/principal	4	4	3	5
Head of Department	1	3	1	1
Research/ethics manager/administrator	2	4	1	2
Human resources staff	1	1	0	2
Finance/procurement staff	2	2	2	1
IT/library staff	3	3	4	4
Laboratory staff	0	2	2	1
Communications staff	0	0	1	0
**Total**	**22**	**22**	**20**	**19**

For peer review only

For peer review only - http://bmjopen.bmj.com/site/about/guidelines.xhtml

Table 2. Consolidated key gaps in research management and support systems (RMSS) and proposed actions, by RMSS component

Gaps	Proposed actions
**Research Strategies and Policies**	
• No research strategy or not available or publicised<br>• Lack of central tracking of research activities	• Departments/universities need an accessible research strategy with polices and guidelines to support its implementation<br>• Electronic research management support systems are needed to track proposals and projects and to document research income and disbursement including overheads
**Institutional Support Services and Infrastructure**	
• Lack of research support offices and/or insufficient coordination between departments and university levels<br>• Inadequate resourcing and lack of clarity about the role and long term financial sustainability of research support offices<br>• Research laboratory facilities are not accredited and lack over-arching planning to harmonise equipment purchase and maintenance across multiple short term projects<br>• Unclear relationship between hard copy library facilities and increasing use of e-resources	• The roles and relationships between university level research coordination and research support offices at Faculty or College level need to be clarified<br>• The strategy for research support offices at Faculty or College level needs to be clarified and mechanisms found for long term sustainability and buy-in by the researchers<br>• Achieve international laboratory accreditation for the institution’s own laboratories; harmonise research laboratories’ activities with those of affiliated organisations and establish clear processes and costs for researchers wishing to access these facilities<br>• Pro-actively plan the future of book libraries in the context of the shift to increasing use of e-resources, including their possible integration with ICT facilities
**Supporting Funding Applications**	
• Insufficient quality assurance checks and signing off processes for proposal submissions or contracts which could put the institution at risk of contractual or intellectual property issues	• Set up mechanisms for timely, multi-disciplinary (e.g. finance, legal, ICT, laboratory, library, procurement) input into proposal development<br>• Set up a formal process for quality assurance and authorisation of proposals before submission and for tracking the outcome of submissions
**Project Management and Control**	
• Senior researchers spend a substantial proportion of time on administrative, procurement and	• Establish an electronic research information system to systematically manage and track all aspects each project including the project agreement, protocol,

For peer review only - http://bmjopen.bmj.com/site/about/guidelines.xhtml

other issues that could be more effectively taken on by non-academic professional staff<br>• Lack of systems for tracking financial spend against budget for projects risks under- or over-spend<br>• Unclear lines of responsibility between researchers and finance officers regarding financial tracking and reporting	budgets, funding requirements, accounting and audit, and to maximise recoupment of overheads<br>• Establish a formal project approval process for successful applications, including and contract review and sign off<br>• Encourage researchers to include and budget for experienced administrators to help reduce the time they spend on project administration and to actively include other relevant inputs such as procurement expertise<br>• Provide joint training in financial management for researchers and finance officers and increase clarity and understanding about their various roles and responsibilities in relation to each other, the institution and the research funders
**Human Resource Management for Research**	
• Lack of clarity on contractual arrangements, and therefore institutional responsibility, for short-term project staff<br>• Poorly defined, or non-existent, career paths for non-academic professionals such as ICT, library and administrative staff<br>• No formal post-doctoral career posts for researchers	• Strengthen HR skills and structures so that they can better support researchers and research projects, and to ensure that project staff are university employees with access to the protection and facilities of the institution where this is not currently the case<br>• Formalise career tracks for research support staff<br>• Formal post-doctoral training programmes need to be established to develop and retain talented researchers
**Human Resource Development for Research**	
• No coordinated, institutionalised programmes for induction or research skills training for researchers; reliance on projects to provide training means focus is on technical skills rather than generic skills, such as leadership and research communication<br>• Training offered at university level (e.g. computer skills, literature searching) poorly publicised and utilised by researchers	• Provide a formal induction programme and training needs assessment for new research staff<br>• Establish an institutional programme of skills training for researchers, possibly through a dedicated unit, that includes non-technical skills such as leadership, supervision and project management<br>• Improve incorporation of existing training opportunities (e.g. provided by library and ICT staff) into a core skills training programme for researchers
**External Promotion of Research**	
• Promotion of research activities and successes by the universities not prioritised although widely recognised as important	• Review research section of universities' website to ensure information is current and that hyperlinks are working<br>• Consider setting up a unit specifically to enhance the visibility of institutional and/or departmental

For peer review only - http://bmjopen.bmj.com/site/about/guidelines.xhtml

	research activities and outputs<br>• Provide training in research communication to improve researchers' ability to write "jargon-free" communications such as press releases and policy briefs
**National Research Engagement**	
• Insufficient publicising of institutional research outputs in influencing national and international policy making and programming	• Explore options for improving researchers' ability to impact on national health research priorities and practices<br>• Universities and departments should systematically document and showcase national and international uptake and utilisation of the research findings they have generated

For peer review only - http://bmjopen.bmj.com/site/about/guidelines.xhtml

For peer review only

Table 3. Examples of progress on actions, challenges and innovative problem-solving during 2015-16, by RMSS* component and institution

Compo-nent [Overall progress]	Institution 1	Institution 2	Institution 3	Institution 4	Examples of innovative practices and problem-solving
**Research Strategies and Policies [little/no progress]**	University level strategy being approved but College level not yet developed.<br>A new university-wide research database now in place. A small university research fund has been set up.	Consultation on the strategy has taken place. A draft document is being written to submit for management approval.<br>New research management software has been installed to manage the research database	A research grant office is planned for early 2016 and a research database will be created within this office.	Strategy is on-hold as wide-scale institutional constitutional change is underway.<br>[post-project note: *Strategic plan 2017-2022 was approved December 2016*]	• Through a consultative process and workshop involving researchers, university officials, research support staff and representatives from external national agencies, the Ministry of Health and international research consortia, a research strategy was agreed based on a SWOT analysis
**Institutional Support Services and Infrastruc-ture [moderate progress]**	Work is on-going to coordinate inputs to proposals and grant management by PIs and finance staff. Workshops took place in 2015.	The first MoUs and SoPs are in development for grant related activities.<br>ICT policies for marketing and staff engagement are being developed.<br>Sustainability planning for lab resources and budgeting is underway.	A new high speed internet (DSL) has been installed at the Department of Biology, the PhD dedicated space and the Faculty library.<br>Steps towards formal accreditation of quality assurance	Progress has been made in establishing a research support office (grants unit) with administrators and financial staff appointed.	• A Laboratory Committee has been formed and inventoried all key laboratory resources within the College and affiliated research institutions

For peer review only - http://bmjopen.bmj.com/site/about/guidelines.xhtml

			processes and certification for the department labs have begun.		
**Supporting Funding Applica-tions [good progress]**	A university-wide database has been implemented.<br>The finance office is now involved in proposal development and joint training has taken place.	A new system for disseminating information about the research support services has been instigated.<br>"How-to" guideline on developing research proposals with a budget framework and checklists has been launched.<br>A research careers development webpage has been developed by the institution with materials for fledgling researchers	Implementation of a new research grant office and recruitment of a research coordinator has been agreed.	Administrators from the research/grants office have started assisting the PIs in proposal development, registering projects and tracking implementation and management.<br>The Grant office has been newly registered with several "calls application portals" and a database of researcher interests is being created.	• Production of a new "how-to" flow chart outlining the steps needed to develop research proposals<br>• A framework for calculating a proportion of staff time to be included in proposals for grants management has been established<br>• Topic-specific research groups have been formed which can respond to funding calls and collaboration opportunities<br>• A legal unit has been set up to guide the contract review process and a contract template and checklist developed
**Project Manage-ment and Control [good progress]**	Contract review, sign off and risk management procedures have been strengthened with contracts signed off by Provosts or Deans of	New systems for pre- and post-award management have been developed.<br>A new policy for compulsory post-award	SOPs for expenses and account management have been developed and validated with PIs, finance and	Project management procedures are now overseen by an administrator who manages the grant and liaises with the PI on the	• Standard operating procedures for expenses and account management have been developed jointly by researchers, finance and administrative

For peer review only - http://bmjopen.bmj.com/site/about/guidelines.xhtml

	Colleges.<br>Need for in-house training for admin to support PIs identified.	"Grants Management" inductions for all successful Principal Investigators is available.<br>A grants administrator has now been appointed. A new legal unit has been set up as part of research support services	administrative officers	implementation of the project.<br>A new financial management system PASTEL is now being used but staff need training to upload project data.	officers.<br>• New quarterly meetings between finance officers and researchers are used to address financial management of project grants
**Human Resource Manage-ment for Research [moderate progress]**	HR recruitment policies and procedures for project staff have been reviewed and developed.<br>A new staff development programme has been launched.<br>On-going meetings are continuing to discuss the need to develop a post-graduate program	A new training needs assessment for research support staff has been completed and training plans developed.<br>Procedures for hiring post- doctoral fellows have been established and terms and conditions of service defined.	The introduction of project management training courses and cross site visits for administrative staff and early career researchers have strengthened human resource management.<br>Staff needs for personal development have been identified but there is yet to be a plan to address these.	A team has been formed to develop a research scale based on a national scale for researchers.<br>Post- doctoral programmes are not yet institutionalised	• Career Development Centres have been established and stocked with training resources<br>• Human resources staff are being included in planning for research staff development
**Human Resource Develop-ment for Research**	Work is still on-going to plan a formal research-based induction process for project staff and	Work has begun towards a comprehensive competency- based capacity building programme for junior and	Progress made towards adaptation and institutionalisation of the doctoral research	The role of HR teams in research staff development has been reviewed and defined.	• An annual research training calendar has been developed for research staff based on a formal training needs assessment

For peer review only - http://bmjopen.bmj.com/site/about/guidelines.xhtml

**[moderate progress]**	fledgling researchers.<br>Mentorship training and pairing has taken place.	senior researchers.<br>A research capacity gap validation workshop and a training needs assessment for research staff have been conducted. A new annual research training calendar has been developed.	courses for researchers.<br>An external funder is working with the institution to plan personal development and leadership courses.	More work needs to be done to build a structured programme of capacity development for new and existing staff in research skills	• Regular training is now provided by the university on the roles and responsibilities of research leaders, administrators and finance staff throughout the grant life cycle
**External Promotion of Research [little/no progress]**	Limited training and mentoring in research communication provided by an external agency has started.<br>Some improvement of the research projects on the College website but more needs to be done to encourage researchers to add their details.<br>The academic board are to incorporate research uptake and the management of research outputs, into the university's centralised research	Research profiles of faculty are being introduced onto their webpages.<br>A separate research office website is under construction.<br>There are plans for a Knowledge Management Unit to help achieve better visibility of research activities.	The Faculty of Medicine website has been updated with current research projects.<br>The recruitment of a communication officer is planned, who will be responsible for supporting the publicising and dissemination of research activities and uptake.	The institution has an ongoing strong external collaborative network including an annual PhD symposium.<br>The planned new research strategy will embed research dissemination and uptake as a high priority area.<br>Work is ongoing to restructure the website	• An attractive annual university research report which chronicles recent research activities and highlights individuals, departments and colleges has been produced and is publically available

For peer review only

For peer review only - http://bmjopen.bmj.com/site/about/guidelines.xhtml

	policy.				
**National Research Engage-ment [little/no progress]**	Mechanisms still need to be established at College and departmental level to promote, monitor and record communication and to share opportunities for engagement between projects.<br><br>Continuation of institution's work with multi-national partners on national research engagement to feed into the College's research strategy.	Key national health policy making forums have been targeted with specifically-designed materials.<br><br>A local travel grant system is being established to support researchers to attend national meetings.<br><br>A template has been developed for annual research reports, to highlight successes, failures and opportunities for policy engagement	Ongoing participation in national tailored monitoring and evaluation training courses for medical district officers and health supervisors where research results and experience will be shared	Researchers continue to attend a national research conference (e.g. in 2015) to disseminate their projects.<br><br>Promotion of their research into policy and practice is not yet stated in the research strategy as a core element of a researchers' responsibility	• A university research uptake strategy has been published<br>• University regularly hosts a meeting of leading thinkers in science, policy, industry and civil society in Africa

*RMSS = research management and support system

For peer review only - http://bmjopen.bmj.com/site/about/guidelines.xhtml

For peer review only

Supplementary Data

Box 1 Key sources used to inform review tools

Optimal institutional research management and support

- Ajai-Ajagbe, P. (2013) #13 Research Management in African Universities. *IAU Horizons* (June) Vol. 19 N° 2 pp 32-33. http://www.acup.cat/sites/default/files/iauhorizonsvol192en1.pdf
- Bates I, *et al*. 2011. Assessing and Strengthening African Universities' Capacity for Doctoral Programmes. *PLoS Medicine.* 8(9).
- Cole DC, Boyd A, Aslanyan G, Bates, I. 2014. Indicators for tracking programmes to strengthen health research capacity in lower- and middle- income countries: a qualitative synthesis. *Health Research Policy and Systems.* **12(17).**
- Dean O, Smith (2011). Managing the Research University. Oxford University Press. ISBN 978-0199793259. Retrieved 2014-08-11.
- Dyason K, Harle J. Research project management in African universities. RIMI4AC. Available online at: http://www.rimi4ac.net/site_media/CONTENT_uploads/ProjectManagement_v1%20[SINGLE%20PAGES].pdf (accessed on 13/08/2014).
- ESSENCE, 2011. Planning, Monitoring and Evaluation. Framework for Capacity Strengthening in Health Research (pdf). TDR/WHO: Geneva. Available online at: http://whqlibdoc.who.int/hq/2011/tdr_essence_11.1_eng.pdf (accessed on 16/05/2014).
- ESSENCE, 2012. Five keys to improving research costing in low- and middle- income countries (pdf). TDR/WHO: Geneva. Available online at: http://whqlibdoc.who.int/hq/2012/TDR_ESSENCE_1.12_eng.pdf?ua=1 (accessed on 16/05/2014).
- Green J, D Langley (2009) Professionalising research management Bristol: HEFCE. A report to HEFCE
- HEFCE (2008) Institutional Benchmarking Template: Establishing and meeting the leadership and development needs of principal investigators
- Kirkland J, Bjarnason S, Stackhouse J. 2006 International research management: benchmarking programme . Report to HEFCE by the Association of Commonwealth Universities. Available online at: http://webarchive.nationalarchives.gov.uk/20100202100434/http://www.hefce.ac.uk/pubs/rdreports/2006//rd11_06/rd11_06.pdf (accessed on 13/08/14)
- Kirkland J, Ajai-Ajagbe, P. 2013. Research management in African universities: from awareness raising to developing structures**.** The association of commonwealth universities (ACU). Available online at: http://carnegie.org/fileadmin/Media/Publications/PDF/ACU_-Research_Management_in_African_Universities_Report_-_2013.pdf (accessed on 02/08/2014, no longer on-line).

- Mirzoev T, Lê G, Green A, Orgill M, Komba A, Esena R, Nyapada L, Uzochukwu B, Made W, Nxumalo N, Gilson L. 2013 Assessment of capacity for Health Policy and Systems Research and Analysis in seven African universities: results from the CHEPSAA project. *Health Policy and Planning*. 10.1093/heapol/czt065
- QAA. 2012. UK Quality Code for Higher Education (pdf). QAA: Gloucester. Available online at: http://www.qaa.ac.uk/Publications/InformationAndGuidance/Documents/B10.pdf (accessed on 16/05/2014).

Cross institution/Country wide research management

- Meek & Olson. Effectiveness of Research and Innovation Management at Policy and Institutional Levels. OECD. (undated) http://www.oecd.org/sti/Effectiveness%20of%20research%20and%20innovation%20management%20at%20policy%20and%20institutional%20levels_Meek%20and%20Olsson.pdf

The structure of the observation checklists, interview guides and the phrasing of some of the questions were based on previously designed evaluation tools, specifically those from the following references:

- Benchmarking Southern African Universities 2014 DRAFT Summary Association of Commonwealth Universities. Available online at: https://www.acu.ac.uk/focus-areas/benchmarking-african-universities (accessed on 13/08/14)
- Kirkland J, Bjarnason S, Stackhouse J. 2006 International research management: benchmarking programme . Report to HEFCE by the Association of Commonwealth Universities. Available online at: http://webarchive.nationalarchives.gov.uk/20100202100434/http://www.hefce.ac.uk/pubs/rdreports/2006//rd11_06/rd11_06.pdf (accessed on 13/08/14)
- Unpublished documents (2014) from RS-DFID Africa Capacity Building Initiative Evaluation: Toolkit (Draft)
- Unpublished documents (2013) from the Royal Society- Leverhulme African Award Evaluation: interview guides, survey methodology, findings report.
- Unpublished documents (2013) from the HRCSI Evaluation 2013: Interview guide package.
- Unpublished documents (2012) from Enhancing Laboratory Capacity Strengthening for NTD control, Deliverable One: tool for capacity gap analysis, interview guides.

For peer review only

Box 2. List of topics/items relevant to university research management and support systems (RMSS), grouped by components

University Research Strategy
The university has a research strategy
The research strategy is framed within the overall goals of the institution. The strategy is distinct from but links clearly with, and is complimentary to, other institutional plans, strategies and policies
The research strategy explicitly states its purpose to assist the business of the institution, identifies priorities, and monitor progress
The institution's mechanism for determining research strategy is transparent and widely owned
The institutional research strategy fully involves faculties in its design and implementation, and policies carried out by individual schools or departments are consistent with it
Implementation of the research strategy is overseen by an appropriate member of senior management. The strategy is also backed up by appropriate manpower & resources, to make sure it is implemented
The research strategy has the facility to draw on a range of evaluation mechanisms which might include sources external to the university - such as external peer review including other universities
The Research Management Office [if it exists] is fully involved in the drafting of institutional research strategies in conjunction with other appropriate offices
The research strategy is underpinned by the internal funding mechanisms for research
The research strategy is, as far as possible, responsive to the research funding environment and opportunities (at national, international and regional levels)
The research strategy seeks to add value to existing activity by proactively highlighting new opportunities for internal and external collaboration. The strategy should also promote interdisciplinary research and the development of early career researchers
The research strategy is effectively communicated, monitored, reviewed and developed/refined
Methods for evaluation of the strategy and performance indicators should be established from the outset. Key performance indicators should include a balance of quantitative and qualitative methods
The research strategy should be sufficiently flexible and defined within a reasonable time frame (e.g. 5 years) reviewed regularly, and be capable of evolving in response to events
The strategy should take into account the need for appropriate staff incentives
**Institutional Research Capacity**
The institution has a unit dedicated to research management (Research office)

For peer review only

The institution has a research committee to develop strategies to assist the University in meeting its research objectives, identify priorities, advise on stakeholder engagement, monitor research performance, discuss annual department updates, monitor national and international research policy which effect the institution<br><br>The research unit has an adequate number of Staff to fulfil the needs of researchers<br><br>Culture where research is valued, accepted, encouraged and enjoyed<br><br>Ethics committee exists to ensure research conducted is ethical<br><br>Evidence of accessible guidance to help researchers through the research process including governance and ethics<br><br>Clear academic honesty guidelines in place<br><br>Able to meet requirements of GCP and GLP<br><br>Sufficient facilities are available for research (studying, IT, library, technology, laboratories etc)
**Supporting funding applications**
Institutions have regular, effective and proactive means of informing academics staff on funding opportunities and the strategic directions of funding agencies where possible, these should involve direct communication between the Research Office and individual staff<br><br>Central research Offices have developed and strategically use key contacts in faculties schools, institutes and departments to facilitate a two way flow of information in funding opportunities and research interest<br><br>The institution maintains a searchable database on institutions research performance, capabilities and contact, including all past projects and proposals<br><br>information and current policy from all funders is maintained and communicated as appropriate<br><br>The Research Office holds regular information and updating sessions and targeted workshops for faculty members and graduate students with the purpose of providing information on funding opportunities, proposal development and the development of collaborative research teams to respond to one-off as well as on-going research opportunities<br><br>The institution seeks to establish an effective two-way communication strategy between themselves and major sponsors and proactively seek to develop that relationship<br><br>The institution has clear mechanisms in place to handle internal external enquiries regarding possible research and consultancy opportunities and to monitor the outcomes of these on a regular basis<br><br>The Research Office actively encourages collaboration between different departments within the institution including senior Academic Office, Public Relations, Marketing and Registry<br><br>The institution seeks to develop mechanisms to effectively track and involve alumni working in key positions with current, past and potential sponsors and in government

For peer review only

The research office actively brings key staff together in response to large scale tender and proposals proposal requests where appropriate and where consistent with research strategy<br><br>Institutions through the Research Office or other appropriate office, ensure that proposals are reviewed by experienced academic and research staff (externally, where appropriate) prior to submission<br><br>Proposals are only submitted with clear support from Head of Department of other appropriate management authority. Key personnel who need to be aware' of the project should be notified.<br><br>The institution approves all proposals before submission and research offices maintain records on the progress of all proposals<br><br>The information gained from previously submitted proposals is used to inform future proposals<br><br>The institution has a clear transparent and widely disseminated formula for determining the full economic cost of any give project, including indirect costs and staff time<br><br>full costing is calculated for each externally funded project even if this is not reflected in the price charged<br><br>All proposed research should be consistent with the institutions overall research strategy<br><br>The institutions provides clear guidance to staff and external sponsors as to which kind of projects and contractual terms are acceptable<br><br>The institution has clear risk assessment procedures for proposed projects which recognise the need to involve several key offices within the institution<br><br>The institution systematically reflects on its progress against its research strategy including regular comparisons with other institutions of similar nature
**Project Management and control**
All project proposals contain explicit statements of how the project will be managed and, where possible and appropriate, provision for the appointment of specialist staff<br><br>Mechanisms are in place to recognise the critical role of Principal Investigators, to ensure that they and other key actors are aware of their roles and responsibilities before commencement of the project and where required, that appropriate training is undertaken.<br><br>Key milestones (including reporting and financial review dates) are agreed with key actors at the outset and updated amongst all those actors throughout<br><br>Key actors, including Principal Investigators and Deans, are provided with regular and up to date project information (including financial, human resources, IP, and commercialization information), through on-line access or regular statements<br><br>Information provided to key actors, including Research Officers and Deans, pro-actively highlights any risks and obligations specific to both them and the institution.<br><br>Procedures are in place to ensure that all those with access to research are covered by appropriate

For peer review only

confidentiality and rights assignment agreements (depending on jurisdiction), particularly those who are covered by a contract of employment with the institution

Appropriate data management policies exist (covering ethical and legal compliance, copyright and IPR issues, data storage, security, sharing and retention)

Appropriate health and safety policies are in place (encompassing staff induction, safety officers, evacuation procedures etc)

Appropriate insurance arrangements are in place for both staff and clinical trials (if applicable)

Mechanisms are in place to ensure that intellectual property both brought to and emerging from research is identified, protected, tracked and signed off at all stages and that staff have access to specialist advice in this regard.

Procedures are in place for the appropriate monitoring of material transfer agreements.

Mechanisms are in place to identify possible delays and monitor expenditure to ensure it is in line with project budgets

The institutions has an explicit consistent framework within which academic units can predict future revenue and expenditure, especially where such income contributes to underpinning core activities

Mechanisms are in place for the disclosure and management of conflicts of interest.

Mechanisms are in place to obtain feedback project sponsors, which can be taken into account in future planning

Formal closure and continuous monitoring processes are in place ensuring that all obligations have been and continue to be met and that opportunities arising from the project are identified.

Training and staff development for research

Evidence of research training needs assessments

Provision of research skills training shaped around skills background and needs of different professional groups

There is availability and use of funds for research skills training for research management staff, researchers and academic staff

There is availability of a range of research skills training for students, research management staff and researchers covering-

Proposal writing, grant application, data analysis and management (including software and qualitative analysis) ethics, health and safety, GCP and GLP, generic research skills (quantitative and qualitative) academic writing and publishing

Evidence of matching novice and experienced researchers

Mentorship and supervision structures for students and early career stage researchers and new PI’s

For peer review only

Individual job descriptions support research/institutional objectives<br><br>Policies are in place to support recruitment and contract negotiation for new support staff<br><br>Staff skills and abilities are matched to research needs<br><br>Seminar programmes relating to research undertaken<br><br>The research management structure and policies form a core element of induction programmes for new academic and technical staff as well as new postgraduate students.<br><br>Research strategy, policy and management issues form a core element of ongoing professional development programmes for mid-career and senior academic staff.<br><br>Staff in leadership roles (e.g. Deans) are offered appropriate instruction in research strategy, policy and management, as well as being involved in discussion of good practice within the institution<br><br>The Research Office maintains effective ongoing relationships with internal clients at all levels (faculty, department, individuals) with a view to supporting research staff and understanding their needs.<br><br>Performance measures for research management are established and are widely available/disseminated.<br><br>The institution makes provision for appropriate incentives to enhance the research activity of new and emerging researchers. Such incentives might include conference grants and other start-up funding.<br><br>Policies for providing incentives for staff research activity are transparent, easy to understand and consistent across the institution.<br><br>Career development opportunities<br><br>Career pathways exist for researchers
**Teaching capacity to support research**
Number of (half as a minimum) full-time academic staff as active and recognised contributors to subject associations, learned societies and relevant professional bodies.<br><br>Number of (third as a minimum) academic staff with recent (i.e. within the past three years) personal experience of research activity (including external examination, review panels, collaborative research)<br><br>Number of (third as a minimum)academic staff engaged in research or other forms of advanced scholarship<br><br>The outcomes of external scrutiny exercises undertaken by bodies such as the Quality Assurance Agency for Higher Education, the funding councils and professional and statutory bodies are carefully considered and actioned.
**External promotion of research**

For peer review only

Collaborations exist with external organisations (institutions, businesses, government, NGO's)
The institution is able to conform to the requirements of multiple funding agencies
Number of joint posts with other academic institutions
The institution has a clear strategy in place for all forms of intellectual property management
Clear regulations are in place to determine the ownership of intellectual property by and between staff, students and third parties. These regulations are effectively disseminated throughout the institution and externally
Academic departments and research projects are systematically monitored to identify emerging intellectual property at an early stage.
The institution establishes a register of intellectual property assets and pro-actively manages and maintains it at all stages of development and exploitation
Clear policy mechanisms are in place to govern the distribution of revenues from intellectual property between the university and other key stakeholders.
The institution's research communication strategy is consistent with the institution's overall strategy and underpins the core missions of the institution, particularly in relation to the integration of research, education and service.
There is a clear understanding of the roles and responsibilities of the different offices/officers responsible for research communication and good channels of communication exist between all these actors.
The institution pro-actively identifies projects (at various stages) and outcomes that are aligned with the university's priorities and are particularly suitable for external dissemination
The institution has a programme of events, such as launches, to profile major achievements or projects which relate to the strategic objectives and any priority research themes of the institution.
The institution has clear criteria for the type of work most likely to generate good publicity, and guidance on how to avoid poor publicity, and makes this information available to staff.
The institution has a clear strategy and procedures with regard to handling crisis communications and ensures these are disseminated to every level.
The institution seeks to make key research findings accessible to a wider audience, through the use of research summaries, expert guides and speakers lists, produced in suitable lay language and in publicly accessible formats so as to engage public understanding of the core mission of the institution (including inter-institutional partnerships).
The institution has established clear mechanisms to review and reward the performance of departments and research groups in the area of dissemination, which are integrated with an incentivisation policy providing a variety of incentives.
Mechanisms are in place for staff to report their dissemination activity. Such mechanisms maximise

For peer review only

research kudos and academic excellence and are consistent with any reporting requirements to external organisations<br><br>The institution provides assistance and systematic training programmes for staff in handling the media, and specific assistance in the drafting of press releases and publicity materials.<br><br>The institution facilitates the participation of researchers, particularly early career researchers, in international conferences and other fora to present their research findings and raise their profile<br><br>Where possible, dissemination outputs of staff are captured in a centrally managed integrated digital repository, linked to any central research activity database, which is made available to all units of the institution<br><br>The institution has a clear branding policy which is consistent with the research communication strategy.<br><br>The institution's web portal reflects the institution's core mission and strategy and is strategically and systematically managed as a key tool for promoting research to the broader community.
**National Research Uptake**
Ability of link policy to research and practice<br><br>Number of evidence based policies<br><br>Number of evidence based development interventions<br><br>Number of plans and policies to support research<br><br>Existence of national standards (accreditation, quality assurance) regarding the evaluation of research institutes<br><br>Existence of scientific councils with transparent and efficient systems in place to evaluate and disburse competitive research funds<br><br>Level of funding of research by the government<br><br>Researcher salary on par or above other countries in region

For peer review only

Box 3. Overall semi-structured interview suite of questions, by RMSS component

Biography of interviewee

What is your current position within this institution?

How long have you held this position?

How long have you worked at this institution?

What is your role in research within this institution?

1. Research strategies and policies

The institution

How many staff and students are there at this institution?

What is the percentage of income from a) teaching and b) research?

Is there core funding for research? How much? How is it disbursed?

How many PhD students are registered a) with your institution and b) externally?

Is there a university officer/directorate responsible for research? Do they have terms of reference?

How does this institution's research outputs compare to other comparable institutions? How do you measure this?

Strategies

Do you have a university research strategy?

What are the main themes/components of the strategy?

Does it link to a) national and b) other institutional strategies?

How is it disseminated internally and externally?

What are the research strengths at this institution?

Are strategies revised? How often? What were any major changes?

What are the strategic priority research areas? How were they decided? How are researchers and externally funded projects encouraged to focus on these areas?

Was any baseline information (e.g. a SWOT analysis) used to inform the strategy?

Who was involved in setting the strategy? What was the process?

For peer review only

2. Institutional Support Services and Infrastructure

Research management

Is there a university research committee? What do they do?

Is there a research support office? What do they do? (E.g. identify opportunities, help with application process, and ensure compliance with funder's requirements)

How are you made aware of research funding opportunities? (at national, international and regional levels)

How do you keep track of publications/presentations/conferences/grant applications produced/ department?

ICT (also see data management in section 4)

Is there adequate Wi-Fi, broadband speed, video conferencing and skype facilities for researchers? Do they pay for this?

Can they access the IT systems from home?

Do you purchase computers etc. on their behalf, or make recommendations? Do you set them up? Help with software? Is there any charge for this service?

How are files and information backed up? (e.g. offsite servers)

Library

How do staff and students access peer reviewed and grey literature? Are there any regular training courses offered?

How is access to e-resources and hard copy books/journals managed between the ICT unit and the library?

Laboratories

What research laboratories and field sites are available to use for research purposes at this institution?

What type of research studies can be supported by the laboratories (e.g. HPLC for pharmacokinetics; genomics/sequencing; insectory etc.)

Are the laboratories enrolled in external quality assurance systems?

Do the laboratories have international accreditation?

Do the laboratories follow Good Laboratory Practice guidelines?

Are there backup generators? Surge protection?

What sample storage facilities do you have? Are they temperature controlled and monitored?

What are the policies and processes governing transfer of samples to external institutions?

For peer review only

3. Supporting funding applications

What are the mechanisms for identifying external funding opportunities? Does the research office help with this? (E.g. is there a 'grants identification' officer?)

Is there any support to help PIs prepare funding proposals? (E.g. getting documents together, preparing/checking budgets, submitting proposals)

What is the mechanism for collating information on all proposals submitted? Is there a searchable database of submitted projects and whether they were successful?

What is the process for submitting proposals? Is there a formal sign off and if so by whom?

Do proposals have to have input or approval from finance/accountants prior to submission? What do they look for? How do you make sure that overheads are included and the costings are correct (e.g. salaries, equipment)?

Does the university use external advice (e.g. legal) at any stage during the process?

Do you have any way of comparing your research performance with other institutions?

4. Project management and control

What systems are in place to monitor the progress of each project? (E.g. against milestones)

Ethics

How is this managed in the university as a whole? Is this done at the university or at the faculty level?

Is ethics committee membership GCP-compliant?

Are there guidelines about how the ethics committee functions?

Are there guidelines for researchers about the ethics process?

Are there guidelines relating to academic honesty and plagiarism?

Financial

Who provides financial reports to funders? Who has specialist knowledge of each funders' reporting requirements?

How often are financial reports made to PI's (frequency, method, feedback loop?)

How do departments predict and plan future research revenue and expenditure?

How does the university ensure that project expenditure remains in line with the budget?

Legal

For peer review only

What is the process for minimising risks regarding financial and contractual terms? Is legal advice available? Who accesses this and when? (ie during the contract signing process or only if there is a problem)?

How are appropriate insurance arrangements organised (particularly for field staff and clinical trials)

What regulations are in place to determine the ownership of intellectual property by and between staff, students and third parties? How are these regulations disseminated throughout the institution and externally?

If what ways do you identify emerging intellectual property in your academic departments and on-going research projects?

Have you established a register of intellectual property assets? How are these managed and maintained?

What policies/mechanisms are in place to govern the distribution of revenues from intellectual property between the university and other key stakeholders?

Data management

Are there research data management guidelines and/or policies for data protection and storage?

How is research data backed up and secured? How are routine office and research documents (e.g. draft publications, guidelines/protocols etc.) backed up and secured?

Who is responsible for these systems? Are PIs charged for this service?

Do you provide help for PIs to complete Data Management plans to funders?

What are the mechanisms for managing data ownership, data security, licensing for re-use, data sharing, reuse of third-party data, restriction of data sharing (prior to publishing or seeking patents, retaining/destroying data?

Clinical work/trials questions:

Does the university acts as a sponsor for clinical trials?

Is there a clinical trials office? What does it do?

How do you do clinical monitoring? Have any audits about this been conducted and if so what were the key findings?

5. Human Resource Management for Research

Are job descriptions available for researchers and support staff?

Is there an induction process for new employees?

What are the processes for promotion for a) researchers and b) support staff (e.g. administrators, laboratory scientists)?

For peer review only

What mentorship and supervision structures exist for students and early career stage researchers and new PI's?

What career pathways are there are for a) researchers and b) research support staff?

Is there any ongoing professional development programme and does this cover research skills?

Do you know about the MCDC career development groups (CDGs)?

How do the MCDC career development groups (CDGs) fit into/complement institutional systems?

Are these career development activities embedded in institutional structures?

Do you think they are helpful? Should they be institutionalised? Why/why not?

Human resources

What Policies/strategies are in place for Human resource development of a) researchers/scientific staff, b) admin staff (including training, retention, tenure track, funding)

Do you have a formal induction process for new employees? Is there a special one for researchers?

Are there health and safety policies? (E.g. staff induction, safety officers, evacuation procedures etc.)

How are training needs identified? (E.g. staff training needs assessments). Is HR responsible for providing and/or recording any research training (e.g. GCP/GLP training, proposal writing, project management, supervision)?

How are training opportunities identified and funded? Is there a core budget for training and how is it allocated?

What proportion of research posts are a) core funded and b) project funded?

Are you involved in all new appointments? Do you advise PIs on the institution's procedures governing the employment of staff?

Is career guidance given to PhD students, post-docs and other researchers?

How are post-docs absorbed into the workforce?

What is the process and turnaround time for recruiting and appointing new research staff?

Do you make more internal or external research appointments?

Does your institution offer the possibility of short-term bridging funding to retain research staff during hiatus periods between grants?

Can you describe what Performance measures are used for research management and how these are reported?

Are there joint posts with other academic institutions? How do they work and are they effective?

For peer review only

6. Human Resource Development for Research

Is training available on

- Research design (epidemiology, stats, social science, health systems)
- Ethics, health and safety, GCP and GLP
- Data analysis and management (including software and qualitative analysis)
- Academic writing and publishing
- Proposal writing, grant application
- Teaching and education
- Leadership and management

Are there facilities and fora (e.g. seminars, journal club, staff exchanges) for researchers to discuss their work regularly with each other?

Is a tracking system in place for PhD students? How many supervisors do PhD students have? How many students do PhD supervisors have?

Are there minimum standards in place about the level of supervision to be given?

7. External promotion of research

Do you have longstanding research collaborations with external organisations? Examples?

Do you have a research communication strategy? A research communication unit?

Who is responsible for research communication? Do researchers perceive that this is their responsibility?

Does the institution have a research profile on its website?

How do you make key research findings accessible to a non-academic audience (e.g. research summaries in lay language and in publicly accessible formats)?

Do you have a programme of events, such as launches, to profile major achievements or projects

Do you provide advice to staff about how to deal with the media (e.g. how to generate good publicity and avoid poor publicity)?

What strategy and procedures are communicated to staff with regard to handling crisis communications and how are these disseminated?

Are there incentives for departments and research groups in the area of dissemination?

8. National research engagement

What level of funding for research is provided by the government?

For peer review only

Are there national policies and plans regarding health (and other) research?

How does the institution engage with policy makers?

Are there national scientific councils that evaluate and disburse competitive research funds? Do they have transparent and efficient systems?

What are the mechanisms by which research from your institution influences policy and practice?

Are there national standards (accreditation, quality assurance) regarding the evaluation of research institutes?

For peer review only

BMJ Open

A qualitative study to develop processes and tools for the assessment and tracking of African institutions' capacity for operational health research

Journal:	*BMJ Open*
Manuscript ID	bmjopen-2017-016660.R2
Article Type:	Research
Date Submitted by the Author:	31-May-2017
Complete List of Authors:	Wallis, Selina; Liverpool school of tropical medicine, International health<br>Cole, Donald; University of Toronto, Dalla Lana School of Public Health<br>gaye, oumar<br>mmbaga, blandina<br>mwapasa, victor<br>tagbor, harry<br>bates, imelda
**Primary Subject Heading**:	Global health
Secondary Subject Heading:	Global health
Keywords:	Research activities, Institutional practices, Systems analysis, Organization & Administration, Evaluation

SCHOLARONE™
Manuscripts

For peer review only

A qualitative study to develop processes and tools for the assessment and tracking of African institutions' capacity for operational health research

Selina Wallis, Donald C Cole, Oumar Gaye, Blandina T. Mmbaga, Victor Mwapasa, Harry Tagbor, Imelda Bates

Corresponding author's name, address, telephone/fax numbers and e-mail address;

Imelda Bates, Liverpool School of Tropical Medicine, Pembroke Place, Liverpool L3 5QA

Tel: 0151 705 3115; Imelda.bates@lstmed.ac.uk

Keywords [MeSH] Research activities, Institutional practices, Systems analysis, Organization & Administration, Evaluation

Abbreviated Running Title - Assessing research management systems

Abstract

Objectives: Research is key to achieving global development goals. Our objectives were to develop and test an evidence-informed process for assessing health research management systems (RMSS) in four African universities and for tracking interventions to address capacity gaps.

Setting: Four African universities

Participants: Eighty-three university staff and students from eleven cadres

Intervention/methods: A literature-informed 'benchmark' was developed and used to itemise all components of a university's health RMSS. Data on all components were collected during site visits to four African universities using interview guides, document reviews and facilities observation guides. Gaps in RMSS capacity were identified against the benchmark and institutional action plans developed to remedy gaps. Progress against indicators was tracked over 15 months and common challenges and successes identified.

Results: Common gaps in operational health research capacity included: no accessible research strategy, a lack of research e-tracking capability, and inadequate quality checks for proposal submissions and contracts. Feedback indicated that the capacity assessment was comprehensive and generated practical actions, several of which were no-cost. Regular follow-up helped to maintain focus on activities to strengthen health research capacity in the face of challenges.

Conclusions: Identification of each institutions' strengths and weaknesses against an evidence-informed benchmark enabled them to identify gaps in in their operational health research systems, to develop prioritised action plans, to justify resource requests to fulfil the plans, and to track progress in strengthening RMSS. Use of a standard benchmark, approach and tools enabled comparisons across institutions which has accelerated production of evidence about the science of research capacity strengthening. The tools could be used by institutions seeking to understand their strengths and to address gaps in research capacity. Research capacity gaps that were common to several institutions could be a 'smart' investment for governments and health research funders.

For peer review only - http://bmjopen.bmj.com/site/about/guidelines.xhtml

Trial registration: none

Strengths and limitations of this study

- This study uses qualitative research to generate primary, prospective, longitudinal data about the baseline status of operational health research systems in four African institutions, and tracks changes in research capacity against pre-determined indicators
- The use of the same benchmark and research approach across different institutions enables comparisons to be made so common challenges can be identified; these could be effective targets for investment
- The main limitations for the study were that the limited follow up time did not allow for demonstration of the long-term sustainability of changes to research systems and, because our study was designed to provide a broad overview of an institution's RMSS, it did not explore particular components in depth
- Institutions found the evaluation process to be comprehensive and helpful since in addition to advancing the science of research capacity strengthening it generated practical actions and progress indicators, and facilitated inter-institutional comparison and bench-marking

Source of funding: This work was supported by a grant from the Wellcome Trust, UK, to the London School of Hygiene and Tropical Medicine MCDC project (http://www.mcdconsortium.org/phd-programme.php). The funders had no role in study design, data collection and analysis, decision to publish, or preparation of the manuscript.

Competing interests

We have read and understood BMJ policy on declaration of interests (http://www.bmj.com/about-bmj/resources-authors/forms-policies-and-checklists/declaration-competing-interests) and declare that we have no conflicts of interest

Word count: 4602 excluding authors' contribution statement, tables, figures/diagrams and references

For peer review only - http://bmjopen.bmj.com/site/about/guidelines.xhtml

Process and tools for the assessment and tracking of African institutions' capacity for operational **health research**

INTRODUCTION

Importance of research for development

Health research has been acknowledged to play a key role in progress towards the Sustainable Development Goals.[1] Strong research institutions and skilled researchers are essential for low- and middle-income countries (LMICs) to generate evidence for their own health policies and to make progress in achieving their health related goals.[2, 3] Investments in operational health research capacity, can provide positive returns by promoting evidence-informed policy and practice in the health system,[2] although implementation[4] and estimation of returns can be challenging.[5] The first African Ministerial Conference on Science and Technology in 2003 recognised that "There is strong evidence that using research evidence to inform policy and practice leads to benefits which contribute to socioeconomic development"[6] and participating countries committed to spend at least 1% of their GDP on research and development by 2010.[7] Only Kenya, Malawi and South Africa have managed to approach this target and Kenya, Mozambique, Senegal and Uganda all have more than 40% of their research and development financed from abroad.[7]

Lack of research/researchers in LMICs especially in Africa

Although the average growth rate of scientific production in Africa is faster than that of the world as a whole, African Union countries only produce 2% of the world's total scientific output.[8] Egypt, Kenya, Nigeria, and South Africa produce the largest number of publications from Africa.[7] This is a reflection of the small numbers of researchers in Africa and decades of under-investment in research institutions. Most countries in sub-Saharan Africa (SSA) have less than 500 researchers (of all disciplines) per million inhabitants (e.g. Tanzania 35, Ghana 39, Malawi 50, Senegal 361) compared to over 4000 per million inhabitants in the UK and North America.[9] There are numerous disincentives to pursuing a research career in many African countries including heavy teaching loads, weak organisational research systems, lack of national research leadership, limited access to scientific information, slow internet connections and inadequate physical facilities including libraries and laboratories.[10]

Attempts to address weak capacity for operational health research in Africa

Resources to guide development of operational health research capacity have been available for at least a decade[11] but outdated and ineffective models for strengthening capacity persist.[12] African research institutions have historically faced numerous challenges.[13] The ability to produce international quality health research depends not only on developing a critical mass of African researchers, but also on providing them with a conducive environment in which to do research and progress their careers.[14, 15] International funders have responded by supporting strengthening of national systems and structures for operational health research[16] and in boosting the capacity of low- and middle-income country universities in research governance and management.[17] However, despite longstanding calls for more robust evaluations of capacity development,[11] the evidence needed to inform effective implementation and evaluation of programmes for strengthening operational health research capacity remains weak.[18, 19] Furthermore, the lack of clearly defined goals and baselines against which to evaluate the success of research capacity strengthening programmes makes it difficult to track their progress and impact.[20]

For peer review only - http://bmjopen.bmj.com/site/about/guidelines.xhtml

Development funders and policy makers are calling for a "significant re-think of the approach to capacity development".[21] They stress the need for an inter-disciplinary approach which recognises the complexity, fluidity and non-linearity in human systems, a systematic perspective, and acknowledgement of relationships between capacity at the individual, institutional and wider societal levels.[22, 19] To promote a more purposeful and strategic approach to strengthening operational health research capacity in LMICs, a group of international funders have produced guidance about developing shared principles and indicators,[23] and for evaluating outcomes and impacts of health research capacity strengthening interventions. Putting these guidelines into practice at the organizational level is challenging since little is known about what information matters for strengthening research capacity, and how and why this varies in different institutional contexts.

Purpose

The purpose of our study was to develop and test an evidence-informed process that could be used 1) to conduct a baseline assessment of operational health research management and support systems (RMSS) in four African universities and 2) to document actions taken to address identified gaps. As institutions implemented these actions, we sought to identify common difficulties they encountered. This information would help not only the institutions, but also external agencies and national governments, to more effectively target and monitor their contributions to strengthening institutional and hence, national health research capacity. The assessment process covered all the components needed for a university to generate, manage and disseminate operational health research of international quality.

Approach to the study

The study comprised three phases – construction of a benchmark against which to conduct the baseline assessments of institutions' RMSS, development of data collection tools based on the benchmark and collection and analysis of data during visits to the institutions and the follow up period. Despite earlier work on research management benchmarking,[24] no single document existed which detailed all the systems needed in a university to foster, support and manage international quality operational health research. Hence it was necessary to develop a comprehensive description of the components of an 'optimal' scenario[19] as a benchmark against which the baseline assessment could be compared.[22, 25] We describe the process of using best available evidence to generate this benchmark as a health Research Management and Support Systems (RMSS) list and used the benchmark to craft tools for collecting baseline data in each of the universities and to collate a list of indicators for monitoring progress. We share our experience of using the tools to identify institutions' RMSS capacity gaps, the early results on tracking the universities' progress and challenges in strengthening their RMSS and senior researchers' experience with the RMSS assessment process.

METHODS

Partner universities

We worked with four African universities or research institutions which were partners in the Malaria Capacity Development Consortium (MCDC 2008-2015, http://www.mcdconsortium.org/) funded by the Bill and Melinda Gates Foundation and the Wellcome Trust. MCDC supported African scientists to undertake high-quality malaria research and to enhance the operational health research capacity of their home institutions. In particular, MCDC aimed to strengthen the capacity of the African universities to provide academic, administrative and financial support to generate health research of

For peer review only - http://bmjopen.bmj.com/site/about/guidelines.xhtml

international quality despite differences in geography, size and maturity of their research infrastructure.

The institutions were based in Anglophone and Francophone countries in West (2), East (1) and Southern (1) Africa. The entry point for our study into each of the universities was the department (or centre) in which MCDC's collaborating principal investigator was located. These departments had been established between 1957 and 1991; all had active malaria research programmes and offered postgraduate training. At the time of the study the universities had between 6,000 and 60,000 registered students.

Generation of a list of research management and support system components

In order to conduct a holistic assessment of the African universities' health RMSS it was necessary to first create a benchmark by identifying all the components and related best practice required for the optimal functioning of such systems.[19] As no single document available detailed all these components, we drafted an initial list of components by itemising all activities that occur within a project cycle and by identifying all the support mechanisms that are required to conceive, generate and monitor research and to ensure that research findings are used to inform national health policies and practices. The list identified search terms (e.g. research management, research capacity indicators, institutional benchmarking) which guided the collection of relevant information using internet searches. The search for relevant global publications included academic articles, and grey literature such as guidelines and regulations governing research aspects of higher education institutions (Supplementary file Box 1). We also interrogated websites of agencies relevant for each of the themes, and read their reports and documents and any references included therein and consulted with researchers, grants managers and research finance officers within and beyond our own institutions until no new items emerged and saturation was achieved. We aimed to cover aspects of the institutional capacity needed to provide optimal academic, administrative and financial support for operational health research activities from the perspectives of the Dean or Principal of the institution, faculty research support staff and researchers at different career stages.

From the literature (examples in Supplementary file Box 1) we extracted a list of all the items relevant for inclusion in a review of institutional RMSS. To help the development of systematic data collections tools, items on the list were grouped into components which were simultaneously adjusted and expanded to encompass all the aspects of RMSS, with no duplication across components (Supplementary file Box 2). The 'optimal' scenario for an institutional RMSS was therefore derived by amalgamating all the items identified from the literature search and eliminating any redundancy. In order to ensure comprehensiveness and minimise bias, no assumptions were made about what should be included, no selection criteria were applied to the original list of items and they were drawn together under the eight components without losing any of the items. This list of items therefore represented the description of the 'optimal' scenario (i.e. benchmark). The final RMSS components encompassed all the RMSS-relevant items identified in the literature and were:

1. Research Strategies and Policies
2. Institutional Support Services and Infrastructure
3. Supporting Funding Applications
4. Project Management and Control
5. Human Resource Management for Research
6. Human Resource Development for Research
7. External Promotion of Research

For peer review only - http://bmjopen.bmj.com/site/about/guidelines.xhtml

8. National Research Engagement

Development of tools for data collection

The most appropriate methods to be used for collecting data on each of the components and their associated items during subsequent visits to the universities were determined.[26] The primary data collection tool was a guide for semi-structured interviews with different cadres of university staff, supplemented by a list of facilities to be visited at the institutions (i.e. library, IT suite, laboratories) and a list of documents to be reviewed (i.e. strategies, policies, regulations, handbooks).

Inclusion of the entire master list of items for every component in every semi-structured interview would have been impractical and inappropriate. Since each interviewee would have knowledge of specific aspects of RMSS in their institution, combinations of questions were selected from an overall suite (Supplementary file Box 3) to construct focused interview guides for different cadres of interviewees (i.e. Heads of Department/Institute Deans or Principals; senior researchers; staff with research support responsibilities such as administration, finance, human resources, communications, ethics and laboratories). For example, questions for laboratory technicians, but not for other cadres, dealt with equipment maintenance. We ensured that all items from the master list were covered across the set of cadre-specific interview guides.

The data collection tools (lists and interview guides) were reviewed by all members of the research team and adjustments were made to reduce redundancy. Additional changes were made after the first university visit and minor revisions were made during the visit to the second university. After this, no more revisions were required, so this version was used for the two subsequent visits.

Baseline data collection during university visits

Pre-visit briefings were conducted by Skype with the MCDC principal investigator in each of the African universities, to explain the purpose and process of the visits and to schedule interviews with different cadres of staff and students. The principal investigators were provided with the data collection tools in advance of the visits so they were aware of the range and type of information that would be sought. Subsequently, 3-4 day visits to each of the four African universities were conducted by 2-3 members of the research team between September and November 2014.

As far as possible, all data collected during the visits was obtained from at least two independent sources to enhance validity.[27] Interviewees were asked if any aspects of research systems had not been covered by the interview questions and, as a result, procurement procedures were added to the questions for the second and subsequent visits. During each interview, interviewees were asked to propose feasible actions that could be taken to overcome any of the challenges or gaps in research support systems that they mentioned.

Notes from the interviews were typed up within a few hours of each interview, checked against audio-recordings of the interviews (available if interviewees gave permission), and final versions were verified among the site visit team. Information from observation of facilities and review of documents was used to elaborate and verify data from the interviews. A consultation meeting was held at the end of each visit for all available interviewees to share preliminary findings about strengths and gaps identified in the institutional RMSS. In keeping with the principles of inter-disciplinary team reflexivity[28] and of pooling internal and external assessments,[29] we used the meetings to check the accuracy of the findings, to discuss the reasons for discrepancies, to generate

For peer review only - http://bmjopen.bmj.com/site/about/guidelines.xhtml

and prioritise proposed actions, and to ensure that such actions were deemed feasible by institution staff.

Baseline Data analysis

A framework analysis approach was used to manage and analyse the multi-disciplinary information generated from the site visits about institutions' 'baseline' research systems.[30] Data were entered into a matrix which had a row for each of the eight components. Columns for topics within each of the RMSS components that emerged from the interviews were constructed using deductive (i.e. based on the topics/items grouped under each component from the scoping review) and inductive (i.e. unexpected new topics that emerged from the information collected) approaches. Use of the matrix facilitated identification of emerging patterns and comparison of the strengths and weaknesses in each institution's research systems. Following the site visits, findings were presented in a draft report which was reviewed by the MCDC principal investigators in consultation with their institutional colleagues, before being finalised. To respect confidentiality, the final reports were only shared with the MCDC secretariat and the institutions themselves. An anonymised 'overview' report was produced and made publically available which summarised commonalities and differences in RMSS across all institutions and highlighted innovative RMSS practices.[31]

Follow-up interviews for tracking progress and obtaining feedback on the process

Information about progress and challenges in addressing gaps in the institutions' health RMSS was obtained through 2-5 Skype and telephone interviews with the MCDC principal investigators in each institution over fifteen months until May 2016. Each interview lasted 20-40 minutes and covered the gaps and actions identified in the relevant intuitional baseline report. The relevant principal investigator, in discussion with SW and IB, gauged the progress on each action, explained the means by which progress had been achieved, and described any challenges experienced. During the interviews the principal investigators were asked to comment on whether the process had been helpful, and if so how, and which aspects could be improved in the future and to reflect on their role as research manager practitioners.[32] These comments were organized into themes, and quotes reflective of each theme were selected to convey the principal investigators' perspectives in their own words.

Information obtained about progress and challenges around actions in the baseline report were mapped against the eight RMSS components using a pre-prepared matrix and analysed using a framework analysis approach. Two authors (SW, IB) reviewed the self-reported progress of each institution and broadly assessed whether the institutions collectively had made 'good', 'moderate' or 'little/no' progress in addressing the gaps in each component of their research support systems. This helped understanding about which components of research support systems all four universities found most easy to address and which they found hardest. A report outlining progress and challenges was drafted for each institution and reviewed by each principal investigator.

Ethical considerations

This project was considered to be primarily an evaluation which aimed to improve practices for strengthening research capacity so formal ethical approval was not sought. However we explained the study to all participants, we asked each interviewee for their verbal consent to participate and we provided an opportunity for them to refuse without any consequences for themselves.

For peer review only - http://bmjopen.bmj.com/site/about/guidelines.xhtml

RESULTS

Baseline situation

In total 83 interviews were conducted (19-22/university) with eleven different cadres of interviewees (table 1), 65 documents/resources (12-20/university) were reviewed, and facilities observed included libraries, research laboratories and study spaces. The gaps in RMSS that were common (i.e. occurred in at least three of the four universities), and proposed actions that emerged during the on-site visits to address these gaps, were categorised by RMSS component (table 2).

Progress in strengthening universities' RMSS

All of the universities had made some progress in addressing gaps in their research support systems, and there were some common successes and challenges. Examples are provided in(table 3. Although MCDC provided some institutions with limited funding to address some of these gaps, many of the actions, such as re-organisation of management structures or in-house training, did not require additional funds.

Overall, little or no progress was made in Research Policies and Strategies, External Promotion of Research, and National Research Engagement; moderate progress was made in Institutional Support Services and Infrastructure and Human Resource Management and Development for Research; and good progress in Funding Applications and Project Management and Control. Examples of innovative practices and problem-solving were identified for each component (table 3).

The process of assessing and tracking strengthening of RMSS

The process of assessing and providing feedback on institutional RMSS used in the study was universally viewed as a positive and constructive way to raise awareness of the importance of strengthening research support systems and to catalyse broader institutional engagement with these topics. Relevant comments from interviews with the principal investigators included:

"Senior staff are really engaging with this. They understand the importance of the programme"

"The project definitely helped to raise awareness of all the challenges we are facing, that we need more funds and to improve the environment; it highlighted difficulties and that all the partners are now really interested in helping African institutions. It enabled us to start some concrete actions and now we have institutional buy in, now they are engaged and committed to go further"

An area for improvement was in ensuring that important documents provided to institutions, such as drafts of the research capacity assessments, were produced in French as well as English language.

"It would help if the report was in French, with logos, stamp and signature - an official version. Otherwise a translation is not taken seriously"

The comprehensive nature of the assessments and data collection tools provided confidence that all key aspects of research support systems had been covered during the process and helped stakeholders to prioritise and justify their future budgeting and funding requests.

"It was very useful to get an overview of the whole system from an outside team"

"A piecemeal approach would not be effective at all. We need to look at each area. We can then leverage funding ….and use this [assessment] to make sure every area is funded."

For peer review only - http://bmjopen.bmj.com/site/about/guidelines.xhtml

The collaboration between an external team and stakeholders within the institutions brought additional benefits in terms of impartiality and reduction in bias, which would not have been possible with an exclusively internal review team. Seeking opinions from multiple perspectives and the involvement of external team, helped to overcome internal sensitivities.

"It stimulated honest and fair discussion between us all…… It demonstrated our strengths as well as weaknesses. Everyone said it didn't say anything we didn't know but as an outside organisation produced it there were no biases. That's why everyone has agreed we need to move forward"

"Certain areas that the overall report helped when I was presenting the sensitive issues that there are common problems - instead of feeling hopeless, we felt we were doing better in some areas. We knew … that here are political issues. If the recommendation had come from within that could have caused issues"

Addressing gaps in research support systems is a complex undertaking and regular contact with the external team to track progress was helpful for keeping the focus on priorities and maintaining momentum.

"The follow up process was helpful to keep me focused on understanding the changes occurring across the college and in all areas of research management."

"On-going follow up was helpful to keep on track with forward movement"

DISCUSSION

Process and Tools

We have demonstrated that is it possible to construct and implement a coherent, evidence–informed process for assessing and tracking programmes to strengthen institutions' health RMSS. The comprehensive data collection tools drew on current approaches and evidence from several disciplines including research management, education and organisational systems.[33, 34, 35] It has parallels with others' efforts[36] to construct assessment tools to improve the quality of indicators and processes for measuring operational health research capacity strengthening.[20] The assessment process was systematic yet flexible enough to accommodate the complexity and fluidity of health RMSS, across a range of African universities. The assessment process acknowledged the influence of inter-relationships between individual, institutional and wider societal levels on the 'research ecosystem' (i.e. researchers and their institutions, funders and governments who support research, policymakers who use research, and communication specialists who share and discuss the findings with a broad audience).[37] The way in which the assessment process was conducted, particularly the findings from the baseline assessments and the collaborative identification of actions to address health RMSS gaps, was universally viewed as positive, and is consistent with others' experience in reviewing operational health research capacity.[36, 4] In addition the institutional assessments helped to raise awareness of the importance of strengthening RMSS[18] and to catalyse multi-disciplinary engagement in improving RMSS across the institutions.[38]

Such assessments would be difficult for exclusively internal teams to undertake since they may struggle to gain timely access to senior university officials and could be influenced by sensitivities and politics within the institutions. A partnership between senior institutional researchers, who intimately understood the structural, financial and political context, and an external team, who were impartial and experienced in such assessments, was therefore essential to maximise assessment

For peer review only - http://bmjopen.bmj.com/site/about/guidelines.xhtml

validity and contribution to learning.[18] Such insider-outsider assessments have also been used in examining research ethics systems.[29] The transferability of the RMSS assessment tools and processes across geo -political and institutional boundaries means they could be usefully deployed in the increasingly common model of research consortia.[24] Of note is the need to produce reports for non-Anglophone universities in the country's dominant language, since language barriers are known to be a critical handicap in scientific collaborations and in engaging senior university officials.[39]

Tracking Progress/Challenges

Although there are numerous publications of retrospective evaluations of research capacity strengthening efforts, prospective tracking of progress is far less common.[40] We applied an established five step process for assessing baseline status and prospectively tracking changes in operational health research capacity.[18] The researchers perceived the process as constructive since it helped to maintain focus and momentum within the institution, and provided an opportunity to introduce and share innovative approaches to problem-solving at each institution and for each RMSS component. Most institutions had made the best progress in areas that were primarily under the control of the collaborating senior researchers' departments, such as involving finance officers and managers in developing research proposals, and providing training and resources for managing grants. Much of this progress was achieved with limited or no additional funds. This may therefore be a useful indicator of what might be achieved by other research institutions in Africa who have minimal external support.

Gaps in operational health research capacity that were generally found to be most the challenging to remedy depended on university-wide changes. Examples included embedding research training, which was usually non-sustainably linked to projects, within university systems, and ensuring laboratories were accredited and underpinned by sustainable financing models. Most challenging of all were the lack of systems for communication and dissemination of research outputs and for using research to influence health policies and programmes. This lack of institutional knowledge exchange capacity to promote research uptake in Africa has been noted by others.[41]

Limitations of the study

Our study was designed to provide a broad overview of an institution's health RMSS, and therefore could did not explore particular components in depth. Other instruments and guidelines are available to do this including: Good Financial Grants Practice,[42] for researchers' development framework,[34] Octagon for research ethics capacity,[29] 'stepwise' laboratory accreditation,[43] and DRUSSA for research uptake.[44] The MCDC principal investigators varied in their seniority, influence and social capital[45] (i.e. the norms and networks that enable people to act collectively) which may have affected the thoroughness of the assessment phase, as well as the extent of progress especially in implementing university-wide actions. We recognise that the study only included four African institutions and that these cannot be considered representative of the diversity and complexity of universities within the continent and even within individual countries. The lack of a theory of change[46] for the broader MCDC programme meant that explicit articulation of a common set of outcomes and pathway to change for strengthening RMSS was lacking.[47] Tracking information in progress was generally not independently verified, as it was based on Skype or phone interviews with the MCDC principal investigators. The follow up time was 15 months which is too short to be able to demonstrate longer term impact of such a process on health RMSS. Hence, we regard our prospective tracking as an initial experience which could be used to guide a more fulsome, prospective evaluation.

For peer review only - http://bmjopen.bmj.com/site/about/guidelines.xhtml

Contributions to an emerging science

Momentum is gathering around a new global science on research capacity strengthening which draws on implementation research,[48] research evaluation processes[5] and qualitative research methodologies.[49] Our effort is consonant with this developing global science, addressing the area of health RMSS with an explicit and comprehensive set of assessment tools, embedded in a collegial, collaborative process. Similar to a small but growing number of colleagues engaged in contributing to the science-base for research capacity strengthening, we are sharing our tools in a peer-review forum, so that others can apply and adapt them for assessing their own or others' university's RMSS. Linking collaborative RMSS assessments of gaps with collegial generation of actions to address those gaps, and jointly tracking progress on chosen actions and challenges prospectively constitutes a more rigorous approach to operational health research capacity strengthening than has been common to date.[20] In addition, documentation of innovative problem-solving by African institutions is crucial to counter deficit-focused narratives, facilitate sharing among resource-constrained institutions, and facilitate universities' role as agents of change.[50] An additional benefit of using a systematic, common approach to strengthening institutional health research capacity is that it provides evidence for external agencies and governments about better targeting of efforts to make institutions in Africa globally competitive research leaders.

IMPLICATIONS

Research capacity outputs need to be recognised as of equivalent value to research outputs[12] and therefore need a rigorous scientific basis. Our experience in developing and applying an assessment and tracking framework can facilitate similar initiatives in other research oriented institutions in LMICs and their respective consortia. The identification and sharing of RMSS components that are commonly problematic could guide national governments to target their resources towards these weakest components. At the supra-national level, the use of our tools and process, and sharing of the results more widely, enable comparisons to be made across institutions and countries. Such analyses would not only contribute to the science of operational health research capacity strengthening, by enabling common research approaches and tools to be applied in different contexts and by validating findings on common capacity gaps, but also provide guidance to international health and research funders about 'smart' investment of resources. Sharing of problem-solving innovations in RMSS among universities and research institutes with similar resource constraints through such organizations as the African Academy of Sciences is an important more immediate opportunity. Finding ways to share such innovations widely beyond health, for example through inter-disciplinary study tours or joint workshops for researchers and research support staff, is imperative for fostering collaborations for RMSS strengthening, and hence health system strengthening more broadly.

Contributorship statement

SW: Liverpool School of Tropical Medicine, Liverpool, UK (mixed methods researcher) contributed to literature review, study design, baseline and follow up data collection, analysis and final paper

DCC: Professor, Dalla Lana School of Public Health, University of Toronto, Canada (mixed methods researcher)

For peer review only - http://bmjopen.bmj.com/site/about/guidelines.xhtml

contributed to literature review, study design, baseline and follow up data collection, analysis and final paper

OG: Professor, Cheikh Anta Diop University, Dakar, Senegal (MCDC Principal Investigator)
contributed to providing baseline and follow up data and final paper

BTM: Kilimanjaro Clinical Research institute, Kilimanjaro Christian Medical Centre and Kilimanjaro Christian Medical University College Moshi, Tanzania (MCDC Principal Investigator)
contributed to providing baseline and follow up data and final paper

VM: College of Medicine, University of Malawi, Blantyre, Malawi (MCDC Principal Investigator)
contributed to providing baseline and follow up data and final paper

HT: Kwame Nkrumah University of Science and Technology, Kumasi, Ghana (MCDC Principal Investigator)
contributed to providing baseline and follow up data and final paper

IB: Liverpool School of Tropical Medicine, Liverpool, UK (mixed methods researcher)
led study design, contributed to literature review, baseline and follow up data collection, analysis and led drafts of final paper

Acknowledgements
We acknowledge the collaboration of staff of participating universities, which have remained anonymous for reasons of confidentiality of the findings. Thanks to other members of the study and administration team, Ema Kelly and Vicki Doyle of Capacity Development International, Dorte Holler Johansen from the University of Copenhagen, Denmark, and Lorelei Silvester and Denise Wellings from LSTM for their valuable contributions to this study

Data sharing statement
No additional data are available
All data related to this study are included in this submission, either in tables in the manuscript or in supplementary files

For peer review only - http://bmjopen.bmj.com/site/about/guidelines.xhtml

References

1. World Health Organization. Health in 2015: from MDGs, Millennium Development Goals to SDGs. Sustainable Development Goals. Switzerland: World Health Organization. Available: http://apps.who.int/iris/bitstream/10665/200009/1/9789241565110_eng.pdf?ua=1 (accessed 27 February 2017)
2. Koon A, Nambiar D, Rao D. 2012. Embedding of research into decision-making processes. Geneva: Alliance for Health Policy and Systems Research, WHO
3. Bennet S, Akua Agyepong I, Sheikh K, Hanson K, Ssengooba F, Gilson L. 2011. Building the field of health policy and systems research: an agenda for action. *PLoS Medicine* 8: 1023
4. Hawkes S, Aulakh BK, Jadeja N, Jimenez M, Buse K, Anwar I, Barge S, Odubanjo MO, Shukla A, Ghaffar A. 2016. Strengthening capacity to apply health research evidence in policy making: experience from four countries. *Health Policy and Planning* 31(2): 161-70
5. Neufeld V, Cole DC, Boyd A, Njelesani D, Bates I, Hanney SR. 2014. Perspectives on Evaluating Global Health Research for Development: A Background Paper. Taking stock of current practice and ways to improve it. For GHRI-IDRC. 79 pp. p9-11. www.ccghr.ca/wp-content/uploads/2014/07/EGHR4D-final-paper.pdf (accessed 27 February 2017)
6. Newman K. 2014. What is the evidence on the impact of research on international development. London: DFID. https://assets.publishing.service.gov.uk/media/57a089aced915d622c000343/impact-of-research-on-international-development.pdf (accessed 27 February 2017)
7. NEPAD Planning and Coordinating Agency (NPCA) 2014. African Innovation Outlook 2014, NPCA, Pretoria. http://www.un.org/africarenewal/sites/www.un.org.africarenewal/files/AIO_2_Final%20Product[2].pdf (accessed 27 February 2017)
8. Neupane B. 2015. A more developmental approach to science. UNESCO science report: towards 2030 http://en.unesco.org/unesco_science_report (accessed 27 February 2017)
9. United Nations Educational, Scientific and Cultural Organisation (UNESCO) Institute for Statistics. Number of Researchers per million inhabitants by country. <http://www.uis.unesco.org/ev.php?URL_ID=3755&URL_DO=DO_TOPIC&URL_SECTION=201>. converted by ChartsBin statistics collector team 2011, Number of Researchers per million inhabitants by Country, ChartsBin.com, <http://chartsbin.com/view/1124>.
10. Vogel I. 2011. Research Capacity Strengthening: Learning from Experience. UKCDS, London. http://www.ukcds.org.uk/sites/default/files/content/resources/UKCDS_Capacity_Building_Report_July_2012.pdf (accessed 27 February 2017)
11. Nuyens Y. 2007. 10 best resources for health research capacity strengthening. *Health Policy and Planning* 22(4): 274-276
12. Franzen SRP, Chandler C and Lang T. Health research capacity development in low- and middle-income countries: reality or rhetoric? A systematic meta-narrative review of the qualitative literature. *BMJ Open* 2017;7:e012332
13. Sawyerr A. 2004 African universities and the challenge of research capacity development. *JHEA/RESA* 2(1): 213–42.
14. Miiro GM, Oukem-Boyer OOM, Sarr O, Rahmani M, Ntoumi F, Dheda K, Pym A, Mboup S, Kaleebu P. 2013. EDCTP regional networks of excellence: initial merits for planned clinical trials in Africa. *BMC Public Health* 13: 1

For peer review only - http://bmjopen.bmj.com/site/about/guidelines.xhtml

15. Cole DC, Johnson N, Mejia R, Mccullough H, Turcotte-Tremblay A-M, Barnoya J, Falabella Luco S. 2016. Mentoring health researchers globally: Diverse experiences, programmes, challenges and responses. Global Public Health 11(9): 1093-108
16. Enoch J. 2015a. Rapid mapping of international funders' research capacity strengthening priorities. UKCDS. http://www.ukcds.org.uk/sites/default/files/content/resources/UKCDS%20rapid%20RCS%20mapping%20for%20DFID.pdf (accessed 27 February 2017)
17. Enoch J. 2015b. Health Research Capacity Strengthening: A UKCDS Mapping. Published by UKCDS. http://www.ukcds.org.uk/our-work/health-research-capacity-strengthening-a-ukcds-mapping (accessed 27 February 2017)
18. Bates I, Boyd A, Aslanyan G, Cole DC. 2015a. Tackling the tensions in evaluating capacity strengthening for health research in low-and middle-income countries. Health Policy and Planning 30: 334-344.
19. Bates I, Boyd A, Smith H, Cole DC. 2014. A practical and systematic approach to organisational capacity strengthening for research in the health sector in Africa. *Health Research Policy and Systems* 12: 11
20. Cole D C, Boyd A, Aslanyan G, Bates I. 2014. Indicators for tracking programmes to strengthen health research capacity in lower-and middle-income countries: a qualitative synthesis. *Health Research Policy and Systems* 12: 17
21. Department for International Development (DFID) 2008. Research strategy 2008-2013 Working Paper Series: Capacity Building. https://assets.publishing.service.gov.uk/media/57a08b8ee5274a31e0000c0a/ResearchStrategyWorkingPaperfinal_capacity_P1.pdf ((accessed 27 February 2017)
22. Ghaffar DA, Ijsselmuiden C, Zicker F. 2008. Changing mindsets: Research capacity strengthening in low-and middle-income countries, Council on Health Research for Development (COHRED). http://www.who.int/tdr/publications/documents/changing_mindsets.pdf (accessed 27 February 2017)
23. ESSENCE (Revision 2016). Planning, Monitoring and Evaluation. Framework for Capacity Strengthening in Health Research. WHO. www.who.int/tdr/publications/Essence_frwk_2016_web.pdf (accessed 27 February 2017)
24. Kirkland J, Bjarnason S, Stackhouse J, Day R. 2006. International research management: Benchmarking programme. Report to the HEFCE by the Association of Commonwealth Universities.
25. Altbach PG, Salmi J. 2012. *The road to academic excellence: the making of world-class research universities*. Directions in development; human development. Washington DC: World Bank
26. Denzin NK, Lincoln YS. 2008. Collecting and interpreting qualitative materials. Published by Sage.
27. Golafshani N. 2003 Understanding reliability and validity in qualitative research. *The Qualitative Report* 8: 597-606
28. Cohen D, Crabtree B. Reflexivity. Qualitative Research Guidelines Project. http://www.qualres.org/HomeRefl-3703.html (accessed 27 February 2017)
29. Hyder AA, Zafar W, Ali J, Ssekubugu R, Ndebele P, Kass N. 2013 Evaluating institutional capacity for research ethics in Africa: a case study from Botswana. *BMC Medical Ethics* 14: 31
30. Gale NK, Heath G, Cameron E, Rashid S, Redwood S. 2013. Using the framework method for the analysis of qualitative data in multi-disciplinary health research. BMC Medical Research Methodology 13: 117

For peer review only - http://bmjopen.bmj.com/site/about/guidelines.xhtml

31. Bates I, Cole DC, Doyle V, Kelly E, Holler Johansen D. 2015b. Strengthening Research Management and Support Systems (RMSS) in African Universities. Overview Report for Four Universities to MCDC. September - October 2014. Accessed 27 February 2017 at http://www.mcdconsortium.org/sites/www.mcdconsortium.org/files/MCDC%20RMSS%20overview%20report%20FINAL%209jan15_0.pdf
32. Cunliffe AL. 2004. On becoming a critically reflexive practitioner. *Journal of Management Education* 28(4): 407-426
33. Quality Assurance Agency 2010. Code of practice for the assurance of academic quality and standards in higher education. http://www.bbk.ac.uk/linkinglondon/resources/apel-credit-resources/pub_Nov2010_QAA_COP_section_2_HE_guidance.pdf (accessed 27 February 2017)
34. Vitae, 2010. Research Development Framework. https://www.vitae.ac.uk/vitae-publications/rdf-related/researcher-development-framework-rdf-vitae.pdf/view (accessed 27 February 2017)
35. UK Research Councils, Arts and Humanities Research Board Joint statement of the Research Councils'/AHRB's skills training requirements for research students. http://www.rcuk.ac.uk/documents/skills/statementofexpectation-pdf/ (accessed 27 February 2017)
36. Jessani N, Lewy D, Ekirapa-Kiracho E, Bennett S. 2014. Institutional capacity for health systems research in East and Central African schools of public health: experiences with a capacity assessment tool. *Health Research Policy and Systems* 12: 21
37. Wellcome Trust. undated. Strengthening research ecosystems in Africa and Asia. https://wellcome.ac.uk/what-we-do/our-work/research-ecosystems-africa-and-asia. (accessed 27 February 2017)
38. Mashaah T, Hakim J, Chidzonga M, Kangwende RA, Naik Y, Federspiel N, Fiorillo S, Scott J, Gomo E. 2014. Strengthening research governance for sustainable research: Experiences from three Zimbabwean universities. *Academic Medicine* 89: S69-S72
39. Hwang K. 2013. Effects of the language barrier on processes and performance of international scientific collaboration, collaborators' participation, organizational integrity, and inter-organizational relationships. *Science Communication* 35(1): 3–31
40. Wirrman-Gadsby E. 2011. Research capacity strengthening, donor approaches to improving and assessing its impact in low- and middle-income countries. *International Journal of Health Planning and Management* 26: 89–106.
41. Ayah R, Jessani N, Mafuta EM. 2014. Institutional capacity for health systems research in East and Central African schools of public health: knowledge translation and effective communication. *Health Research Policy and Systems* 12: 20
42. African Academy of Sciences. Undated. Good Financial Grants Practice – Standardising, simplifying and strengthening governance of grant funding. http://aasciences.ac.ke/aas/en/academy/academy-pages/good-financial-grants-practice/ (accessed 27 February 2017)
43. World Health Organization Regional Office for Africa. Undated. WHO Guide for the Stepwise Laboratory Improvement Process Towards Accreditation in the African Region (with checklist). http://www.who.int/tb/laboratory/afro-slipta-checklist-guidance.pdf (accessed 27 February 2017)
44. Development Research Uptake in Sub-Saharan Africa (DRUSSA). Undated. Strategies for effective research uptake communication and impact. http://www.drussa.net/index.php?option=com_content&view=article&id=2788%3Astrategies-

For peer review only - http://bmjopen.bmj.com/site/about/guidelines.xhtml

for-effective-research-uptake-communication-and-impact&catid=211%3Atools-a-tips-open&Itemid=297&lang=en (accessed 27 February 2017)

45. Woolcock M, Narayan D. 2000. Social capital: Implications for development theory, research, and policy. *The World Bank Research Observer* 15: 225-249
46. Vogel I. 2012. Review of the use of 'Theory of Change' in international development. Report for the UK Department for International Development (DFID). August. https://assets.publishing.service.gov.uk/media/57a08a5ded915d3cfd00071a/DFID_ToC_Review_VogelV7.pdf (accessed 27 February 2017)
47. Boyd A, Cole DC, Cho DB, Aslanyan G, Bates I. 2013 Frameworks for evaluating health research capacity strengthening: a qualitative study. *Health Research Policy and Systems* 11(1): 46.
48. Peters DH, Adam T, Alonge O, Agyepong IA, Tran N. 2013. Implementation research: what it is and how to do it. *BMJ* 347: f6753
49. Creswell JW, Plano Clark VL. 2010. 2nd edition. Designing and Conducting Mixed Methods Research. Published by Sage https://us.sagepub.com/en-us/nam/designing-and-conducting-mixed-methods-research/book233508
50. Hansen JA, Lehmann M. 2006. Agents of change: universities as development hubs. *Journal of Cleaner Production* 14: 820-829

For peer review only - http://bmjopen.bmj.com/site/about/guidelines.xhtml

Table 1. Number of interviewees for baseline data collection by cadre and institution

	Institution number			
	**1**	**2**	**3**	**4**
Principal investigator	1	1	1	1
PhD students	5	1	3	0
Senior lecturer/lecturer/post-doc	3	1	2	2
Dean/registrar/ provost/principal	4	4	3	5
Head of Department	1	3	1	1
Research/ethics manager/administrator	2	4	1	2
Human resources staff	1	1	0	2
Finance/procurement staff	2	2	2	1
IT/library staff	3	3	4	4
Laboratory staff	0	2	2	1
Communications staff	0	0	1	0
**Total**	**22**	**22**	**20**	**19**

For peer review only

For peer review only - http://bmjopen.bmj.com/site/about/guidelines.xhtml

Table 2. Consolidated key gaps in research management and support systems (RMSS) and proposed actions, by RMSS component

Gaps	Proposed actions
**Research Strategies and Policies**	
• No research strategy or not available or publicised<br>• Lack of central tracking of research activities	• Departments/universities need an accessible research strategy with polices and guidelines to support its implementation<br>• Electronic research management support systems are needed to track proposals and projects and to document research income and disbursement including overheads
**Institutional Support Services and Infrastructure**	
• Lack of research support offices and/or insufficient coordination between departments and university levels<br>• Inadequate resourcing and lack of clarity about the role and long term financial sustainability of research support offices<br>• Research laboratory facilities are not accredited and lack over-arching planning to harmonise equipment purchase and maintenance across multiple short term projects<br>• Unclear relationship between hard copy library facilities and increasing use of e-resources	• The roles and relationships between university level research coordination and research support offices at Faculty or College level need to be clarified<br>• The strategy for research support offices at Faculty or College level needs to be clarified and mechanisms found for long term sustainability and buy-in by the researchers<br>• Achieve international laboratory accreditation for the institution's own laboratories; harmonise research laboratories' activities with those of affiliated organisations and establish clear processes and costs for researchers wishing to access these facilities<br>• Pro-actively plan the future of book libraries in the context of the shift to increasing use of e-resources, including their possible integration with ICT facilities
**Supporting Funding Applications**	
• Insufficient quality assurance checks and signing off processes for proposal submissions or contracts which could put the institution at risk of contractual or intellectual property issues	• Set up mechanisms for timely, multi-disciplinary (e.g. finance, legal, ICT, laboratory, library, procurement) input into proposal development<br>• Set up a formal process for quality assurance and authorisation of proposals before submission and for tracking the outcome of submissions
**Project Management and Control**	
• Senior researchers spend a substantial proportion of time on administrative, procurement and	• Establish an electronic research information system to systematically manage and track all aspects each project including the project agreement, protocol,

For peer review only - http://bmjopen.bmj.com/site/about/guidelines.xhtml

other issues that could be more effectively taken on by non-academic professional staff<br>• Lack of systems for tracking financial spend against budget for projects risks under- or over-spend<br>• Unclear lines of responsibility between researchers and finance officers regarding financial tracking and reporting	budgets, funding requirements, accounting and audit, and to maximise recoupment of overheads<br>• Establish a formal project approval process for successful applications, including and contract review and sign off<br>• Encourage researchers to include and budget for experienced administrators to help reduce the time they spend on project administration and to actively include other relevant inputs such as procurement expertise<br>• Provide joint training in financial management for researchers and finance officers and increase clarity and understanding about their various roles and responsibilities in relation to each other, the institution and the research funders
**Human Resource Management for Research**	
• Lack of clarity on contractual arrangements, and therefore institutional responsibility, for short-term project staff<br>• Poorly defined, or non-existent, career paths for non-academic professionals such as ICT, library and administrative staff<br>• No formal post-doctoral career posts for researchers	• Strengthen HR skills and structures so that they can better support researchers and research projects, and to ensure that project staff are university employees with access to the protection and facilities of the institution where this is not currently the case<br>• Formalise career tracks for research support staff<br>• Formal post-doctoral training programmes need to be established to develop and retain talented researchers
**Human Resource Development for Research**	
• No coordinated, institutionalised programmes for induction or research skills training for researchers; reliance on projects to provide training means focus is on technical skills rather than generic skills, such as leadership and research communication<br>• Training offered at university level (e.g. computer skills, literature searching) poorly publicised and utilised by researchers	• Provide a formal induction programme and training needs assessment for new research staff<br>• Establish an institutional programme of skills training for researchers, possibly through a dedicated unit, that includes non-technical skills such as leadership, supervision and project management<br>• Improve incorporation of existing training opportunities (e.g. provided by library and ICT staff) into a core skills training programme for researchers
**External Promotion of Research**	
• Promotion of research activities and successes by the universities not prioritised although widely recognised as important	• Review research section of universities' website to ensure information is current and that hyperlinks are working<br>• Consider setting up a unit specifically to enhance the visibility of institutional and/or departmental

For peer review only - http://bmjopen.bmj.com/site/about/guidelines.xhtml

	research activities and outputs<br>• Provide training in research communication to improve researchers' ability to write "jargon-free" communications such as press releases and policy briefs
**National Research Engagement**	
• Insufficient publicising of institutional research outputs in influencing national and international policy making and programming	• Explore options for improving researchers' ability to impact on national health research priorities and practices<br>• Universities and departments should systematically document and showcase national and international uptake and utilisation of the research findings they have generated

For peer review only

For peer review only - http://bmjopen.bmj.com/site/about/guidelines.xhtml

Table 3. Examples of progress on actions, challenges and innovative problem-solving during 2015-16, by RMSS* component and institution

Compo-nent [Overall progress]	Institution 1	Institution 2	Institution 3	Institution 4	Examples of innovative practices and problem-solving
**Research Strategies and Policies [little/no progress]**	University level strategy being approved but College level not yet developed.<br>A new university-wide research database now in place. A small university research fund has been set up.	Consultation on the strategy has taken place. A draft document is being written to submit for management approval.<br>New research management software has been installed to manage the research database	A research grant office is planned for early 2016 and a research database will be created within this office.	Strategy is on-hold as wide-scale institutional constitutional change is underway.<br>[post-project note: *Strategic plan 2017-2022 was approved December 2016*]	• Through a consultative process and workshop involving researchers, university officials, research support staff and representatives from external national agencies, the Ministry of Health and international research consortia, a research strategy was agreed based on a SWOT analysis
**Institutional Support Services and Infrastruc-ture [moderate progress]**	Work is on-going to coordinate inputs to proposals and grant management by PIs and finance staff. Workshops took place in 2015.	The first MoUs and SoPs are in development for grant related activities.<br>ICT policies for marketing and staff engagement are being developed.<br>Sustainability planning for lab resources and budgeting is underway.	A new high speed internet (DSL) has been installed at the Department of Biology, the PhD dedicated space and the Faculty library.<br>Steps towards formal accreditation of quality assurance	Progress has been made in establishing a research support office (grants unit) with administrators and financial staff appointed.	• A Laboratory Committee has been formed and inventoried all key laboratory resources within the College and affiliated research institutions

For peer review only - http://bmjopen.bmj.com/site/about/guidelines.xhtml

			processes and certification for the department labs have begun.		
**Supporting Funding Applica-tions [good progress]**	A university-wide database has been implemented.<br><br>The finance office is now involved in proposal development and joint training has taken place.	A new system for disseminating information about the research support services has been instigated.<br><br>"How-to" guideline on developing research proposals with a budget framework and checklists has been launched.<br><br>A research careers development webpage has been developed by the institution with materials for fledgling researchers	Implementation of a new research grant office and recruitment of a research coordinator has been agreed.	Administrators from the research/grants office have started assisting the PIs in proposal development, registering projects and tracking implementation and management.<br><br>The Grant office has been newly registered with several "calls application portals" and a database of researcher interests is being created.	• Production of a new "how-to" flow chart outlining the steps needed to develop research proposals<br>• A framework for calculating a proportion of staff time to be included in proposals for grants management has been established<br>• Topic-specific research groups have been formed which can respond to funding calls and collaboration opportunities<br>• A legal unit has been set up to guide the contract review process and a contract template and checklist developed
**Project Manage-ment and Control [good progress]**	Contract review, sign off and risk management procedures have been strengthened with contracts signed off by Provosts or Deans of	New systems for pre- and post-award management have been developed.<br><br>A new policy for compulsory post-award	SOPs for expenses and account management have been developed and validated with PIs, finance and	Project management procedures are now overseen by an administrator who manages the grant and liaises with the PI on the	• Standard operating procedures for expenses and account management have been developed jointly by researchers, finance and administrative

For peer review only - http://bmjopen.bmj.com/site/about/guidelines.xhtml

	Colleges.<br><br>Need for in-house training for admin to support PIs identified.	"Grants Management" inductions for all successful Principal Investigators is available.<br><br>A grants administrator has now been appointed. A new legal unit has been set up as part of research support services	administrative officers	implementation of the project.<br><br>A new financial management system PASTEL is now being used but staff need training to upload project data.	officers.<br>• New quarterly meetings between finance officers and researchers are used to address financial management of project grants
**Human Resource Manage-ment for Research [moderate progress]**	HR recruitment policies and procedures for project staff have been reviewed and developed.<br><br>A new staff development programme has been launched.<br><br>On-going meetings are continuing to discuss the need to develop a post-graduate program	A new training needs assessment for research support staff has been completed and training plans developed.<br><br>Procedures for hiring post- doctoral fellows have been established and terms and conditions of service defined.	The introduction of project management training courses and cross site visits for administrative staff and early career researchers have strengthened human resource management.<br><br>Staff needs for personal development have been identified but there is yet to be a plan to address these.	A team has been formed to develop a research scale based on a national scale for researchers.<br><br>Post- doctoral programmes are not yet institutionalised	• Career Development Centres have been established and stocked with training resources<br>• Human resources staff are being included in planning for research staff development
**Human Resource Develop-ment for Research**	Work is still on-going to plan a formal research-based induction process for project staff and	Work has begun towards a comprehensive competency- based capacity building programme for junior and	Progress made towards adaptation and institutionalisation of the doctoral research	The role of HR teams in research staff development has been reviewed and defined.	• An annual research training calendar has been developed for research staff based on a formal training needs assessment

For peer review only - http://bmjopen.bmj.com/site/about/guidelines.xhtml

**[moderate progress]**	fledgling researchers.<br>Mentorship training and pairing has taken place.	senior researchers.<br>A research capacity gap validation workshop and a training needs assessment for research staff have been conducted. A new annual research training calendar has been developed.	courses for researchers.<br>An external funder is working with the institution to plan personal development and leadership courses.	More work needs to be done to build a structured programme of capacity development for new and existing staff in research skills	• Regular training is now provided by the university on the roles and responsibilities of research leaders, administrators and finance staff throughout the grant life cycle
**External Promotion of Research [little/no progress]**	Limited training and mentoring in research communication provided by an external agency has started.<br>Some improvement of the research projects on the College website but more needs to be done to encourage researchers to add their details.<br>The academic board are to incorporate research uptake and the management of research outputs, into the university's centralised research	Research profiles of faculty are being introduced onto their webpages.<br>A separate research office website is under construction.<br>There are plans for a Knowledge Management Unit to help achieve better visibility of research activities.	The Faculty of Medicine website has been updated with current research projects.<br>The recruitment of a communication officer is planned, who will be responsible for supporting the publicising and dissemination of research activities and uptake.	The institution has an ongoing strong external collaborative network including an annual PhD symposium.<br>The planned new research strategy will embed research dissemination and uptake as a high priority area.<br>Work is ongoing to restructure the website	• An attractive annual university research report which chronicles recent research activities and highlights individuals, departments and colleges has been produced and is publically available

For peer review only - http://bmjopen.bmj.com/site/about/guidelines.xhtml

	policy.				
**National Research Engage-ment [little/no progress]**	Mechanisms still need to be established at College and departmental level to promote, monitor and record communication and to share opportunities for engagement between projects.<br><br>Continuation of institution's work with multi-national partners on national research engagement to feed into the College's research strategy.	Key national health policy making forums have been targeted with specifically-designed materials.<br><br>A local travel grant system is being established to support researchers to attend national meetings.<br><br>A template has been developed for annual research reports, to highlight successes, failures and opportunities for policy engagement	Ongoing participation in national tailored monitoring and evaluation training courses for medical district officers and health supervisors where research results and experience will be shared	Researchers continue to attend a national research conference (e.g. in 2015) to disseminate their projects.<br><br>Promotion of their research into policy and practice is not yet stated in the research strategy as a core element of a researchers' responsibility	• A university research uptake strategy has been published<br>• University regularly hosts a meeting of leading thinkers in science, policy, industry and civil society in Africa

*RMSS = research management and support system

For peer review only - http://bmjopen.bmj.com/site/about/guidelines.xhtml

For peer review only

Supplementary Data

Box 1 Key sources used to inform review tools

Optimal institutional research management and support

- Ajai-Ajagbe, P. (2013) #13 Research Management in African Universities. *IAU Horizons* (June) Vol. 19 N° 2 pp 32-33. http://www.acup.cat/sites/default/files/iauhorizonsvol192en1.pdf
- Bates I, *et al*. 2011. Assessing and Strengthening African Universities' Capacity for Doctoral Programmes. *PLoS Medicine.* 8(9).
- Cole DC, Boyd A, Aslanyan G, Bates, I. 2014. Indicators for tracking programmes to strengthen health research capacity in lower- and middle- income countries: a qualitative synthesis. *Health Research Policy and Systems.* **12(17).**
- Dean O, Smith (2011). Managing the Research University. Oxford University Press. ISBN 978-0199793259. Retrieved 2014-08-11.
- Dyason K, Harle J. Research project management in African universities. RIMI4AC. Available online at: http://www.rimi4ac.net/site_media/CONTENT_uploads/ProjectManagement_v1%20[SINGLE%20PAGES].pdf (accessed on 13/08/2014).
- ESSENCE, 2011. Planning, Monitoring and Evaluation. Framework for Capacity Strengthening in Health Research (pdf). TDR/WHO: Geneva. Available online at: http://whqlibdoc.who.int/hq/2011/tdr_essence_11.1_eng.pdf (accessed on 16/05/2014).
- ESSENCE, 2012. Five keys to improving research costing in low- and middle- income countries (pdf). TDR/WHO: Geneva. Available online at: http://whqlibdoc.who.int/hq/2012/TDR_ESSENCE_1.12_eng.pdf?ua=1 (accessed on 16/05/2014).
- Green J, D Langley (2009) Professionalising research management Bristol: HEFCE. A report to HEFCE
- HEFCE (2008) Institutional Benchmarking Template: Establishing and meeting the leadership and development needs of principal investigators
- Kirkland J, Bjarnason S, Stackhouse J. 2006 International research management: benchmarking programme . Report to HEFCE by the Association of Commonwealth Universities. Available online at: http://webarchive.nationalarchives.gov.uk/20100202100434/http://www.hefce.ac.uk/pubs/rdreports/2006//rd11_06/rd11_06.pdf (accessed on 13/08/14)
- Kirkland J, Ajai-Ajagbe, P. 2013. Research management in African universities: from awareness raising to developing structures**.** The association of commonwealth universities (ACU). Available online at: http://carnegie.org/fileadmin/Media/Publications/PDF/ACU_-Research_Management_in_African_Universities_Report_-_2013.pdf (accessed on 02/08/2014, no longer on-line).

For peer review only

- Mirzoev T, Lê G, Green A, Orgill M, Komba A, Esena R, Nyapada L, Uzochukwu B, Made W, Nxumalo N, Gilson L. 2013 Assessment of capacity for Health Policy and Systems Research and Analysis in seven African universities: results from the CHEPSAA project. *Health Policy and Planning*. 10.1093/heapol/czt065
- QAA. 2012. UK Quality Code for Higher Education (pdf). QAA: Gloucester. Available online at: http://www.qaa.ac.uk/Publications/InformationAndGuidance/Documents/B10.pdf (accessed on 16/05/2014).

Cross institution/Country wide research management

- Meek & Olson. Effectiveness of Research and Innovation Management at Policy and Institutional Levels. OECD. (undated) http://www.oecd.org/sti/Effectiveness%20of%20research%20and%20innovation%20management%20at%20policy%20and%20institutional%20levels_Meek%20and%20Olsson.pdf

The structure of the observation checklists, interview guides and the phrasing of some of the questions were based on previously designed evaluation tools, specifically those from the following references:

- Benchmarking Southern African Universities 2014 DRAFT Summary Association of Commonwealth Universities. Available online at: https://www.acu.ac.uk/focus-areas/benchmarking-african-universities (accessed on 13/08/14)
- Kirkland J, Bjarnason S, Stackhouse J. 2006 International research management: benchmarking programme . Report to HEFCE by the Association of Commonwealth Universities. Available online at: http://webarchive.nationalarchives.gov.uk/20100202100434/http://www.hefce.ac.uk/pubs/rdreports/2006//rd11_06/rd11_06.pdf (accessed on 13/08/14)
- Unpublished documents (2014) from RS-DFID Africa Capacity Building Initiative Evaluation: Toolkit (Draft)
- Unpublished documents (2013) from the Royal Society- Leverhulme African Award Evaluation: interview guides, survey methodology, findings report.
- Unpublished documents (2013) from the HRCSI Evaluation 2013: Interview guide package.
- Unpublished documents (2012) from Enhancing Laboratory Capacity Strengthening for NTD control, Deliverable One: tool for capacity gap analysis, interview guides.

For peer review only

Box 2. List of topics/items relevant to university research management and support systems (RMSS), grouped by components

University Research Strategy
The university has a research strategy
The research strategy is framed within the overall goals of the institution. The strategy is distinct from but links clearly with, and is complimentary to, other institutional plans, strategies and policies
The research strategy explicitly states its purpose to assist the business of the institution, identifies priorities, and monitor progress
The institution's mechanism for determining research strategy is transparent and widely owned
The institutional research strategy fully involves faculties in its design and implementation, and policies carried out by individual schools or departments are consistent with it
Implementation of the research strategy is overseen by an appropriate member of senior management. The strategy is also backed up by appropriate manpower & resources, to make sure it is implemented
The research strategy has the facility to draw on a range of evaluation mechanisms which might include sources external to the university - such as external peer review including other universities
The Research Management Office [if it exists] is fully involved in the drafting of institutional research strategies in conjunction with other appropriate offices
The research strategy is underpinned by the internal funding mechanisms for research
The research strategy is, as far as possible, responsive to the research funding environment and opportunities (at national, international and regional levels)
The research strategy seeks to add value to existing activity by proactively highlighting new opportunities for internal and external collaboration. The strategy should also promote interdisciplinary research and the development of early career researchers
The research strategy is effectively communicated, monitored, reviewed and developed/refined
Methods for evaluation of the strategy and performance indicators should be established from the outset. Key performance indicators should include a balance of quantitative and qualitative methods
The research strategy should be sufficiently flexible and defined within a reasonable time frame (e.g. 5 years) reviewed regularly, and be capable of evolving in response to events
The strategy should take into account the need for appropriate staff incentives
**Institutional Research Capacity**

For peer review only

The institution has a unit dedicated to research management (Research office)<br>The institution has a research committee to develop strategies to assist the University in meeting its research objectives, identify priorities, advise on stakeholder engagement, monitor research performance, discuss annual department updates, monitor national and international research policy which effect the institution<br>The research unit has an adequate number of Staff to fulfil the needs of researchers<br>Culture where research is valued, accepted, encouraged and enjoyed<br>Ethics committee exists to ensure research conducted is ethical<br>Evidence of accessible guidance to help researchers through the research process including governance and ethics<br>Clear academic honesty guidelines in place<br>Able to meet requirements of GCP and GLP<br>Sufficient facilities are available for research (studying, IT, library, technology, laboratories etc)
**Supporting funding applications**
Institutions have regular, effective and proactive means of informing academics staff on funding opportunities and the strategic directions of funding agencies where possible, these should involve direct communication between the Research Office and individual staff<br>Central research Offices have developed and strategically use key contacts in faculties schools, institutes and departments to facilitate a two way flow of information in funding opportunities and research interest<br>The institution maintains a searchable database on institutions research performance, capabilities and contact, including all past projects and proposals<br>information and current policy from all funders is maintained and communicated as appropriate<br>The Research Office holds regular information and updating sessions and targeted workshops for faculty members and graduate students with the purpose of providing information on funding opportunities, proposal development and the development of collaborative research teams to respond to one-off as well as on-going research opportunities<br>The institution seeks to establish an effective two-way communication strategy between themselves and major sponsors and proactively seek to develop that relationship<br>The institution has clear mechanisms in place to handle internal external enquiries regarding possible research and consultancy opportunities and to monitor the outcomes of these on a regular basis<br>The Research Office actively encourages collaboration between different departments within the institution including senior Academic Office, Public Relations, Marketing and Registry

For peer review only

The institution seeks to develop mechanisms to effectively track and involve alumni working in key positions with current, past and potential sponsors and in government<br><br>The research office actively brings key staff together in response to large scale tender and proposals proposal requests where appropriate and where consistent with research strategy<br><br>Institutions through the Research Office or other appropriate office, ensure that proposals are reviewed by experienced academic and research staff (externally, where appropriate) prior to submission<br><br>Proposals are only submitted with clear support from Head of Department of other appropriate management authority. Key personnel who need to be aware' of the project should be notified.<br><br>The institution approves all proposals before submission and research offices maintain records on the progress of all proposals<br><br>The information gained from previously submitted proposals is used to inform future proposals<br><br>The institution has a clear transparent and widely disseminated formula for determining the full economic cost of any give project, including indirect costs and staff time<br><br>full costing is calculated for each externally funded project even if this is not reflected in the price charged<br><br>All proposed research should be consistent with the institutions overall research strategy<br><br>The institutions provides clear guidance to staff and external sponsors as to which kind of projects and contractual terms are acceptable<br><br>The institution has clear risk assessment procedures for proposed projects which recognise the need to involve several key offices within the institution<br><br>The institution systematically reflects on its progress against its research strategy including regular comparisons with other institutions of similar nature
**Project Management and control**
All project proposals contain explicit statements of how the project will be managed and, where possible and appropriate, provision for the appointment of specialist staff<br><br>Mechanisms are in place to recognise the critical role of Principal Investigators, to ensure that they and other key actors are aware of their roles and responsibilities before commencement of the project and where required, that appropriate training is undertaken.<br><br>Key milestones (including reporting and financial review dates) are agreed with key actors at the outset and updated amongst all those actors throughout<br><br>Key actors, including Principal Investigators and Deans, are provided with regular and up to date project information (including financial, human resources, IP, and commercialization information), through on-line access or regular statements

For peer review only

Information provided to key actors, including Research Officers and Deans, pro-actively highlights any risks and obligations specific to both them and the institution.<br><br>Procedures are in place to ensure that all those with access to research are covered by appropriate confidentiality and rights assignment agreements (depending on jurisdiction), particularly those who are covered by a contract of employment with the institution<br><br>Appropriate data management policies exist (covering ethical and legal compliance, copyright and IPR issues, data storage, security, sharing and retention)<br><br>Appropriate health and safety policies are in place (encompassing staff induction, safety officers, evacuation procedures etc)<br><br>Appropriate insurance arrangements are in place for both staff and clinical trials (if applicable)<br><br>Mechanisms are in place to ensure that intellectual property both brought to and emerging from research is identified, protected, tracked and signed off at all stages and that staff have access to specialist advice in this regard.<br><br>Procedures are in place for the appropriate monitoring of material transfer agreements.<br><br>Mechanisms are in place to identify possible delays and monitor expenditure to ensure it is in line with project budgets<br><br>The institutions has an explicit consistent framework within which academic units can predict future revenue and expenditure, especially where such income contributes to underpinning core activities<br><br>Mechanisms are in place for the disclosure and management of conflicts of interest.<br><br>Mechanisms are in place to obtain feedback project sponsors, which can be taken into account in future planning<br><br>Formal closure and continuous monitoring processes are in place ensuring that all obligations have been and continue to be met and that opportunities arising from the project are identified.
**Training and staff development for research**
Evidence of research training needs assessments<br><br>Provision of research skills training shaped around skills background and needs of different professional groups<br><br>There is availability and use of funds for research skills training for research management staff, researchers and academic staff<br><br>There is availability of a range of research skills training for students, research management staff and researchers covering-

For peer review only

Proposal writing, grant application, data analysis and management (including software and qualitative analysis) ethics, health and safety, GCP and GLP, generic research skills (quantitative and qualitative) academic writing and publishing<br><br>Evidence of matching novice and experienced researchers<br><br>Mentorship and supervision structures for students and early career stage researchers and new PI's<br><br>Individual job descriptions support research/institutional objectives<br><br>Policies are in place to support recruitment and contract negotiation for new support staff<br><br>Staff skills and abilities are matched to research needs<br><br>Seminar programmes relating to research undertaken<br><br>The research management structure and policies form a core element of induction programmes for new academic and technical staff as well as new postgraduate students.<br><br>Research strategy, policy and management issues form a core element of ongoing professional development programmes for mid-career and senior academic staff.<br><br>Staff in leadership roles (e.g. Deans) are offered appropriate instruction in research strategy, policy and management, as well as being involved in discussion of good practice within the institution<br><br>The Research Office maintains effective ongoing relationships with internal clients at all levels (faculty, department, individuals) with a view to supporting research staff and understanding their needs.<br><br>Performance measures for research management are established and are widely available/disseminated.<br><br>The institution makes provision for appropriate incentives to enhance the research activity of new and emerging researchers. Such incentives might include conference grants and other start-up funding.<br><br>Policies for providing incentives for staff research activity are transparent, easy to understand and consistent across the institution.<br><br>Career development opportunities<br><br>Career pathways exist for researchers
**Teaching capacity to support research**
Number of (half as a minimum) full-time academic staff as active and recognised contributors to subject associations, learned societies and relevant professional bodies.<br><br>Number of (third as a minimum) academic staff with recent (i.e. within the past three years) personal experience of research activity (including external examination, review panels, collaborative research)

For peer review only

Number of (third as a minimum)academic staff engaged in research or other forms of advanced scholarship<br><br>The outcomes of external scrutiny exercises undertaken by bodies such as the Quality Assurance Agency for Higher Education, the funding councils and professional and statutory bodies are carefully considered and actioned.
**External promotion of research**
Collaborations exist with external organisations (institutions, businesses, government, NGO's)<br><br>The institution is able to conform to the requirements of multiple funding agencies<br><br>Number of joint posts with other academic institutions<br><br>The institution has a clear strategy in place for all forms of intellectual property management<br><br>Clear regulations are in place to determine the ownership of intellectual property by and between staff, students and third parties. These regulations are effectively disseminated throughout the institution and externally<br><br>Academic departments and research projects are systematically monitored to identify emerging intellectual property at an early stage.<br><br>The institution establishes a register of intellectual property assets and pro-actively manages and maintains it at all stages of development and exploitation<br><br>Clear policy mechanisms are in place to govern the distribution of revenues from intellectual property between the university and other key stakeholders.<br><br>The institution's research communication strategy is consistent with the institution's overall strategy and underpins the core missions of the institution, particularly in relation to the integration of research, education and service.<br><br>There is a clear understanding of the roles and responsibilities of the different offices/officers responsible for research communication and good channels of communication exist between all these actors.<br><br>The institution pro-actively identifies projects (at various stages) and outcomes that are aligned with the university's priorities and are particularly suitable for external dissemination<br><br>The institution has a programme of events, such as launches, to profile major achievements or projects which relate to the strategic objectives and any priority research themes of the institution.<br><br>The institution has clear criteria for the type of work most likely to generate good publicity, and guidance on how to avoid poor publicity, and makes this information available to staff.<br><br>The institution has a clear strategy and procedures with regard to handling crisis communications and ensures these are disseminated to every level.<br><br>The institution seeks to make key research findings accessible to a wider audience, through the use of research summaries, expert guides and speakers lists, produced in suitable lay language and in

For peer review only

publicly accessible formats so as to engage public understanding of the core mission of the institution (including inter-institutional partnerships).<br><br>The institution has established clear mechanisms to review and reward the performance of departments and research groups in the area of dissemination, which are integrated with an incentivisation policy providing a variety of incentives.<br><br>Mechanisms are in place for staff to report their dissemination activity. Such mechanisms maximise research kudos and academic excellence and are consistent with any reporting requirements to external organisations<br><br>The institution provides assistance and systematic training programmes for staff in handling the media, and specific assistance in the drafting of press releases and publicity materials.<br><br>The institution facilitates the participation of researchers, particularly early career researchers, in international conferences and other fora to present their research findings and raise their profile<br><br>Where possible, dissemination outputs of staff are captured in a centrally managed integrated digital repository, linked to any central research activity database, which is made available to all units of the institution<br><br>The institution has a clear branding policy which is consistent with the research communication strategy.<br><br>The institution's web portal reflects the institution's core mission and strategy and is strategically and systematically managed as a key tool for promoting research to the broader community.
**National Research Uptake**
Ability of link policy to research and practice<br><br>Number of evidence based policies<br><br>Number of evidence based development interventions<br><br>Number of plans and policies to support research<br><br>Existence of national standards (accreditation, quality assurance) regarding the evaluation of research institutes<br><br>Existence of scientific councils with transparent and efficient systems in place to evaluate and disburse competitive research funds<br><br>Level of funding of research by the government<br><br>Researcher salary on par or above other countries in region

For peer review only

Box 3. Overall semi-structured interview suite of questions, by RMSS component

Biography of interviewee

What is your current position within this institution?

How long have you held this position?

How long have you worked at this institution?

What is your role in research within this institution?

1. Research strategies and policies

The institution

How many staff and students are there at this institution?

What is the percentage of income from a) teaching and b) research?

Is there core funding for research? How much? How is it disbursed?

How many PhD students are registered a) with your institution and b) externally?

Is there a university officer/directorate responsible for research? Do they have terms of reference?

How does this institution's research outputs compare to other comparable institutions? How do you measure this?

Strategies

Do you have a university research strategy?

What are the main themes/components of the strategy?

Does it link to a) national and b) other institutional strategies?

How is it disseminated internally and externally?

What are the research strengths at this institution?

Are strategies revised? How often? What were any major changes?

What are the strategic priority research areas? How were they decided? How are researchers and externally funded projects encouraged to focus on these areas?

Was any baseline information (e.g. a SWOT analysis) used to inform the strategy?

Who was involved in setting the strategy? What was the process?

For peer review only

2. Institutional Support Services and Infrastructure

Research management

Is there a university research committee? What do they do?

Is there a research support office? What do they do? (E.g. identify opportunities, help with application process, and ensure compliance with funder's requirements)

How are you made aware of research funding opportunities? (at national, international and regional levels)

How do you keep track of publications/presentations/conferences/grant applications produced/ department?

ICT (also see data management in section 4)

Is there adequate Wi-Fi, broadband speed, video conferencing and skype facilities for researchers? Do they pay for this?

Can they access the IT systems from home?

Do you purchase computers etc. on their behalf, or make recommendations? Do you set them up? Help with software? Is there any charge for this service?

How are files and information backed up? (e.g. offsite servers)

Library

How do staff and students access peer reviewed and grey literature? Are there any regular training courses offered?

How is access to e-resources and hard copy books/journals managed between the ICT unit and the library?

Laboratories

What research laboratories and field sites are available to use for research purposes at this institution?

What type of research studies can be supported by the laboratories (e.g. HPLC for pharmacokinetics; genomics/sequencing; insectory etc.)

Are the laboratories enrolled in external quality assurance systems?

Do the laboratories have international accreditation?

Do the laboratories follow Good Laboratory Practice guidelines?

Are there backup generators? Surge protection?

What sample storage facilities do you have? Are they temperature controlled and monitored?

What are the policies and processes governing transfer of samples to external institutions?

3. Supporting funding applications

For peer review only

What are the mechanisms for identifying external funding opportunities? Does the research office help with this? (E.g. is there a 'grants identification' officer?)

Is there any support to help PIs prepare funding proposals? (E.g. getting documents together, preparing/checking budgets, submitting proposals)

What is the mechanism for collating information on all proposals submitted? Is there a searchable database of submitted projects and whether they were successful?

What is the process for submitting proposals? Is there a formal sign off and if so by whom?

Do proposals have to have input or approval from finance/accountants prior to submission? What do they look for? How do you make sure that overheads are included and the costings are correct (e.g. salaries, equipment)?

Does the university use external advice (e.g. legal) at any stage during the process?

Do you have any way of comparing your research performance with other institutions?

4. Project management and control

What systems are in place to monitor the progress of each project? (E.g. against milestones)

Ethics

How is this managed in the university as a whole? Is this done at the university or at the faculty level?

Is ethics committee membership GCP-compliant?

Are there guidelines about how the ethics committee functions?

Are there guidelines for researchers about the ethics process?

Are there guidelines relating to academic honesty and plagiarism?

Financial

Who provides financial reports to funders? Who has specialist knowledge of each funders' reporting requirements?

How often are financial reports made to PI's (frequency, method, feedback loop?)

How do departments predict and plan future research revenue and expenditure?

How does the university ensure that project expenditure remains in line with the budget?

Legal

What is the process for minimising risks regarding financial and contractual terms? Is legal advice available? Who accesses this and when? (ie during the contract signing process or only if there is a problem)?

For peer review only

How are appropriate insurance arrangements organised (particularly for field staff and clinical trials)

What regulations are in place to determine the ownership of intellectual property by and between staff, students and third parties? How are these regulations disseminated throughout the institution and externally?

If what ways do you identify emerging intellectual property in your academic departments and on-going research projects?

Have you established a register of intellectual property assets? How are these managed and maintained?

What policies/mechanisms are in place to govern the distribution of revenues from intellectual property between the university and other key stakeholders?

Data management

Are there research data management guidelines and/or policies for data protection and storage?

How is research data backed up and secured? How are routine office and research documents (e.g. draft publications, guidelines/protocols etc.) backed up and secured?

Who is responsible for these systems? Are PIs charged for this service?

Do you provide help for PIs to complete Data Management plans to funders?

What are the mechanisms for managing data ownership, data security, licensing for re-use, data sharing, reuse of third-party data, restriction of data sharing (prior to publishing or seeking patents, retaining/destroying data?

Clinical work/trials questions:

Does the university acts as a sponsor for clinical trials?

Is there a clinical trials office? What does it do?

How do you do clinical monitoring? Have any audits about this been conducted and if so what were the key findings?

5. Human Resource Management for Research

Are job descriptions available for researchers and support staff?

Is there an induction process for new employees?

What are the processes for promotion for a) researchers and b) support staff (e.g. administrators, laboratory scientists)?

What mentorship and supervision structures exist for students and early career stage researchers and new PI’s?

For peer review only

What career pathways are there are for a) researchers and b) research support staff?

Is there any ongoing professional development programme and does this cover research skills?

Do you know about the MCDC career development groups (CDGs)?

How do the MCDC career development groups (CDGs) fit into/complement institutional systems?

Are these career development activities embedded in institutional structures?

Do you think they are helpful? Should they be institutionalised? Why/why not?

Human resources

What Policies/strategies are in place for Human resource development of a) researchers/scientific staff, b) admin staff (including training, retention, tenure track, funding)

Do you have a formal induction process for new employees? Is there a special one for researchers?

Are there health and safety policies? (E.g. staff induction, safety officers, evacuation procedures etc.)

How are training needs identified? (E.g. staff training needs assessments). Is HR responsible for providing and/or recording any research training (e.g. GCP/GLP training, proposal writing, project management, supervision)?

How are training opportunities identified and funded? Is there a core budget for training and how is it allocated?

What proportion of research posts are a) core funded and b) project funded?

Are you involved in all new appointments? Do you advise PIs on the institution’s procedures governing the employment of staff?

Is career guidance given to PhD students, post-docs and other researchers?

How are post-docs absorbed into the workforce?

What is the process and turnaround time for recruiting and appointing new research staff?

Do you make more internal or external research appointments?

Does your institution offer the possibility of short-term bridging funding to retain research staff during hiatus periods between grants?

Can you describe what Performance measures are used for research management and how these are reported?

Are there joint posts with other academic institutions? How do they work and are they effective?

6. Human Resource Development for Research

For peer review only

Is training available on

- Research design (epidemiology, stats, social science, health systems)
- Ethics, health and safety, GCP and GLP
- Data analysis and management (including software and qualitative analysis)
- Academic writing and publishing
- Proposal writing, grant application
- Teaching and education
- Leadership and management

Are there facilities and fora (e.g. seminars, journal club, staff exchanges) for researchers to discuss their work regularly with each other?

Is a tracking system in place for PhD students? How many supervisors do PhD students have? How many students do PhD supervisors have?

Are there minimum standards in place about the level of supervision to be given?

7. External promotion of research

Do you have longstanding research collaborations with external organisations? Examples?

Do you have a research communication strategy? A research communication unit?

Who is responsible for research communication? Do researchers perceive that this is their responsibility?

Does the institution have a research profile on its website?

How do you make key research findings accessible to a non-academic audience (e.g. research summaries in lay language and in publicly accessible formats)?

Do you have a programme of events, such as launches, to profile major achievements or projects

Do you provide advice to staff about how to deal with the media (e.g. how to generate good publicity and avoid poor publicity)?

What strategy and procedures are communicated to staff with regard to handling crisis communications and how are these disseminated?

Are there incentives for departments and research groups in the area of dissemination?

8. National research engagement

What level of funding for research is provided by the government?

Are there national policies and plans regarding health (and other) research?

For peer review only

How does the institution engage with policy makers?

Are there national scientific councils that evaluate and disburse competitive research funds? Do they have transparent and efficient systems?

What are the mechanisms by which research from your institution influences policy and practice?

Are there national standards (accreditation, quality assurance) regarding the evaluation of research institutes?

For peer review only
